# Synergistic roles of aquaporin 5 and intra‐ and extracellular carbonic anhydrases in promoting CO_2_ diffusion across the *Xenopus* oocyte plasma membrane

**DOI:** 10.1113/JP289145

**Published:** 2025-12-23

**Authors:** Deng‐Ke Wang, Fraser J. Moss, Walter F. Boron

**Affiliations:** ^1^ Department of Physiology and Biophysics Case Western Reserve University School of Medicine Cleveland Ohio USA; ^2^ Department of Medicine Case Western Reserve University School of Medicine Cleveland Ohio USA; ^3^ Department of Biochemistry Case Western Reserve University School of Medicine Cleveland Ohio USA

**Keywords:** cell‐surface pH, Fick's law, gas channels, intracellular pH, pH‐sensitive microelectrodes

## Abstract

**Abstract:**

CO_2_ diffusion across plasma membranes depends on both membrane CO_2_ permeability ($P_{M,CO_{2}}$) and the transmembrane CO_2_ concentration gradient (Δ[CO_2_]) – Fick's law. Human aquaporin‐5 (hAQP5) enhances CO_2_ diffusion by increasing $P_{M,CO_{2}}$, whereas carbonic anhydrases (CAs) do so by enhancing CO_2_ consumption/production and thus Δ[CO_2_]. Here we systematically assess functional interactions among a gas channel and intra‐/extracellular CAs. On Day 1 we inject *Xenopus* oocytes with cRNA encoding hAQP5 (control: H_2_O). On Day 4 we inject hCA II protein in ‘Tris’ buffer (control: ‘Tris’). We assess CO_2_ fluxes by introducing extracellular 1.5% CO_2_/10 mM HCO_3_^−^ and using microelectrodes to measure (1) the maximal increase of extracellular surface pH (ΔpH_S_), (2) the maximal rate of pH_S_ relaxation (dpH_S_/dt)_Max_ and (3) the maximal rate of intracellular pH decrease (dpH_i_/dt)_Max_. By itself hCA II minimally increases ΔpH_S_ – measured on the side of the membrane opposite to the added cytosolic CA (CA_i_) – even at our highest doses (100 ng/oocyte). However hAQP5 alone triples ΔpH_S_, an effect further doubled by increasing hCA II. By itself bovine erythrocyte CA (bCA) in the extracellular fluid doubles (dpH_i_/dt)_Max_ magnitude – measured on the side of the membrane opposite to the added extracellular CA (CA_o_) – an effect further doubled by hAQP5. Our pH measurements (1) confirm synergy between CA_o_ and CA_i_; establish synergy between hAQP5 and both (2) CA_o_ and (3) CA_i_; and (4) show that the ability of CA_i_ to enhance ΔpH_S_ is a useful tool for assessing the CO_2_ permeability of membrane proteins (e.g. hAQP5).

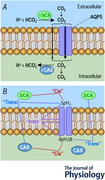

**Key points:**

- According to Fick's law transmembrane CO_2_ flux ($J_{CO_{2}}$) is the product of membrane permeability ($P_{M,CO_{2}}$) and transmembrane concentration gradient (Δ[CO_2_]): $J_{CO_{2}}$ = $P_{M,CO_{2}}$×Δ[CO_2_]. Previous work separately showed that (1) human aquaporin‐5 (hAQP5) enhances $P_{M,CO_{2}}$, and (2) intracellular and (3) extracellular carbonic anhydrases (CAs) enhance (Δ[CO_2_]) by consuming accumulated or replenishing lost CO_2_. We now examine interactions among #1–#3.
- We assess CO_2_ fluxes – produced by addition/removal of extracellular CO_2_/HCO_3_^−^ – using microelectrodes to monitor extracellular surface pH (pH_S_) and intracellular pH (pH_i_) of *Xenopus* oocytes heterologously expressing hAQP5, injected with human CA II (hCA II), and/or exposed to extracellular bovine CA (bCA).
- Enhancing effects on CO_2_ fluxes are synergistic among hAQP5, hCA II and bCA, any of which can become rate‐limiting, depending on the status of the other two.
- CO_2_/HCO_3_^−^ addition transiently increases pH_S_ (ΔpH_S_), hCA II augments ΔpH_S_ (ΔΔpH_S_) and hAQP5 enhances ΔΔpH_S_ (ΔΔΔpH_S_) – a novel tool to assess potential CO_2_ channels.

## Graphical abstract

**Abstract figure legend** Fick's law: 
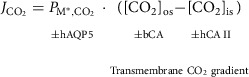

Fick's law governs the diffusion of CO_2_ into a cell. The entry of CO_2_ depletes CO_2_ at the outer surface but raises it at the inner surface, both tending to decrease the transmembrane gradient. A, we show that bovine carbonic anhydrase (bCA) in the extracellular fluid and human CA II in the intracellular fluid increase the flux by enhancing the gradient, whereas aquaporin 5 (AQP5) increases the flux by increasing membrane CO_2_ permeability ($P_{M^{*},CO_{2}}$). The three effects are synergistic. *B*, we assess the CO_2_ flux from pH changes on the cell surface (ΔpH_S_) or intracellular fluid (dpH_i_/dt). However it is critical that one use pH changes that are ‘trans’ to the added CA to avoid confusion from the ‘cis‐side’ consumption or production of H^+^. AQP5 is ‘inter’ with respect to both pH measurements and is thus not subject to ‘cis’ errors.

## Introduction

Aquaporins (AQPs) are integral membrane proteins named for their ability to conduct H_2_O across biological membranes. The AQPs occur ubiquitously across all kingdoms of life (Ishibashi et al., 2017; King et al., 2004; Maurel et al., 2015; Ni et al., 2017). To date the cDNAs encoding 13 human AQPs have been cloned and characterized (for review see Li & Wang, 2017). One can divide them into three groups, based on whether they can conduct glycerol in addition to H_2_O. The first group – the classical aquaporins – comprises AQP0, 1, 2, 4, 5, 6 and 8. Although some classical aquaporins are also permeable to urea (AQP6 and AQP8) or ions (AQP6), all of them have a high permeability to H_2_O. The second group – the aquaglyceroporins – includes AQP3, 7, 9 and 10 (Heymann & Engel, 1999; Ishibashi et al., 1997; Michalek, 2016; Moss et al., 2020; Zhang et al., 2019). Besides H_2_O they can mediate the diffusion of glycerol, urea and other non‐volatile solutes. Finally the third group consists of AQP11 and AQP12, which show the lowest homology to the other aquaporins and have uncertain functions (Calvanese et al., 2013; Ishibashi, 2009).

In addition to conducting H_2_O, glycerol, urea, and other small solutes some AQPs serve as conduits for highly volatile solutes CO_2_ and NH_3_ (for review see Boron, 2010), NO (Herrera et al., 2006; Herrera & Garvin, 2007) and probably O_2_ (Moss et al., 2025; Occhipinti et al., 2025; Zhao et al., 2025). Nakhoul et al. (1998) and Cooper and Boron (1998) used the rate of intracellular acidification (dpH_i_/dt) to examine the effects of expressing human AQP1 on CO_2_ diffusion across the plasma membrane of *Xenopus laevis* oocytes. They were the first to observe that a membrane protein can act as a channel for a dissolved gas. Later Endeward et al. (2006) confirmed the CO_2_ permeability of AQP1 in human red blood cells, using mass spectrometry to monitor the disappearance of C^18^O^16^O. As part of the Endeward study Musa‐Aziz introduced the technique of using the rapid, transient increase of surface pH (pH_S_) – during the application of CO_2_/HCO_3_^−^ – as a semiquantitative index of the CO_2_ influx across the oocyte membrane. This pH_S_ approach is technically simpler than the others, and its use led to the elucidation of the roles that several other AQPs – including AQP5 – play as CO_2_ channels (Geyer et al., 2013; Musa‐Aziz et al., 2009). Mathematical modelling has provided quantitative insight into the events surrounding CO_2_ influx, including the time course of pH_S_ (Calvetti et al., 2020; Musa‐Aziz et al., 2014a, 2014b; Occhipinti et al., 2014; Somersalo et al., 2012).

Raina et al. (1995) were the first to clone the cDNA encoding AQP5, obtaining the clone from a library of rat submandibular gland. Others localized AQP5 to the apical membrane of acinar cells in secretory glands, such as salivary glands (Gresz et al., 2001; Larsen et al., 2011; Ma et al., 1999; Nielsen et al., 1997; Steinfeld et al., 2001; Yoshimura et al., 2016) and lacrimal glands (Ishida et al., 1997; Tsubota et al., 2001). AQP5 also is present in the apical membranes of secretory cells of pyloric glands and duodenal glands (Matsuzaki et al., 2003; Parvin et al., 2002), as well as sweat glands (Nejsum et al., 2002). In the lung AQP5 in rats and humans is localized to apical membrane of alveolar type I pneumocytes but not type II pneumocytes (Funaki et al., 1998; Kreda et al., 2001; Nielsen et al., 1997). However in mice AQP5 is present in the apical membranes of both type I and type II pneumocytes (Krane et al., 2001; Matsuzaki et al., 2009). In the kidney AQP5 co‐localizes with pendrin at the apical membrane of β‐intercalated cells (β‐ICs) that secrete HCO_3_^−^ into the lumen of cortical collecting duct (Procino et al., 2011). In the aforementioned cells AQP5 presumably plays an important role in transepithelial fluid and electrolyte transport either because of its role as a H_2_O channel or, perhaps in the case of β‐intercalated cells, as a CO_2_ channel that promotes HCO_3_^−^ secretion.

Besides the above glandular tissues AQP5 is present in astrocytes, where it may play a role during metabolic and traumatic injuries (Chai et al., 2013). In the eye AQP5 is present in the epithelia layer (Funaki et al., 1998) and stromal keratocytes (Kumari et al., 2012) of the cornea, as well as in the epithelial and fibre cells of the lens (Kumari et al., 2012). In the palmar epidermis AQP5 is expressed in keratinocytes (Blaydon et al., 2013). In the aforementioned cells and elsewhere in the body AQP5 could play a role in cell‐volume regulation and osmo‐sensing.

Governing CO_2_ diffusion across a membrane is Fick's law, a simplified and integrated form of which was introduced by Wroblewski (1879):

(1)

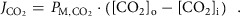

Here $J_{CO_{2}}$ is flux across the membrane; the $P_{M,CO_{2}}$ is the macroscopic membrane CO_2_ permeability; and the term in parentheses indicates the transmembrane CO_2_ concentration gradient expressed in terms of bulk extracellular and intracellular.

For our purposes it is more informative to consider events immediately adjacent to the membrane:

(2)

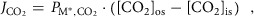

where [CO_2_]_os_ refers to a thin film of aqueous solution on the outer surface of the plasma membrane, [CO_2_]_is_ refers to a thin film on the inner surface and $P_{M^{*},CO_{2}}$ (with an *) is the corresponding permeability.

Equation (2) better reflects the reaction‐diffusion mathematical models to which we refer above. Stated simply $J_{CO_{2}}$ depends both on $P_{M^{*},CO_{2}}$, which can be augmented by certain AQPs and other protein channels, and factors that can influence the nanoscopic concentration gradient near the membrane. Among these latter factors are the carbonic anhydrases (CAs), enzymes that reversibly catalyse the hydration of CO_2_ and the dehydration of H_2_CO_3_ (for review see Boone et al., 2014). Among the 12 enzymatically active human (h) α‐carbonic anhydrases (hCA), hCA II has one of the highest catalytic rates (see Purkerson & Schwartz, 2007). It is expressed broadly throughout the body and is especially abundant in cells that engage in substantial CO_2_/HCO_3_^−^ transport, including red blood cells, the renal proximal tubule and alveolar type I cells (Chen et al., 2008). Moreover CA II appears to play a role in CO_2_ elimination by the lung (Heming et al., 1986; Lien & Lai, 1998; Taki et al., 1999).

A long‐established role of CAs is in the facilitated diffusion of CO_2_ within a single compartment (for review see Occhipinti & Boron, 2019). In the case of transmembrane CO_2_ diffusion, when CA is present on both sides of an artificial membrane, the CA can replenish depleted CO_2_ on the membrane surface from which CO_2_ departs and consume newly arriving CO_2_ on the opposite surface. The result is a magnification of the transmembrane CO_2_ concentration gradient on a nanoscale (see eqn (2)), and thus an increase in transmembrane CO_2_ diffusion (Gutknecht et al., 1977). Work from our group on *Xenopus* oocytes – in the absence of exogenously expressed CO_2_ channels – has shown that hCA II (in the cytosol) and CA IV (mainly on the extracellular surface) can each enhance transmembrane CO_2_ fluxes, and that together the two enzymes produce a supra‐additive stimulation synergism (Musa‐Aziz et al., 2014a, 2014b; Occhipinti et al., 2014). Moreover the group quantitatively accounted for these effects using computer simulations based on three‐dimensional reaction‐diffusion models. Left unanswered is the question of whether CAs can enhance the effect of CO_2_ channels (the $P_{M^{*},CO_{2}}$ term in eqn (2)), and vice versa, on transmembrane CO_2_ fluxes. In the present study we use simultaneous measurements of pH_S_ and intracellular pH (pH_i_) to determine whether hAQP5, cytosolic hCA II and extracellular bovine CA (bCA; from erythrocytes) augment each other's effects in accentuating CO_2_ fluxes across *Xenopus* oocyte plasma membranes. We find that the effects of expressing hAQP5 are supra‐additive (i.e. synergistic) to those of adding CA to either the intra‐ or extracellular fluid. Moreover in experiments in which we measure the increase in cell‐surface pH (ΔpH_S_) caused by the influx of CO_2_ the introduction of CA to the cytosol augments the pH_S_ increase – a ΔΔpH_S_ – by an amount that CO_2_ channels further augment – a ΔΔΔpH_S_. Thus, this ΔΔΔ approach could be a diagnostic tool for identifying candidate CO_2_ channels.

## Methods

### Ethical approval and animal procedures

The Institutional Animal Care and Use Committee at Case Western Reserve University approved all procedures for the housing and handling of *X. laevis* (PHS Assurance number – D16‐00089 (A3145‐01). Housing, anaesthesia and killing were performed essentially as described by Moss and Boron (2020). To alleviate stress we accommodated a maximum of six frogs, each static 20 gallon aquarium tank, filled with dechlorinated water that was circulated using a charcoal Bio‐Bag aquarium power pump (Tetra, Blacksburg, VA, USA). We partially replaced water in the tank as necessary, and every 90 days we transferred the frogs to a newly cleaned tank, which was half‐filled with water from the previous tank and half‐filled with fresh dechlorinated water. We placed a PVC elbow pipe for environmental enrichment in each tank. We fed the frogs thrice per week with an adult *Xenopus* diet (Zeigler Bros. Inc., Gardners, PA, USA). We sprinkled the food (10 pellets per frog) into the aquarium, and the *Xenopus* were permitted to feed. We used a net to remove surplus food after a few hours.

We anaesthetized the *Xenopus* by immersion in a 0.2% tricaine solution. We then removed the animal from the anaesthetic after it became unresponsive to tactile stimuli. We placed ice into a sterile tray, covered the ice with foil and then the anaesthetized *Xenopus* was placed onto the foil surface to surgically remove the ovaries. We killed the animals before regaining consciousness from anaesthesia through cardiac excision.

### Physiological solutions

Our ND96 solution comprises 93.5 mM NaCl, 2 mM KCl, 1 mM MgCl_2_, 1.8 mM CaCl_2_ and 5 mM Hepes (including ∼2.5 mM Na‐Hepes after titration of solution pH to 7.50 using NaOH). We measured solution pH at room temperature using a benchtop pH meter together with a glass pH electrode with a flowing 3M KCl junction (‘Ross’ electrode, Cat#13‐643‐201, ThermoFisher, Waltham, MA, USA). We adjusted the osmolality to 195±5 mOsm by adding NaCl or H_2_O. Our 1.5% CO_2_/10 mM NaHCO_3_ solution was identical to ND96, except that 10 mM NaHCO_3_ replaced 10 mM NaCl, and we bubbled the solution with 1.5% CO_2_/balance O_2_. Our ND48 – identical to ND96, except contained only 48 mM NaCl – was a hypotonic solution (∼100 mOsm) for the osmotic water permeability coefficient (*P*_f_) assay. The 0 Ca^2+^ normal saline (NRS) solution used during oocyte isolation was as described by Musa‐Aziz et al. (2010).

To study the effect of extracellular carbonic anhydrase on CO_2_ diffusion we dissolved bCA purified from bovine erythrocytes (Cat#C3934, Sigma‐Aldrich, St. Louis, MO, USA) – a mixture of CA I and CA II – in both the ND96 and 1.5% CO_2_/10 mM NaHCO_3_ solutions of an experiment to achieve a concentration of 0.1 mg/ml (Musa‐Aziz et al., 2014b).

OR3 medium, as described by Musa‐Aziz et al. (2010), comprises one sachet of Leibovitz L‐15 (Cat#41300, ThermoFisher), 100 ml penicillin‐streptomycin (Cat#15140, ThermoFisher) and 1.785 g HEPES, all dissolved in ∼1.6 l of deionized H_2_O (dH_2_O), with pH titrated to 7.50 and osmolality adjusted to 195±5 mOsm by adding an appropriate volume of dH_2_O.

We confirmed all solution osmolalities using a Wescor model 5520 osmometer.

### Oocyte preparation

For a description of our approach see Musa‐Aziz et al. (2010) and Parker et al. (2008). Briefly on Day 0 we anaesthetized a frog (female *X. laevis*, NASCO, Fort Atkinson, WI, USA) in 0.2% tricaine (Cat#A5040, Sigma‐Aldrich), surgically removed an ovary from the ventral side of the frog, cut the ovary into small pieces (∼0.5 × 0.5 × 0.5 cm) and washed the pieces ×3 with 0 Ca^2+^ NRS in a 50 ml Falcon tube, using a benchtop end‐over‐end rotator. We then digested the extracellular matrix of the follicles with 2 mg/ml collagenase (Cat#C5138, Sigma‐Aldrich) dissolved in 0 Ca^2+^ NRS, washed ×3 in 0 Ca^2+^ NRS, carefully selected stage V and VI oocytes from the processed batch and then cultured in OR3 medium in an incubator at 18°C for subsequent injection of cRNA.

### cDNA construct encoding human AQP5

The cDNA encoding human AQP5 (GenBank# NM_001651.4), subcloned into pGH19 vector, was the same construct as used by Qin and Boron (2012). After the sequence was confirmed, we linearized the cDNA using Xho I (Cat# R0146, New England Biolabs, Ipswich, MA, USA), transcribed it *in vitro* using a mMESSAGE mMACHINE T7 capped RNA transcription kit (Cat#AM1344, ThermoFisher). We purified the cRNA using an RNeasy MinElute Cleanup Kit (Cat#74204, QIAGEN, Hilden, Germany), eluted the resulting cRNA with DNase/RNase‐free H_2_O, quantified the cRNA concentration by measuring peak absorbance at λ = 260 nm (A_260_) and assessed its purity by measuring the ratio of the λ = 260 nm *vs*. λ = 280 nm absorbance (A_260/280_) using a NanoDrop 2000 UV spectrophotometer (ThermoFisher), and diluted the cRNA into 1000 ng/µl aliquots, which we stored at –80°C.

### Expression in *Xenopus* oocytes

On Day 1 we injected oocytes with 25 ng (25 nl) of cRNA encoding hAQP5 or 25 nl of H_2_O as a control. On Day 4 we separated both cRNA‐injected and H_2_O‐injected oocytes into two subgroups, one pair of subgroups for injection with 25 nl containing 1, 10 or 100 ng of hCA II protein purified from human erythrocytes (Cat #C6165, Sigma‐Aldrich) dissolved in ‘Tris’ (i.e. 50 mM Tris base, titrated with HCl) at pH 7.40, and the other pair of subgroups for injection with 25 nl of ‘Tris’ as a control.

We selected healthy oocytes in three ways: (1) we used only oocytes that appeared healthy when observed under a dissecting microscope (see Musa‐Aziz et al., 2010) while still in the Petri dish. (2) Although membrane potential (*V*_m_; see below) is not a universal indicator of oocyte health, we generally accepted only oocytes in which values were more negative than –40 mV. (3) We discarded oocytes that, as we pushed the pH_S_ electrode against the membrane (under microscopic observation in the experimental chamber), exhibited a subjectively soft membrane (often accompanied by a splotchy distribution of colours).

### Electrophysiological measurements

#### Solution delivery to the chamber

At room temperature we put each solution into two 140 ml piston syringes (Cat#8881114030, Covidien, Dublin, Ireland) and delivered the solution using a dual syringe pump (Cat#55‐2226, Harvard Apparatus, Holliston, MA, USA) at a constant total flow of 4 ml/min. The solutions flowed through Tygon tubing (4.76 mm OD × 1.59 mm ID, Cat#ACF00003, Saint‐Gobain, Courbevoie, France) to minimize the leak of CO_2_. We used custom‐manufactured five‐way valves (Clippard, Cincinnati, OH, USA) actuated by nitrogen pressure to switch solutions between ND96 and 1.5% CO_2_/10 mM NaHCO_3_. We controlled the nitrogen pressure using electrically activated four‐way solenoid valves (R481, Clippard).

#### Construction of microelectrodes

We made *V_m_* electrodes from borosilicate tubing (2.00 mm OD × 1.56 mm ID, with filament; Cat#BF200‐156‐10, Sutter Instrument, Novato, CA, USA), which we pulled on a Sutter puller (model P‐97, Sutter Instrument), and filled with 3 M KCl to achieve a resistance of 0.5 to 1.0 MΩ. For pH_i_ electrodes after pulling we dried the micropipettes at 270°C overnight, treated with *bis*‐di‐(methylamino)‐dimethylsilane (Cat#14755, Sigma‐Aldrich) for 20 min, vented the silane vapours, maintained the silanized micropipettes at 270°C overnight, removed them from the oven, filled the tip with H^+^ ionophore I‐cocktail B (Cat#95293, Sigma‐Aldrich) and then filled with the backfill solution (see Musa‐Aziz et al., 2010). We made pH_S_ electrodes in a fashion as pH_i_ electrodes, except that after pulling the tubing (2.00 mm OD × 1.16 mm ID, without filament; Cat#B200‐116‐10, Sutter Instrument), we used a microforge to break the micropipette tip to give a final inner diameter of 30 µm. The thicker wall for the pH_S_ electrodes produces a better tip break, and the lack of the filament stabilizes the H^+^ cocktail.

#### Measurement of pH_S_ and pH_i_

##### Positioning of electrodes

We measured pH_S_ and pH_i_ as previously described (Musa‐Aziz et al., 2010) and illustrated schematically (Musa‐Aziz et al., 2014a, 2014b; Occhipinti et al., 2014). Briefly after pre‐positioning all electrodes (see below) in the plastic chamber, we introduced an oocyte. The flow of ND96 solution pinned the oocyte against X‐shaped nylon fibres immediately downstream, holding the oocyte in place. The final disposition of the microelectrodes (see below) further stabilized the oocyte. We connected the *V*_m_ electrode to an OC‐725C oocyte clamp (Warner Instruments, Hamden, CT, USA) and the pH_i_ and pH_S_ electrodes to a HiZ‐223 amplifier (Warner Instruments). The electrical ground of the chamber was a bath clamp in which the same OC‐725C as above connected to (a) a platinum wire in the chamber and (b) a reference electrode (identical to a *V_m_* electrode but with its tip broken by dragging on paper) that contacted the ND96 downstream from the oocyte. The reference electrode for the pH_S_ measurements was a longer version of a *V*_m_ microelectrode (i.e. filled with 3M KCl), but with a carefully broken tip, placed downstream from the oocyte, and connected via a plastic electrode holder and calomel half‐cell to a model 750 amplifier (WPI, Sarasota, FL, USA). All of the primary electrical signals fed into custom data‐acquisition hardware ‘Ribbit Box’ built in‐house by Dale Huffman around a LabJack U6Pro device (LabJack, Lakewood, CO, USA) and connected to a Windows‐based computer, controlled by custom software ‘The Frog Whisperer’, written in‐house by Dale Huffman.

##### Subtraction of electrical signals

Our sampling frequency was 3/s. We obtained the pH_i_ value by digitally subtracting (in the computer) the *V_m_* signal (voltage) from the pH_i_‐electrode signal (voltage), and converted the difference to a pH_i_ value using the electrode‐calibration data (see below). We similarly obtained the pH_S_ value by subtracting the signal of pH_S_ reference (calomel) from that of the pH_S_ electrode. The above custom software performed the calculations and displayed the record of all electrical parameters *vs*. time on a computer monitor.

#### Calibration of pH microelectrodes

We calibrated pH microelectrodes in two steps, first in certified pH standards (not appropriate for physiological experiments) and then in one of our physiological solutions that we had titrated to pH 7.50 using a commercial glass electrode (the ‘gold standard’), as described above.^1^

The first time we used a new pH_S_ or pH_i_ electrode – we never used an electrode for more than 1 day – we obtained the electrode slope using the solution‐delivery system (see above) to flow sequentially a pH 6.00 or pH 8.00 buffer (Certified, Cat#SB104‐1 and SB112‐1, ThermoFisher) through the chamber – before adding the oocyte – and measuring the voltage signal as described above. This exercise provided the ‘electrode slope’. We accepted an electrode only if it had a slope of at least 55 mV/pH. We then flushed the chamber with our ND96 solution before adding the oocyte and performed a one‐point calibration for each electrode in ND96. This second exercise provided the ‘electrode offset’ in the physiological solution, assuming the slope to be the same as in the certified pH standards. We assigned to the subtracted pH_S_ or pH_i_ electrode voltage, obtained as described above,^2^ the pH value of 7.50 in the ND96 solution. Because the liquid‐membrane pH electrodes can be sensitive to CO_2_, we repeated this calibration during the experiment in the CO_2_/HCO_3_^−^ solution.

#### Recording of electrophysiological data

In consecutive fashion, with ND96 flowing in the chamber, we impaled the oocyte with a *V_m_* electrode and a pH_i_ electrode and then positioned the tip of the pH_S_ electrode ∼300 µm from the oocyte surface using a precision remote‐controlled micro‐manipulator (Cat#ROE200, Sutter Instrument), all as previously described (Musa‐Aziz et al., 2014a). Although maintaining the flow of ND96, we advanced the pH_S_ electrode tip ∼300 µm to touch the oocyte surface, and an additional 40 µm to create a slight dimple on the oocyte surface. After 1 min, we switched the solution from ND96 to 1.5% CO_2_/10 mM NaHCO_3_. After several minutes, we retracted the pH_S_ electrode 340 µm from the oocyte surface for 1 min to obtain a one‐point calibration of the pH_S_ electrode in the bulk 1.5% CO_2_/10 mM NaHCO_3_ solution. We then readvanced the pH_S_ electrode by 340 µm to resume pH_S_ measurements before switching back to ND96. Finally after pH_S_ had stabilized we retracted the pH_S_ electrode by 340 µm to obtain a one‐point calibration in the bulk ND96 solution at the end of the experiment.

#### Calculation of ΔpH_S_

With its tip in the bulk ND96 solution the pH_S_ electrode detected, by definition, a pH of 7.50 (i.e. the initial ND96 calibration). Once dimpling the membrane with the oocyte exposed to ND96 the pH_S_ electrode – calibrated for ND96 – detected pH_S_. The average pH_S_ over at least the final 30 s – when pH_S_ was stable – we took as pH_S_(ND96_Init_). From the moment that we switched the chamber solution to 1.5% CO_2_/10 mM NaHCO_3_ the initial ND96 calibration of the pH_S_ electrode was no longer valid, which is why we obtained a new one‐point calibration in 1.5% CO_2_/10 mM NaHCO_3_ (see above); we applied this second CO_2_/HCO_3_^−^ calibration to all pH_S_ data obtained during the CO_2_/HCO_3_^−^ exposure, as pH_S_ rises to a peak and then decays downwards. Using this second calibration we computed the peak pH_S_ after the switch to CO_2_/HCO_3_^−^, that is, pH_S_(CO_2,Peak_). We define the ‘upward’ ΔpH_S_ for CO_2_ addition as pH_S_(CO_2,Peak_) – pH_S_(ND96_Init_). At the end of the CO_2_/HCO_3_^−^ exposure the average pH_S_ over at least the final 30 s – when pH_S_ was stable – we took as pH_S_(CO_2,Tail_). From the moment that we switched the chamber solution to ND96 the previous 1.5% CO_2_/10 mM NaHCO_3_ calibration of the pH_S_ electrode was no longer valid, which is why we obtained a final (i.e. third) one‐point calibration in ND96 (see above); we applied this final ND96 calibration to all pH_S_ data obtained during the final ND96 exposure, as pH_S_ falls to a trough and then decays upwards. Using this third calibration we computed the lowest pH_S_ after the switch to ND96, namely, pH_S_(ND96_Nadir_). We define the ‘downward’ ∆pH_S_ for CO_2_ removal as pH_S_(ND96_Nadir_) – pH_S_(CO_2,Tail_), which is a negative number.

#### Calculation of ‘maximal’ dpH_S_/dt

**Figure 1:**
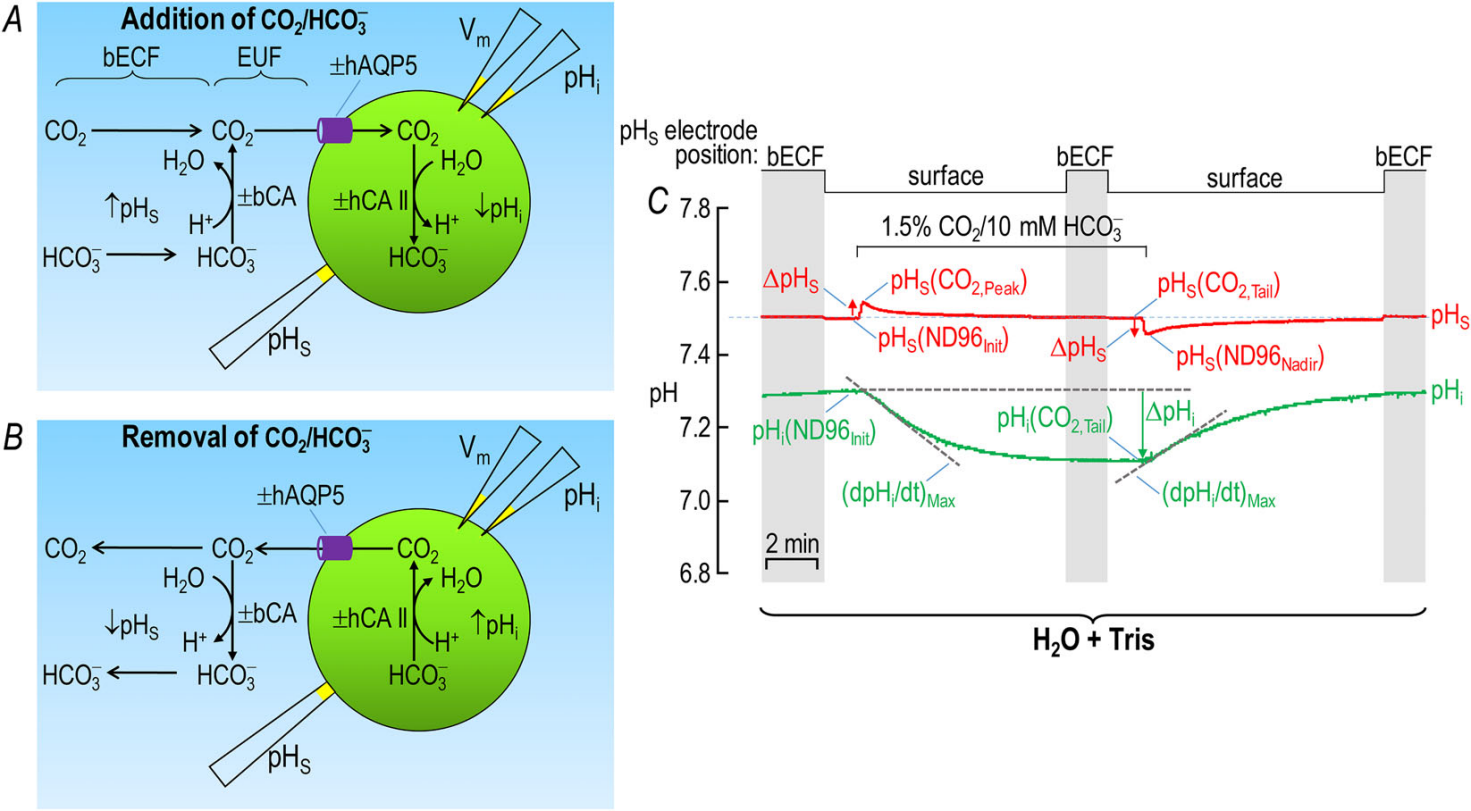
Design of experiments A, model cell showing CO_2_ influx. The green circle represents a *Xenopus laevis* oocyte. Arrows with flared arrowheads indicate the direction of diffusion. Arrows with triangular arrowheads indicate the direction of a chemical reaction. Upward and downward arrows associated with pH_S_ and pH_i_ indicate the direction of a pH change. The purple cylinder represents a human (h) AQP5 tetramer heterologously expressed in the membrane. We impale a *X. laevis* oocyte with both a pH_i_ and *V_m_* electrode and used a blunt, polished electrode to monitor pH_S_. ±, presence or absence; bECF, bulk extracellular fluid; bCA, extracellular bovine carbonic anhydrase (a mixture of CA I and CA II); EUF, extracellular unconvected fluid; hCA II, intracellular purified carbonic anhydrase II protein from human erythrocytes. B, model cell showing CO_2_ efflux. All events occur in a direction opposite to that in *A*. C, representative experiment. We dissected the oocyte on Day 0, injected it with H_2_O (as a control for cRNA encoding hAQP5) on Day 1 and then with ‘Tris’ (as a control for hCA II protein) on Day 4. The stair‐step line at the top of the panel indicates the position of the tip of the pH_S_ electrode. The three vertical shaded bars indicate the time during which the pH_S_‐electrode tip is in the bECF for a 1‐point recalibration at pH_o_ = 7.50; at other times the pH_S_‐electrode tip dimples the cell surface. We used the first and third recalibration periods for assignment of pH_S_ values for times that the oocyte was exposed to ND96, and the middle recalibration period for assignment of pH_S_ values for times that the oocyte was exposed to CO_2_/HCO_3_^−^. The flowing solution is ND96, except for the period labelled ‘1.5 CO_2_/10 mM HCO_3_^−^’. The red record represents the time course of pH_S_, and the green record represents the time course of pH_i_. We provide detailed definitions of the red and green labels in Methods.^3^

During CO_2_/HCO_3_^−^ addition (e.g. see Fig. 1*C*) pH_S_ rises rapidly from around 7.50 to a peak (where dpH_S_/dt ≅ 0) and then begins to decline, at first slowly (dpH_S_/dt is slightly negative), later rapidly (dpH_S_/dt reaches a maximally negative value) and finally more slowly again (dpH_S_/dt gradually becomes less and less negative) as pH_S_ relaxes towards an asymptotic value around 7.50. During CO_2_/HCO_3_^−^ removal the changes are in the opposite direction. Here we describe the analysis for CO_2_/HCO_3_^−^ addition, but the approach is similar for CO_2_/HCO_3_^−^ removal. Using software written in‐house by Dale Huffman we obtain a running linear fit of (pH_S_, time) data points to generate a plot of (dpH_S_/dt)_Running_ *vs*. time, which allows us to identify the time (which we define as local time: *t*_Local_ = 0) of the maximally negative dpH_S_/dt (i.e. minimal dpH_S_/dt). We then obtain a double‐exponential (DExp) fit of the (pH_S_, time) data points, beginning at *t*_Local_ = 0 and extending to the time when we removed the pH_S_ electrode from the cell surface to the bulk extracellular fluid (bECF) (for calibration). Finally we evaluate the derivative of the DExp function at *t*_Local_ = 0 and take this value as (dpH_S_/dt)_Max_. During CO_2_/HCO_3_^−^ addition this value is negative (i.e. maximal rate of pH_S_ descent), and during CO_2_/HCO_3_^−^ removal (e.g. see Fig. 1*C*) this value is positive (i.e. maximal rate of pH_S_ rise).

#### Calculation of ‘maximal’ dpH_i_/dt

After either the addition or removal of CO_2_/HCO_3_^−^ we define (dpH_i_/dt)_Max_ as the fastest change in pH_i_, which occurs several seconds after the new solution reaches the oocytes (Musa‐Aziz et al., 2014a). For CO_2_/HCO_3_^−^ addition pH_i_ decreases and thus the ‘downward’ (dpH_i_/dt)_Max_ is a negative number; upon CO_2_/HCO_3_^−^ removal the ‘upward’ (dpH_i_/dt)_Max_ is positive. After the time of the fastest pH_i_ change was visually identified (i.e. corresponding to the most extreme local value of dpH_i_/dt) we used Origin 2024 to obtain (dpH_i_/dt)_Max_ by a linear fit over the next ∼10 s of (pH_i_, time) data.

#### Calculation of intrinsic intracellular buffering power

We defined initial pH_i_ in ND96 solution – pH_i_(ND96_Init_) – as the average of at least 90 data points over a period (i.e. 30 s) when the experimenter (in real experimental time) judged pH_i_ to be stable just before switching from ND96 to the CO_2_/HCO_3_^−^ solution. Similarly we defined the final pH_i_ in the CO_2_/HCO_3_^−^ solution – pH_i_(CO_2_,_Tail_) – as the average of at least 90 data points over a period when the experimenter (in real experimental time) judged pH_i_ appeared stable just before switching from the CO_2_/HCO_3_^−^ solution to ND96. During CO_2_/HCO_3_^−^ application we defined ΔpH_i_ (a negative number) as pH_i_(CO_2_,_Tail_) – pH_i_(ND96_Init_).

Based on the decrease in steady‐state pH_i_ produced by the aforementioned application of CO_2_/HCO_3_^−^ we calculated the intrinsic intracellular buffering (Boron, 1977) power β_I_ (mM/pH) of oocytes using the following equation:


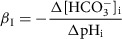




Here the negative sign arises because we are observing a pH_i_ decrease produced by an intracellular, CO_2_‐induced acid load. Because we impose the acid load by the introduction of CO_2_/HCO_3_^−^, followed by the intracellular reaction CO_2_ + H_2_O → H^+^ + HCO_3_^−^, the magnitude of the acid load is Δ[HCO_3_^−^]_i_ (for a discussion see Roos & Boron, 1981). Because we assume that [HCO_3_^−^]_i_ is 0 before the exposure to CO_2_/HCO_3_^−^, ∆[HCO_3_^−^]_i_ (a positive number) is the same as the final [HCO_3_^−^]_i_ after the introduction of CO_2_/HCO_3_^−^, determined at a time when pH_i_ is stable. Because we observed no pH_i_ recovery during the CO_2_/HCO_3_^−^ exposure, nor a pH_i_ overshoot after the removal of CO_2_/HCO_3_^−^, we can conclude that net acid extrusion during the CO_2_/HCO_3_^−^ exposure was negligible (Boron & De Weer, 1976). Thus we computed the final [HCO_3_^−^]_i_ using pH_i_(CO_2_,_Tail_). Because we used 1.5% CO_2_/10 mM HCO_3_^−^ at a pH of 7.5, we can use the shorthand equation:


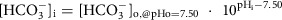

Here we assume that [CO_2_]_i_ = [CO_2_]_o_, where the subscript ‘o’ denotes the bECF, and that the *pK*_a_ in the intracellular fluid is the same as in the extracellular fluid.

### Measurement of *P*_f_

After the pH_S_ and pH_i_ were recorded we saved the oocyte, equilibrated it in ND96 for at least 10 min, transferred it to a Petri dish containing the hypotonic solution ND48 and a 1.6 mm diameter steel sphere (i.e. a ball bearing), placed the dish under a dissecting microscope (model Stemi 508, ZEISS, Oberkochen, Germany) equipped with a video camera (OptixCam summit series, Microscope LLC, Roanoke, VA, USA) connecting to a computer running proprietary software and recorded 1 image/s of the swelling oocyte and the nearby steel sphere for 1 min. We used Image J software (U. S. National Institutes of Health, Bethesda, MA, USA, https://imagej.nih.gov/ij) to determine the perimeter of the oocyte at each time point, computed the projection area (using the steel sphere as an area standard) and the oocyte volume (assuming the oocyte to be a sphere) and used the following equation to calculate osmotic water permeability:

$$P_{f}=\frac{V_{0}\cdot \frac{d(V/V_{0})}{\mathrm{dt}}}{S\cdot \Delta \mathrm{Osm}\cdot V_{w}}$$

Here, *V*_0_ is the initial oocyte volume, *d*(*V*/*V*_0_)/*dt* is the rate of volume increase during the first minute, ∆*Osm* is the osmotic gradient across the membrane (i.e. 195 mOsm – 100 mOsm), *V*_W_ is the molar volume of water (18 ml/molar) and S is the oocyte surface area, assumed to be eightfold greater than the idealized area (Chandy et al., 1997).

### Statistical analysis

We present data as mean ± SD. To compare the difference between two or more groups we use a one‐way ANOVA followed by the Tukey *post hoc* analysis. We perform the analyses using Origin 2024, considering *P* < 0.05 as significant.

To compare means of analysed parameters of oocytes exposed to bECF lacking bCA (grey, black, light‐ and dark‐blue bars in Fig. 3*A–D*) with oocytes for which the bECF contained bCA (Fig. 8*A–D*) we perform a one‐way ANOVA with Tukey's means comparisons. In Statistics Table 8*E‐H* we present descriptive statistics and means comparisons to determine the statistical significance of bCA‐dependent differences.

**Statistics Table 3:** For clarity within the figure panel we present tables of *P*‐values for one‐way ANOVA with Tukey's *post hoc* means comparison for the data presented in Fig. 3. For all tables α is 0.05, and significant *P*‐values are highlighted in bold. A, *P‐*values for means comparisons of ∆pH_S_ upon CO_2_ addition data. B, *P*‐values for means comparisons of ∆pH_S_ upon CO_2_ removal data. C, *P‐*values for means comparisons of (dpH_i_/dt)_Max_ upon CO_2_ addition data. D, *P‐*values for means comparisons of (dpH_i_/dt)_Max_ upon CO_2_ removal data

3A, ∆pH_S_ upon CO_2_ addition								
cRNA		H_2_O	hAQP5	H_2_O	hAQP5	H_2_O	hAQP5	H_2_O
	hCA II (ng)	‘Tris’	‘Tris’	1	1	10	10	100
hAQP5	‘Tris’	**0.00150**						
H_2_O	1	0.956	**0.0391**					
hAQP5	1	**4.18×10^−8^**	**0.00247**	**7.19×10^−8^**				
H_2_O	10	0.943	**0.0456**	1.00	**7.72×10^−8^**			
hAQP5	10	**3.90×10^−8^**	**7.38×10^−4^**	**5.43×10^−8^**	1.00	**5.60×10^−8^**		
H_2_O	100	0.947	**0.0437**	1.00	**7.56×10^−8^**	1.00	**5.55×10^−8^**	
hAQP5	100	**2.21×10^−8^**	**1.34×10^−7^**	**2.93×10^−8^**	0.0978	**2.97×10^−8^**	0.214	**2.95×10^−8^**

3B, ∆pH_S_ upon CO_2_ removal								
cRNA		H_2_O	hAQP5	H_2_O	hAQP5	H_2_O	hAQP5	H_2_O
	hCA II (ng)	‘Tris’	‘Tris’	1	1	10	10	100
hAQP5	‘Tris’	**2.03×10^−4^**						
H_2_O	1	0.992	**0.00309**					
hAQP5	1	**4.44×10^−8^**	**0.0321**	**7.16×10^−8^**				
H_2_O	10	0.995	**0.00249**	1.00	**6.67×10^−8^**			
hAQP5	10	**4.51×10^−8^**	**0.0383**	**7.76×10^−8^**	1.00	**7.15×10^−8^**		
H_2_O	100	0.994	**0.00278**	1.00	**6.91×10^−8^**	1.00	**7.44×10^−8^**	
hAQP5	100	**3.17×10^−8^**	**1.50×10^−4^**	**3.72×10^−8^**	0.692	**3.68×10^−8^**	0.650	**3.70×10^−8^**

3C, (dpH_i_/dt)_Max_ upon CO_2_ addition								
cRNA		H_2_O	hAQP5	H_2_O	hAQP5	H_2_O	hAQP5	H_2_O
	hCA II (ng)	‘Tris’	‘Tris’	1	1	10	10	100
hAQP5	‘Tris’	1.00						
H_2_O	1	0.0803	0.0744					
hAQP5	1	**0.00798**	**0.00726**	0.989				
H_2_O	10	**3.62×10^−4^**	**3.25×10^−4^**	0.624	0.979			
hAQP5	10	**3.76×10^−5^**	**3.36×10^−5^**	0.244	0.753	0.998		
H_2_O	100	**5.69×10^−4^**	**5.10×10^−4^**	0.708	0.991	1.00	0.994	
hAQP5	100	**0.00210**	**0.00189**	0.905	1.00	0.999	0.938	1.00

3D, (dpH_i_/dt)_Max_ upon CO_2_ removal								
cRNA		H_2_O	hAQP5	H_2_O	hAQP5	H_2_O	hAQP5	H_2_O
	hCA II (ng)	‘Tris’	‘Tris’	1	1	10	10	100
hAQP5	‘Tris’	1.00						
H_2_O	1	0.154	0.141					
hAQP5	1	**0.0330**	**0.0295**	0.998				
H_2_O	10	**0.00381**	**0.00335**	0.865	0.995			
hAQP5	10	**4.57×10^−5^**	**3.97×10^−4^**	0.479	0.862	0.998		
H_2_O	100	**0.00160**	**0.00140**	0.721	0.972	1.00	1.00	
hAQP5	100	**0.0460**	**0.0413**	1.00	1.00	0.988	0.801	0.947

**Statistics Table 5:** For clarity within the figure panel we present tables of *P*‐values for one‐way ANOVA with Tukey's *post hoc* means comparison for the data presented in Fig. 5. For all tables α is 0.05, and significant *P*‐values are highlighted in bold. A, *P‐*values for means comparisons of (dpH_S_/dt)_Max_ upon CO_2_ addition. B, *P*‐values for means comparisons of (dpH_S_/dt)_Max_ upon CO_2_ removal. Where the displayed *P*‐values are 0.00, this indicates a values less than <2.22×10^−308^.

*5A, (dpH_S_/dt)_Max_ upon CO_2_ addition*								
cRNA		H_2_O	hAQP5	H_2_O	hAQP5	H_2_O	hAQP5	H_2_O
	hCA II (ng)	‘Tris’	‘Tris’	1	1	10	10	100
hAQP5	‘Tris’	**4.79×10^−4^**						
H_2_O	1	0.998	**0.00518**					
hAQP5	1	**4.73×10^−8^**	**0.104**	**7.84×10^−8^**				
H_2_O	10	0.975	**0.00655**	1.00	**4.77×10^−8^**			
hAQP5	10	**3.42×10^−8^**	**7.41×10^−4^**	**4.18×10^−8^**	0.408	**4.03×10^−8^**		
H_2_O	100	0.993	**0.0134**	1.00	**4.89×10^−7^**	1.00	**4.78×10^−8^**	
hAQP5	100	**2.85×10^−8^**	**0.00**	**2.85×10^−8^**	**4.74×10^−8^**	**0.00**	**3.95×10^−4^**	**0.00**

*5B, (dpH_S_/dt)_Max_ upon CO_2_ removal*								
cRNA		H_2_O	hAQP5	H_2_O	hAQP5	H_2_O	hAQP5	H_2_O
	hCA II (ng)	‘Tris’	‘Tris’	1	1	10	10	100
hAQP5	‘Tris’	**0.0151**						
H_2_O	1	0.999	0.0862					
hAQP5	1	**7.67×10^−4^**	0.998	**0.00783**				
H_2_O	10	0.995	0.0768	1.00	**0.00516**			
AQP5	10	**0.00116**	0.992	**0.0125**	1.00	**0.00975**		
H_2_O	100	0.995	0.165	1.00	**0.00228**	1.00	**0.0303**	
AQP5	100	**3.68×10^−8^**	**0.0114**	**4.03×10^−7^**	**0.0280**	**1.21×10^−7^**	0.144	**2.06×10^−6^**

2.22×10^−308^ is the smallest possible value for double type data that a 64‐bit system is able to distinguish.

**Statistics Table 6:** For clarity within the figure panel we present tables of *P*‐values for one‐way ANOVA with Tukey's *post hoc* means comparison for the data presented in Fig. 6. For all tables α is 0.05, and significant *P*‐values are highlighted in bold. A, *P*‐values for means comparisons of initial pH_i_ data. B, *P*‐values for means comparisons of ∆pH_i_ data. C, *P‐*values for means comparisons of intrinsic buffering power (β_I_). D, *P*‐values for means comparisons of *P*_f_ data

*6A Initial pH_i_*								
cRNA		H_2_O	hAQP5	H_2_O	hAQP5	H_2_O	hAQP5	H_2_O
	hCA II (ng)	‘Tris’	‘Tris’	1	1	10	10	100
hAQP5	‘Tris’	0.701						
H_2_O	1	0.971	0.997					
hAQP5	1	0.512	1.00	0.978				
H_2_O	10	1.00	0.481	0.877	0.309			
hAQP5	10	0.703	1.00	0.998	1.00	0.484		
H_2_O	100	1.00	0.569	0.925	0.385	1.00	0.572	
hAQP5	100	0.997	0.972	1.00	0.898	0.970	0.973	0.987

*6B ∆pH_i_*								
cRNA		H_2_O	hAQP5	H_2_O	hAQP5	H_2_O	hAQP5	H_2_O
	hCA II (ng)	‘Tris’	‘Tris’	1	1	10	10	100
hAQP5	‘Tris’	1.00						
H_2_O	1	1.00	1.00					
hAQP5	1	0.679	0.909	0.757				
H_2_O	10	0.980	1.00	0.991	0.994			
hAQP5	10	0.0737	0.199	0.0995	0.902	0.460		
H_2_O	100	1.00	1.00	1.00	0.748	0.990	0.0963	
hAQP5	100	0.480	0.767	0.563	1.00	0.960	0.976	0.554

*6C β_I_*								
cRNA		H_2_O	hAQP5	H_2_O	hAQP5	H_2_O	hAQP5	H_2_O
	hCA II (ng)	‘Tris’	‘Tris’	1	1	10	10	100
hAQP5	‘Tris’	0.982						
H_2_O	1	0.995	1.00					
hAQP5	1	1.00	0.869	0.934				
H_2_O	10	0.876	0.326	0.434	0.984			
hAQP5	10	0.504	0.0905	0.137	0.782	0.998		
H_2_O	100	1.00	0.928	0.970	1.00	0.960	0.685	
hAQP5	100	0.720	0.188	0.267	0.926	1.00	1.00	0.867

*6D P_f_*								
cRNA		H_2_O	hAQP5	H_2_O	hAQP5	H_2_O	hAQP5	H_2_O
	hCA II (ng)	‘Tris’	‘Tris’	1	1	10	10	100
hAQP5	‘Tris’	**6.86×10^−8^**						
H_2_O	1	1.00	**8.93×10^−7^**					
hAQP5	1	**1.87×10^−6^**	1.00	**2.79×10^−7^**				
H_2_O	10	1.00	**8.76×10^−7^**	1.00	**8.85×10^−7^**			
hAQP5	10	**7.30×10^−8^**	0.988	**7.23×10^−8^**	0.996	**7.16×10^−8^**		
H_2_O	100	1.00	**8.96×10^−7^**	1.00	**6.86×10^−8^**	1.00	**7.25×10^−8^**	
hAQP5	100	**7.08×10^−8^**	1.00	**1.86×10^−6^**	1.00	**8.93×10^−7^**	0.999	**1.87×10^−6^**

**Statistics Table 8:** For clarity within the figure panel we present tables of *P*‐values for one‐way ANOVA with Tukey's *post hoc* means comparison for the data presented in Fig. 8. For all tables α is 0.05, and significant *P*‐values are highlighted in bold. A, *P*‐values for means comparisons of initial pH_i_ data. B, *P*‐values for means comparisons of ∆pH_i_ data. C, *P‐*values for means comparisons of β_I_. D, *P*‐values for means comparisons of *P*_f_ data. Panels E–H provide descriptive statistics and means comparisons (see Methods, Statistical analysis) for comparisons of data represented by the grey, black, light‐blue and dark‐blue bars in Fig. 3 (–bCA) *vs*. Fig. 8 (+bCA). E, ΔpH_i_ during addition of CO_2_/HCO_3_^−^. F, ΔpH_i_ during removal of CO_2_/HCO_3_^−^. G, (dpH_i_/dt)_Max_ during addition of CO_2_/HCO_3_^−^. H, (dpH_i_/dt)_Max_ during addition of CO_2_/HCO_3_^−^

*8A ∆pH_i_ CO_2_ addition*							
cRNA		H_2_O	hAQP5	H_2_O			
	hCA II (ng)	‘Tris’	‘Tris’	1			
hAQP5	‘Tris’	1.00					
H_2_O	1	**0.00582**	**0.00663**				
hAQP5	1	**6.13×10^−4^**	**6.99×10^−4^**	0.757			

*8B ∆pH_i_ CO_2_ removal*							
cRNA		H_2_O	hAQP5	H_2_O			
	hCA II (ng)	‘Tris’	‘Tris’	1			
hAQP5	‘Tris’	0.996					
H_2_O	1	**0.0352**	0.0560				
hAQP5	1	**0.00287**	**0.00478**	0.674			

*8C (dpH_i_/dt)_Max_ CO_2_ addition*							
cRNA		H_2_O	hAQP5	H_2_O			
	hCA II (ng)	‘Tris’	‘Tris’	1			
hAQP5	‘Tris’	**0.0331**					
H_2_O	1	**2.42×10^−6^**	0.00171				
hAQP5	1	**2.33×10^−5^**	0.0192	0.707			

*8D (dpH_i_/dt)_Max_ CO_2_ removal*							
cRNA		H_2_O	hAQP5	H_2_O			
	hCA II (ng)	‘Tris’	‘Tris’	1			
hAQP5	‘Tris’	0.571					
H_2_O	1	**1.42×10^−5^**	**2.49×10^−4^**				
hAQP5	1	**7.08×10^−4^**	**0.0135**	0.318			

*8E, ∆pH_S_ upon CO_2_ addition ±bCA in bECF (*Fig. *3A* *vs*. Fig. *8A*)							
Descriptive stats.	n	Mean	SD	SEM			
bECF	32	0.0689	0.0422	0.00746			
bECF + bCA	24	0.286	0.0641	0.0131			
**Means comparison**	**Mean diff**.	**SEM**	***Q*‐value**	***P*‐value**	**α**	**LCL**	**UCL**
bECF *vs*. bECF + bCA	0.217	0.0142	21.6	**4.53×10^−8^**	0.0500	0.188	0.245

*8F, ∆pH_S_ upon CO_2_ removal ±bCA in bECF (*Fig. *3B* *vs*. Fig. *8B*)							
Descriptive stats.	n	Mean	SD	SEM			
bECF	32	−0.0771	0.0487	0.00861			
bECF + bCA	24	−0.386	0.0681	0.0139			
**Means comparison**	**Mean diff**.	**SEM**	***Q*‐value**	***P*‐value**	**α**	**LCL**	**UCL**
bECF *vs*. bECF + bCA	−0.309	0.0156	28.0	**4.53×10^−8^**	0.0500	−0.340	−0.278

*8G, (dpH_i_/dt)_Max_ upon CO_2_ addition ±bCA in bECF (*Fig. *3C* *vs*. Fig. *8C*)							
Descriptive stats.	n	Mean	SD	SEM			
bECF	32	−0.00315	0.00236	4.18×10^−4^			
bECF + bCA	24	−0.00528	0.00225	4.60×10^−4^			
**Means comparison**	**Mean diff**.	**SEM**	***Q*‐value**	***P*‐value**	**α**	**LCL**	**UCL**
bECF *vs*. bECF + bCA	−0.00213	6.25×10^−4^	4.83	**0.00122**	0.0500	−0.00339	−8.81×10^−4^

*8H, (dpH_i_/dt)_Max_ upon CO_2_ removal ±bCA in bECF (*Fig. *3D* *vs*. Fig. *8D*)							
Descriptive stats.	*n*	Mean	SD	SEM			
bECF	32	0.00183	0.00125	2.22×10^−4^			
bECF + bCA	24	0.00323	0.00161	3.29×10^−4^			
**Means comparison**	**Mean diff**.	**SEM**	***Q*‐value**	***P*‐value**	**α**	**LCL**	**UCL**
bECF *vs*. bECF + bCA	0.00140	3.83×10^−4^	5.17	**5.82×10^−7^**	0.0500	6.32×10^−4^	0.00217

To compare mean values of various other parameters – initial pH_i_, ΔpH_i_, β_I_ and *P*_f_ – between oocytes not exposed to bCA (grey, black, light‐ and dark‐blue bars in Fig. 6*A–D*) and those exposed to bCA (Fig. 10*A–D* (bECF +bCA) we use the same approach described in the previous paragraph. In Table 10E–H we present descriptive statistics and means comparisons to determine the statistical significance of bCA‐dependent differences.

In Fig. 11 we determine the mean ΔΔpH_S,Base_ by subtracting the mean ΔpH_S_ for –hAQP5/–hCA II oocytes from each measured ΔpH_S_ of –hAQP5/+hCA II oocytes. We determined the mean ΔΔpH_S,hAQP5_ by subtracting the mean (ΔpH_S_) for +hAQP5/–hCA II oocytes from each measured ΔpH_S_ of +hAQP5/+hCA II oocytes. We then performed a one‐way ANOVA followed by the Tukey *post hoc* analysis on the data for ΔΔpH_S,Base_ *vs*. ΔΔpH_S,hAQP5_.

In Fig. 12 we determine the mean Δ(dpH_i_/dt)_Max,Base_ by subtracting the mean (dpH_i_/dt)_Max_ for –hAQP5/–bCA oocytes from each measured (dpH_i_/dt)_Max_ of –hAQP5/+bCA oocytes. We determined the mean Δ(dpH_i_/dt)_Max,hAQP5_ by subtracting the mean Δ(dpH_i_/dt)_Max_ for +hAQP5/–bCA oocytes from each measured Δ(dpH_i_/dt)_Max_ of +hAQP5/+bCA oocytes. We then performed a one‐way ANOVA followed by the Tukey *post hoc* analysis on the data for Δ(dpH_i_/dt)_Max,Base_ *vs*. Δ(dpH_i_/dt)_Max,hAQP5_.

## Results

### General protocol

Figure 1*A* and *B* is a schematic representation suggesting how hAQP5, cytosolic hCA II and extracellular bCA would affect the influx (panel A) or efflux of CO_2_ (panel B) and thus pH_S_ and pH_i_ of an *Xenopus* oocyte. Imagine that we switch the solution – the bECF^4^ – that flows through our chamber from one that nominally lacks to one that contains CO_2_/HCO_3_^−^. Imagine also that we have a cell whose membrane is initially impermeable to CO_2_ and HCO_3_^−^. Thus a short time after we switch solutions CO_2_ and HCO_3_^−^ will have diffused throughout the system, so that [CO_2_] and [HCO_3_^−^] will be uniform through the bECF (an ‘infinite reservoir’) and extracellular unconvected fluid (EUF), including a thin layer of fluid at the oocyte surface (S). Suddenly we now increase $P_{M,CO_{2}}$, which is where Fig. 1*A* picks up the narrative. Now CO_2_ diffuses into the cell (Fig. 1*A*), leading to the depletion of CO_2_ at the cell surface. The replenishment of this surface CO_2_ occurs via both CO_2_ diffusion from bECF and – especially adjacent to the cell surface – the reaction HCO_3_^−^ + H^+^ → CO_2_ + H_2_O. The reaction causes a rise in pH_S_, the maximum magnitude of which is ΔpH_S_; assuming that cell‐surface CA activity is fixed ΔpH_S_ is a semiquantitative index of the CO_2_ influx (see eqn (2)). Meanwhile beneath the membrane the CO_2_ that has entered the cell undergoes the reaction CO_2_ + H_2_O → H^+^+ HCO_3_^−^, thus causing a fall in pH_i_. The maximal rate of intracellular acidification – assuming that cytosolic CA activity is fixed – is also an index of the CO_2_ influx. However (dpH_i_/dt)_Max_ is a far less sensitive measure than ΔpH_S_ (Musa‐Aziz et al., 2014b).

If we nominally remove CO_2_/HCO_3_^−^ from the solution flowing through the chamber, all of the above processes, including changes in pH_S_ and pH_i_, reverse (Fig. 1*B*).

Figure 1*C* shows typical pH_S_ and pH_i_ records of a control oocyte – that is, one injected with H_2_O (rather than cDNA dissolved in H_2_O encoding hAQP5) on Day 1 and then ‘Tris’ (rather than hCA II dissolved in ‘Tris’) on Day 4 – during the application and removal of CO_2_/HCO_3_^−^. No bCA is present in the bECF. The shaded vertical bars represent periods of time during which the pH_S_ electrode is ∼300 µm from the cell surface, in the bECF, for electrode recalibration (for details see figure legend and Methods^5^). At other times (i.e. between shaded bars) the electrode tip dimples the oocyte surface. Switching the solution from ND96 to 1.5% CO_2_/10 mM NaHCO_3_ causes an upward pH_S_ transient that soon reaches a maximum (left upward red arrow) and a pH_i_ decrease, the maximum rate of which is indicated by the left dashed line. The pH_S_ signal (after the pH_S_ peak) relaxes and pH_i_ signal declines with similar time constants (Musa‐Aziz et al., 2014a, 2014b; Occhipinti et al., 2014). Later we switch the solution back to ND96, which elicits a reversal of the previous events. In Methods and the figure legend we define the red and green labels shown in Fig. 1*C*.

In the following eight sections we describe the effects – on various parameters measured during our standard protocol in Fig. 1*C* – of various combinations of human AQP5 expressed or not (±hAQP5), human CA II injected or not (±hCA II) and bovine CA added to the bulk extracellular fluid or not (±bCA). In the first four sections we examine oocytes in the absence of bCA but with various combinations of ±hAQP5 and ±hCA II. In the second group of four we study oocytes in the presence of extracellular bCA.

### ±hAQP5^6^ in absence of exogenous CAs: effects on ΔpH_S_ and (dpH_i_/dt)_Max_

Our overall goal is to investigate the relative roles of hAQP5, cytosolic hCA II and extracellular bCA in promoting CO_2_ fluxes across the plasma membrane of a *Xenopus* oocyte. In this first section we begin by verifying previous data that addressed the role of hAQP5 in promoting CO_2_ diffusion in the absence of added CAs. We injected oocytes on Day 1 with either ‘H_2_O’ or H_2_O + cRNA encoding ‘hAQP5’ and then, on Day 4, with ‘Tris’ (as a control for ‘Tris + hCA II’).

#### pH_S_

**Figure 2:**
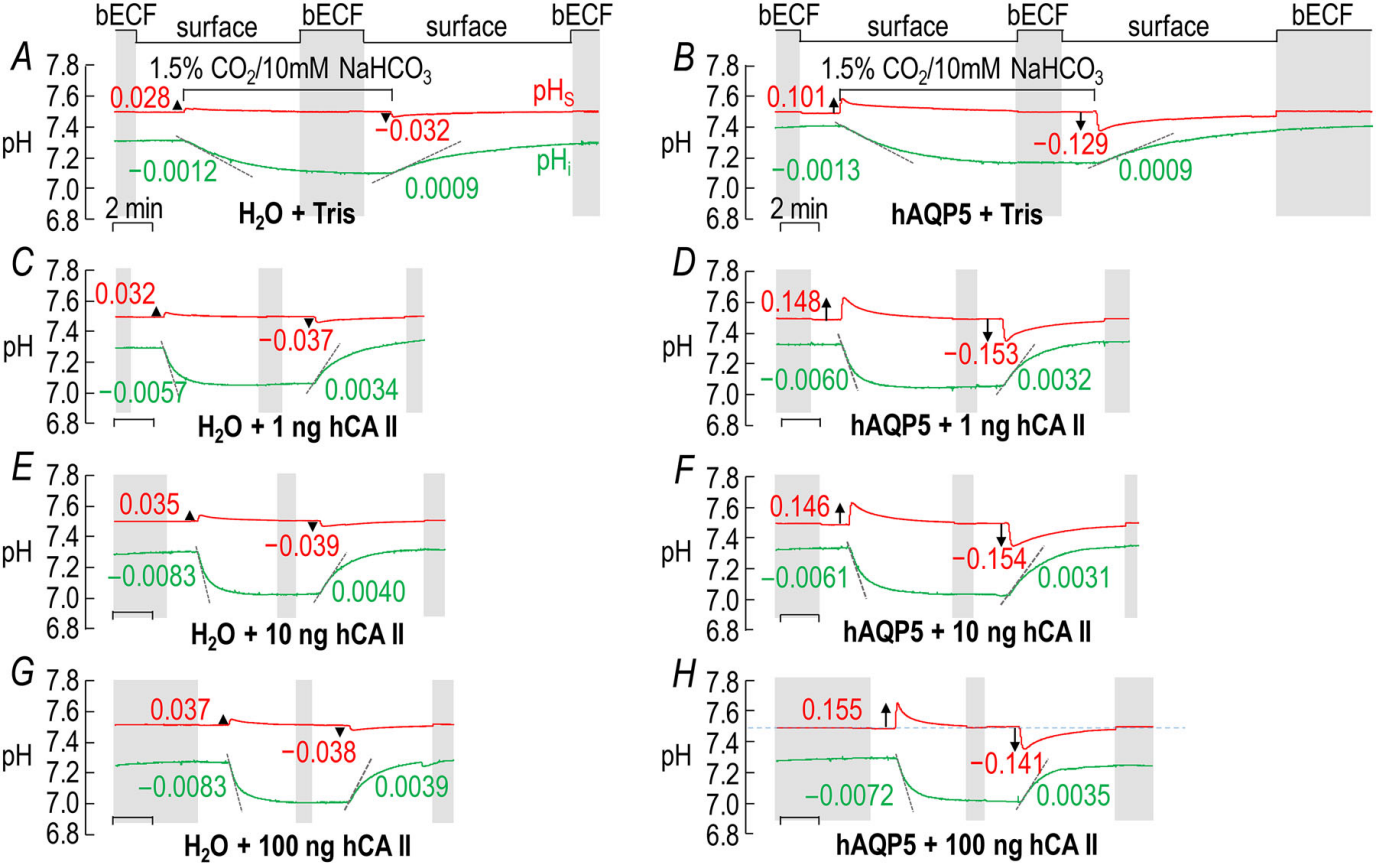
Representative pH_S_ and pH_i_ recordings: ±hAQP5, Δcytosolic hCA II, all in the absence of extracellular bCA (i.e. –bCA) A, ‘H_2_O + Tris’. We injected this oocyte with H_2_O (as a control for cRNA encoding hAQP5) on Day 1 and with ‘Tris’ (as a control for hCA II) on Day 4. B, ‘hAQP5 + Tris’. We injected this oocyte on Day 1, with cRNA encoding hAQP5, and then on Day 4 with ‘Tris’. *C*, ‘H_2_O + 1 ng hCA II’. Similar to panel A, except that on Day 4, we injected the oocyte with hCA II enzyme dissolved in ‘Tris’. *D*, ‘hAQP5 +1 ng hCA II’. Similar to panel B, except that on Day 4, we injected the oocyte with hCA II enzyme dissolved in ‘Tris’. *E*, ‘H_2_O + 10 ng hCA II’. *F*, ‘hAQP5 + 10 ng hCA II’. *G*, ‘H_2_O + 100 ng hCA II’. *H*, ‘hAQP5 + 100 ng hCA II’. The ‘Δ’ in the title and elsewhere in the paper implies that we choose among several levels of injected hCA II. The numbers in red are ΔpH_s_ and those in green are (dpH_i_/dt)_Max_ for the oocyte presented here. The stair‐step line at the top of *A* and *B* indicates the position of the pH_S_ electrode. The vertical shaded bars indicate times during which the pH_S_ electrode is in the bulk extracellular fluid (bECF) for recalibration; at other times the pH_S_ electrode dimples the cell surface for actual pH_S_ measurements. The colours of the rectangles that surround the panel labels (e.g. ‘H_2_O + Tris’) correspond to the colours of the bars in Fig. 3 and later figures.

The red record in Fig. 2*A* shows the pH_S_ data for a representative ‘H_2_O + Tris’ oocyte. The red record in Fig. 2*B* shows the comparable data for an ‘hAQP5 + Tris’ oocyte. Consistent with previous results (Geyer et al., 2013; Musa‐Aziz et al., 2009) the expression of hAQP5 leads to pH_S_ transients that are substantially greater in magnitude – both with the addition of CO_2_/HCO_3_^−^ (0.101 *vs*. 0.028) and with the removal of CO_2_/HCO_3_^−^ (–0.129 *vs*. –0.032) – than the mere injection of H_2_O into the oocytes (red records in Fig. 2*A* *vs*. *B*).

##### Summary

**Figure 3:**
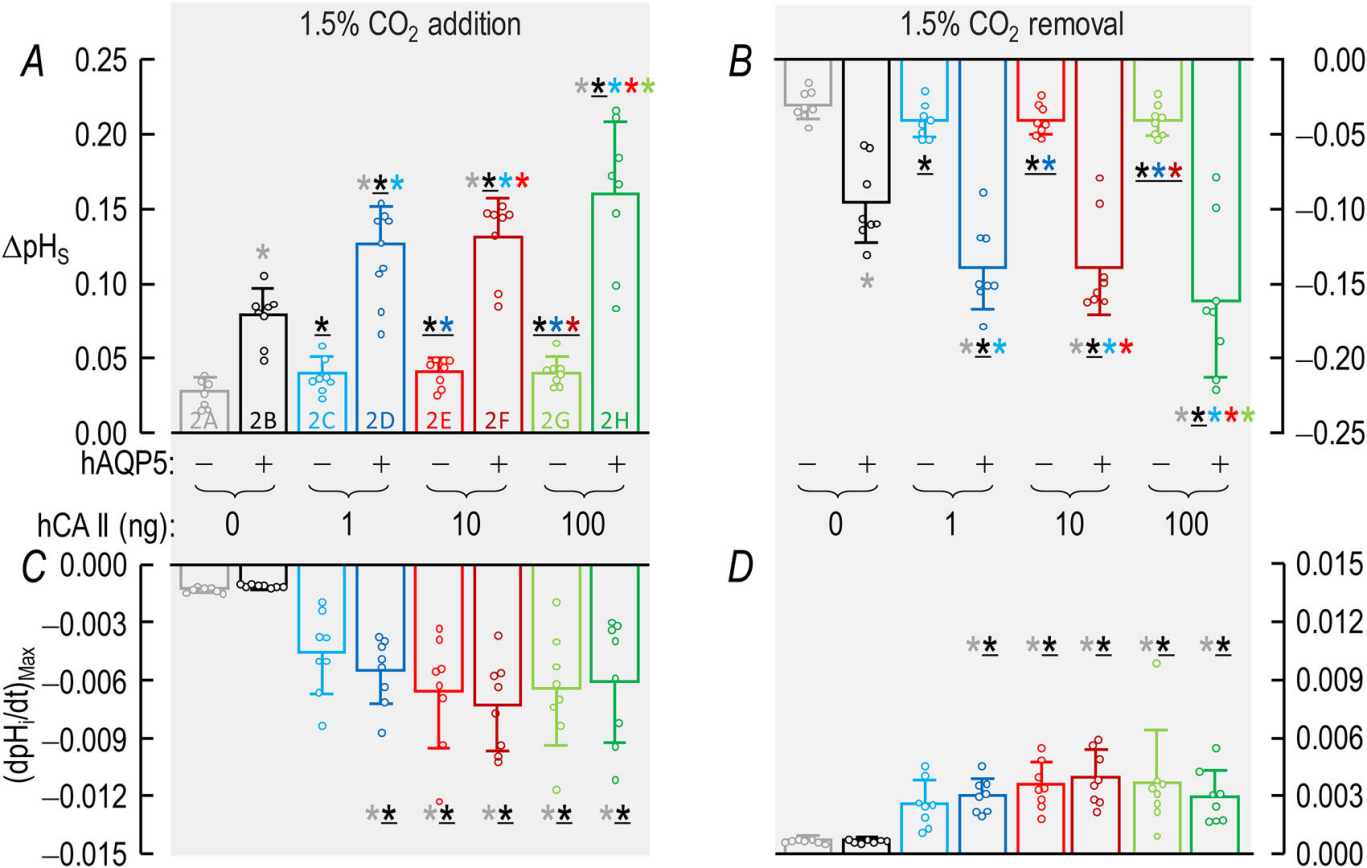
Summary of ∆pH_S_ and (dpH_i_/dt)_Max_ data from experiments like those in Fig. 2: ±hAQP5, Δcytosolic hCA II, all in the absence of extracellular bCA (i.e. –bCA) A, summary of ∆pH_S_ upon addition of 1.5% CO_2_/10 mM HCO_3_^−^. We computed individual ΔpH_S_ values as outlined in ‘Methods’ > ’Electrophysiological measurements’ > ‘Calculation of ΔpH_S_’. B, summary of ∆pH_S_ upon removal of 1.5% CO_2_/10 mM HCO_3_^−^. C, summary of (dpH_i_/dt)_Max_ upon addition of 1.5% CO_2_/10 mM HCO_3_^−^. We computed individual ΔpH_S_ values as outlined in ‘Methods’ > ’Electrophysiological measurements’ > ‘Calculation of “maximal” dpH_S_/dt’. D, summary of (dpH_i_/dt)_Max_ upon removal of 1.5% CO_2_/10 mM HCO_3_^−^. The ‘Δ’ in the title and elsewhere in the paper implies that we choose among several levels of injected hCA II. Data are presented as mean ± SD. ‘−’ and lighter‐coloured bars indicate that we injected oocytes with H_2_O; ‘+’ and darker‐coloured bars, with cRNA encoding hAQP5 on Day 1. ‘0’, ‘1’, ‘10’ and ‘100’ indicate the amount of hCA II (ng) injected into oocytes on Day 4. Grey/black and light‐/dark‐blue, red and green pairs of bars indicate eight groups of oocytes. These colours correspond to the colours of the rectangles that surround panel labels (e.g. ‘H_2_O + Tris’) in Fig. 2, A1. Statistical star/bar conventions: for each panel we used an ANOVA (see Methods^7^) to compare the data underlying each bar with that of every other bar in that panel (i.e. 7! total comparisons for each panel). The bars are meant to be read from right to left, starting with the darker of the two green bars. A coloured star above a bar indicates that the data underlying the light‐coloured data to the left differ significantly (see Statistics Table 3 for *P*‐values). Thus the light‐grey star above the dark‐green bar indicates that the dark‐green data differ significantly from the data represented by the light‐grey bar. The black star (underscored to emphasize that it is darker than grey) indicates that the dark‐green data also differ significantly from the data represented by the black bar. The light‐blue, light‐red and light green stars indicate statistically significant differences between the dark‐green data and those of the bars with the same colour as the stars. Similarly the light‐green data differ significantly from the black, dark‐blue and dark‐red data to the left. To reduce the number of displayed stars by half we do not use stars to indicate the reverse statistical significance (i.e. bars to the right). Thus even though the light‐grey bar has no stars its underlying data differ significantly from bars to its right with grey stars: the black, dark‐blue, dark‐red and dark‐green bars. The absence of a star in the right‐to‐left progression indicates a lack of statistical significance. Thus because the dark‐green bar lacks dark‐red and dark‐blue stars, the data underlying the dark‐green bar do not differ significantly from those underlying the dark‐red or dark‐blue bars. The complete statistical analyses are presented in Table A3A, A3B, A3C, A3D

For a larger number of these oocytes studied in the absence of both hCA II and bCA the bars in Fig. 3*A* provide the mean ΔpH_S_ data for CO_2_/HCO_3_^−^ addition in the absence (grey) and presence (black) of hAQP5, respectively. The grey and black bars in Fig. 3*B* summarize the corresponding data for CO_2_/HCO_3_^−^ removal.

##### Conclusions

From the statistical analysis of the data summarized in Fig. 3*A,B* we conclude that – in the absence of both hCA II and bCA both for CO_2_/HCO_3_^−^ addition and removal – expression of hAQP5 increases the magnitude of ΔpH_S_.

##### Interpretation

ΔpH_S_ (grey vs. black bars) in Fig. 3*A,B*; ±hAQP5, –hCA II, –bCA. Expression of hAQP5 increases $P_{M,CO_{2}}$ in eqn (1).

#### dpH_i_/dt

The green records in Fig. 2*A* and *B* show the pH_i_ data that correspond to the pH_S_ data presented above. The expression of hAQP5 does not produce a remarkable change in either the downward or upward (dpH_i_/dt)_Max_.

##### Summary

The grey and black bars in Fig. 3*C* show the mean downward (dpH_i_/dt)_Max_ for CO_2_/HCO_3_^−^ addition, ±hAQP5 – in the absence of both hCA II and bCA. The bars in Fig. 3*D* show the analogous data for CO_2_/HCO_3_^−^ removal.

##### Conclusions

With either CO_2_/HCO_3_^−^ addition or removal – in the absence of injected hCA II and extracellular bCA – the expression of hAQP5 does not significantly affect (dpH_i_/dt)_Max_.

##### Interpretation

(dpH_i_/dt)_Max_ (grey vs. black bars) in Fig. 3*C,D*; ±hAQP5, –hCA II, –bCA. (1) Expression of hAQP5, even though it markedly increases $P_{M,CO_{2}}$ as indicated by the ΔpH_S_ signals in Fig. 3*A,B*, does not increase the magnitude of (dpH_i_/dt)_Max_. The reason, in part, is that – without hCA II – the cytosolic CO_2_ hydration reaction (during CO_2_ influx) and dehydration reaction (during CO_2_ efflux) are rate‐limiting, thereby limiting the transmembrane CO_2_ gradient and choking the CO_2_ fluxes (see eqn (2)). In the second half of Results we will see that the availability of CO_2_ on the outer surface of the cell (replenished/consumed by bCA) also is rate‐limiting under the conditions of Fig. 2*A,B*. (2) Thus (dpH_i_/dt)_Max_ is a relatively insensitive indicator of CO_2_ fluxes in the absence of bCA in the bECF. And (3) a contributing factor may be the large diameter of oocytes (∼1.2 mm); a prolonged time for diffusion to/from the centre of the cell limits the dynamic range of (dpH_i_/dt)_Max_. Note that in the hands of Nakhoul et al. (1998) and Cooper and Boron (1998) AQP1 expression did not produce a statistically significant effect on (dpH_i_/dt)_Max_ under similar experimental conditions (i.e. in the absence of injected CA enzyme and with vitelline membrane intact).

### ±hAQP5, ΔhCA II, –bCA^8^: effects on ΔpH_S_ and (dpH_i_/dt)_Max_

To explore the interaction between hAQP5 and intracellular CA (CA_i_) in the diffusion of CO_2_ we injected oocytes on Day 1 with either ‘H_2_O’ or ‘H_2_O + cRNA encoding hAQP5’ and then, on Day 4, we injected oocytes with either ‘Tris’ as a control or ‘Tris + hCA II’ at hCA II levels of 1, 10 or 100 ng.

#### pH_s_

The red record in Fig. 2*C* shows the pH_S_ data for a representative ‘H_2_O + 1 ng hCA II’ oocyte and in Fig. 2*D* the comparable data for an ‘hAQP5 + 1 ng hCA II’ oocyte. Comparing the pH_S_ records between Fig. 2*C* *vs*. Fig. 2*A* we see that – in the absence of hAQP5 – the addition of 1 ng hCA II appears to increase the magnitude of the pH_S_ transient slightly. Musa‐Aziz et al. (2014a) had previously observed that hCA II – presumably with a higher specific activity – significantly increases the magnitude of ΔpH_S_ with both CO_2_/HCO_3_^−^ addition and removal. If we compare Fig. 2*D* *vs*. Fig. 2*B*, we see that – in the presence of hAQP5 – the injection of 1 ng hCA II has a much larger effect on the pH_S_ transient. Finally comparing Fig. 2*D* *vs*. Fig. 2*C* and Fig. 2*B* *vs*. Fig. 2*A* we see that the hAQP5 expression has a far greater effect on augmenting the magnitude of ΔpH_S_ – both with CO_2_/HCO_3_^−^ addition and removal – with the injection of 1 ng hCA II than with no added hCA II.

Note that in the above comparisons the pH_S_ electrode is ‘trans’ – or on the opposite side of the membrane – with respect to the hCA II that we added to the cytosol, a concept developed by Musa‐Aziz et al. (2014a, 2014b) and Occhipinti et al. (2014). In such cases one can conclude by intuition – but supported by mathematical simulations – that the trans‐side increase in ΔpH_S_ is indicative of an increased CO_2_ flux when hCA II is present in the cytosol. Thus based on Fig. 2*A–D* we can conclude that both the expression of hAQP5 alone (a large effect) and the injection of hCA II alone (a small effect), and especially the two in combination, increase CO_2_ fluxes into/out of oocytes.

Examining the effects of injecting larger amounts of hCA II we continue to see – in the absence of hAQP5 – very little effect on the pH_S_ transients (Fig. 2*E,G*) compared to the ‘Tris’ control (Fig. 2*A*). In the presence of hAQP5 the higher levels of injected hCA II (Fig. 2*F*,*H*) continue to enhance the pH_S_ transients greatly compared to the ‘AQP5 + Tris’ control (Fig. 2*B*), but the stimulatory effect is little more than with our 1 ng dose of hCA II (Fig. 2*D*).

In their earlier work Musa‐Aziz et al. (2014a) observed a baseline ΔpH_S_ (i.e. no injected CA) of slightly more than 0.04, whereas in the present study our baseline ΔpH_S_ is only ∼0.03. Thus the baseline values of CO_2_ permeability, surface CA activity or cytosolic CA activity in that previous study may have been somewhat greater than in the present study. Moreover Musa‐Aziz et al. (2014a) found that injecting 300 ng of recombinant hCA II approximately doubled the ΔpH_S_, whereas in the present study injecting 100 ng hCA II enzyme (a dose at which the ΔpH_S_ seemingly had already plateaued) increased ΔpH_S_ only by about one‐third (compare Fig. 2*G* *vs*. *A*). We presume that our commercially obtained hCA II, purified from red blood cells (RBCs), had a lower specific activity than the recombinant hCA II in the previous study. We abandoned attempts at injecting >100 ng/oocyte because these higher doses seemed to have deleterious effects on the oocytes, perhaps because of contaminants.

##### Summary

The lighter‐coloured blue, red and green bars in Fig. 3*A* display the mean ΔpH_S_ data for CO_2_/HCO_3_^−^ addition with increasing doses of hCA II, all in the absence of hAQP5 and bCA. The darker bars in Fig. 3*A* summarize comparable data but in the presence of hAQP5. For CO_2_/HCO_3_^−^ removal Fig. 3*B* reveals patterns that are similar to those in Fig. 3*A*.

##### Conclusions

For oocytes examined in the absence of bCA both for the addition and removal of CO_2_/HCO_3_^−^: (a)^9^ expressing hAQP5 (darker *vs*. lighter bars) increases the magnitude of ΔpH_S_ at every level of injected hCA II. (b) In the absence of hAQP5 (lighter bars) injecting oocytes with increasing amounts of hCA II, despite a modest upward trend, does not have a statistically significant effect on ΔpH_S_ magnitudes, neither for CO_2_/HCO_3_^−^ addition nor removal. (c) In the presence of hAQP5 (darker bars), injection of increasing amounts of hCA II tends to cause graded increases of ΔpH_S_ magnitudes, both for CO_2_ influx and efflux. Relative to no injected hCA II the effects reach statistical significance at all three levels of injected hCA II, although these three bars are not significantly different from each other.

##### Interpretation

ΔpH_S_ (colourful bars) in Fig. 3*A,B*; ±hAQP5, ΔhCA II, –bCA. (1) In the absence of hAQP5 (four lighter bars) $P_{M,CO_{2}}$ is the major rate‐limiting factor in both directions of CO_2_ diffusion and at all hCA II levels. Expression of hAQP5 augments $P_{M,CO_{2}}$ and thereby increases ΔpH_S_ (four darker bars). (2) During CO_2_/HCO_3_^−^ addition in control cells (–hAQP5, –hCA II; grey bars) disposal of incoming CO_2_ (in this case by cytosolic CA) is borderline rate‐limiting (i.e. with greater CA_i_ activity the flux would have been modestly greater), as noted previously by Musa‐Aziz et al. (2014a). Conversely during the subsequent CO_2_/HCO_3_^−^ removal replenishment of outgoing CO_2_ (here by hCA II), likewise, is borderline rate‐limiting. And (3) as we will see below in our presentation of data on bCA in the bECF for control cells (grey bars) the replenishment of CO_2_ on the extracellular surface is also rate‐limiting during CO_2_ influx. Conversely disposal of exiting CO_2_ at the cell surface during CO_2_ efflux is also rate‐limiting. (4) The greater effect of expressing hAQP5 in oocytes injected with 1 ng of hCA II (light‐ *vs*. dark‐blue bars) than in oocytes injected only with ‘Tris’ (grey *vs*. black bars) is an example of synergism.

#### dpH_i_/dt

The green records in Fig. 2*C,E,G* and *D,F,H* show the pH_i_ data that correspond to the pH_S_ data presented above, with incremental amounts of injected hCA II without hAQP5 (left side of Fig. 2) and with hAQP5 (right side). We observe that injecting even 1 ng hCA II into the cytosol produces a striking increase in (dpH_i_/dt)_Max_, both for CO_2_/HCO_3_^−^ application and removal. Increasing the injected hCA II to 10 ng further increases the downward (dpH_i_/dt)_Max_ but has only a modest effect on the upward (dpH_i_/dt)_Max_ in this example. Increasing injected hCA II to 100 ng has no additional effect. Comparing the left and right sides of Fig. 2 we see that hAQP5 expression seemingly has little effect on downward or upward (dpH_i_/dt)_Max_ regardless of the amount of injected hCA II.

Note that in the above (dpH_i_/dt)_Max_ analyses the pH_i_ electrode is ‘cis’ – or on the same side of the membrane – with respect to the hCA II that we added to the cytosol, again, a concept developed by Musa‐Aziz et al. (2014a, 2014b) and Occhipinti et al. (2014). In such cases one cannot use intuition to arrive at conclusions that the cis‐side acceleration of a pH_i_ change by hCA II is indicative of an increased CO_2_ flux. The reason is that – near the intracellular surface of the plasma membrane – the added hCA II greatly accelerates H^+^ formation during CO_2_ influx and H^+^ consumption during CO_2_ efflux. The pH_i_ electrode directly senses the changes in [H^+^], which only indirectly reflect CO_2_ fluxes. However previous mathematical simulations predict that the CO_2_ fluxes must have increased under these conditions (Musa‐Aziz et al., 2014a; Occhipinti et al., 2014).

##### Summary

The lighter/darker pairs of bars (–/+ hAQP5) in Fig. 3*C,D* précis the mean (dpH_i_/dt)_Max_ data for CO_2_/HCO_3_^−^ addition and removal.

##### Conclusions

Both for CO_2_/HCO_3_^−^ addition and removal: (a) expression of hAQP5 does not significantly affect (dpH_i_/dt)_Max_ at any level of injected hCA II (four sets of lighter *vs*. darker bars). And (b) in the absence of hAQP5 (four lighter bars) increasing amounts of injected hCA II cause (dpH_i_/dt)_Max_ values to trend towards greater magnitudes, although the effects do not reach statistical significance until 10 ng (light red) and 100 ng (light green).

##### Interpretation

(dpH_i_/dt)_Max_ (colourful bars) in Fig. 3*C,D*; ±hAQP5, ΔhCA II, –bCA. (1) Because the injected hCA II is ‘cis’ to the pH_i_ electrode, we cannot draw intuitive conclusions about CO_2_ fluxes as we compare the grey (dpH_i_/dt)_Max_ bar (i.e. –hAQP5) to the other three light‐coloured bars (i.e. increasing amounts of hCA II) or as we compare the black bar (i.e. +hAQP5) to the other three darker‐coloured bars. (2) Expression of hAQP5 (compare lighter *vs*. darker blue, red and green bars) does not increase (dpH_i_/dt)_Max_ magnitudes. As noted in our interpretation of ΔpH_S_ data immediately above^10^ this lack of effect probably reflects limited availability of CO_2_ at the extracellular surface during influx and disposal of CO_2_ at the extracellular surface during efflux. Such choking could be mitigated by introducing an extracellular carbonic anhydrase (CA_o_), as illustrated by the grey *vs*. black bars in Fig. 8*C*. As already mentioned^11^ a compounding factor may be the large diameter of the oocyte. (3) Nevertheless we know that expression of hAQP5 leads to a sizeable increase in $P_{M,CO_{2}}$ because of the parallel ΔpH_S_ data summarized by the lighter/darker pairs of bars in Fig. 3*A,B*.

#### V_m_

Figure *A*1 (in the Appendix) summarizes the end‐of‐experiment *V*_m_ data for the oocytes in Fig. 3. Note that oocytes expressing hAQP5 are modestly depolarized compared to the H_2_O‐injected control oocytes.

### ±hAQP5, ΔhCA II, –bCA: effects on pH_S_ relaxation

#### Theoretical considerations

Let us assume that the oocyte is a sphere, containing only an aqueous pH buffer, surrounded by a membrane permeable only to CO_2_ and initially devoid of CO_2_/HCO_3_^−^. If we now expose the oocyte to a CO_2_/HCO_3_^−^ solution, we could compute – given [CO_2_]_o_, initial pH_i_, buffering power and cell volume – the net number of CO_2_ molecules that would diffuse into the cell before the system came into equilibrium. The speed of this equilibration depends on the parameters that contribute to the description of Fick's law in eqns (1) and (2), including [CO_2_]_os_, [CO_2_]_is_ and $P_{M^{*},CO_{2}}$.

In their study of the impact of cytosolic CA II and CA IV (predominantly extracellular) on CO_2_ equilibration Musa‐Aziz et al. (2014a, 2014b) noted that, following the rapid upswing in pH_S_ triggered by CO_2_/HCO_3_^−^ application (or the rapid downswing in pH_S_ triggered by CO_2_/HCO_3_^−^ removal), (1) pH_S_ decayed following an approximately single‐exponential (SExp) time course, and that the time constant (τ) of this decay decreased markedly either with (2) injection of recombinant hCA II into the cytosol (see fig. 13 in Musa‐Aziz et al. (2014a) or (3) expression of hCA IV (see fig. 21 in Musa‐Aziz et al. (2014b). Moreover mathematical simulations based on a reaction‐diffusion model (Somersalo et al., 2012) corroborated nearly all of the essential observations (Occhipinti et al., 2014). In other words CA II and CA IV increased the speed (reflected by the rate constant = 1/τ) of CO_2_ equilibration, and thus 1/τ is an indirect measure of the transmembrane CO_2_ flux.

In the present study we note that the pH_S_ relaxations do not so nearly approximate an SExp decay as in the work by Musa‐Aziz et al. (2014a, 2014b), probably due to (1) minor differences in the tip of the pH_S_ electrodes, (2) the angles with which the pH_S_ electrode contacted the cell surface and (3) the precise location of the pH_S_ electrode on the oocyte surface (see Fig. 1 insets in Musa‐Aziz et al. (2014a). Rather we were able to obtain excellent fits of the present pH_S_‐decay data using a DExp function (i.e. one with two, not one, time constants). Note also that the aforementioned mathematical simulations suggest that the pH_S_ decays should not be perfectly exponential in the first place (Musa‐Aziz et al., 2014a, 2014b; Occhipinti et al., 2014). We regard the subtle differences in pH_S_‐decay waveforms between the previous and present work as the result of a divergence in technique that requires appropriate flexibility in data analysis. In the present study, where the pH_S_ decay is not a single exponential, we chose to use the maximal initial rate of pH_S_ relaxation as a surrogate for the single‐exponential 1/τ.

#### Exemplar data

**Figure 4:**
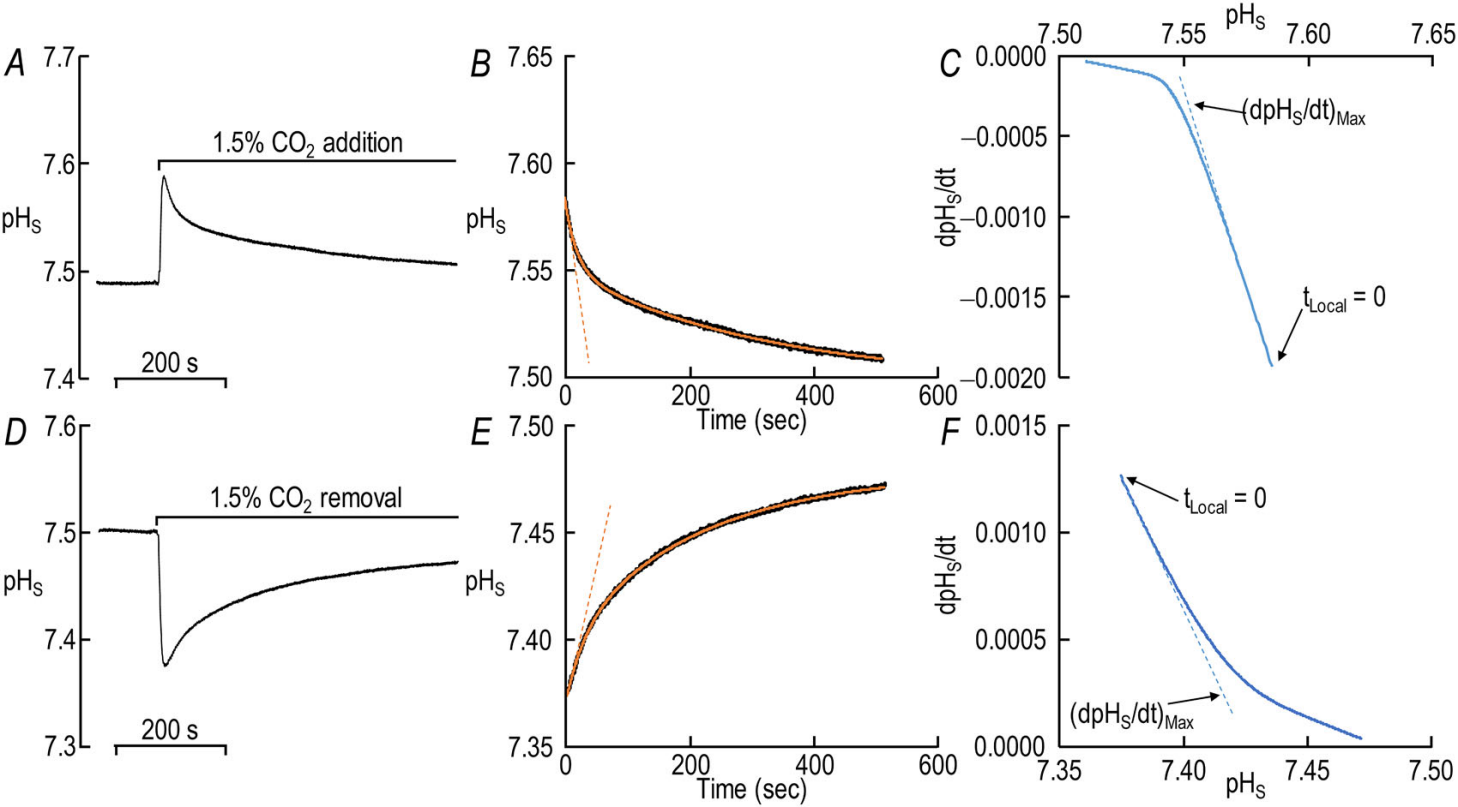
Analysis of (dpH_S_/dt)_Max_, the magnitude of maximal rate of relaxation of pH_S_ A, time course of surface pH (pH_S_) during addition of 1.5% CO_2_/10 mM HCO_3_^−^. This is a reproduction – with a magnified *y*‐axis – of the red record (during CO_2_ addition) in Fig. 2B. B, detail of panel A, showing only the pH_S_ relaxation, beginning from the time (*t*_Local_ = 0 s) of fastest pH_S_ descent. The jittery black record is the actual pH_S_ recording; the smooth orange curve is the result of a double‐exponential (DExp) curve fit. The dashed red line has a dpH_S_/dt‐*vs*.‐time slope that is the derivative of the fitted DExp function at *t*_Local_ = 0 s. C, dependence of dpH_S_/dt on pH_S_ during addition of CO_2_/HCO_3_^−^ application. We obtained dpH_S_/dt by computing the derivative of the DExp best‐fit function at the time that corresponds to the indicated pH_S_. Note that real time runs from right to left. The dashed blue line has a dpH_S_/dt‐*vs*.‐pH_S_ slope that corresponds to the value at *t*_Local_ = 0 s. A perfect single‐exponential fit of pH_S_ *vs*. time would have produced a straight line in this dpH_S_/dt‐*vs*.‐pH_S_ plot. Thus the break in the blue curve near pH_S_ = 7.54 is the demarcation between the dominance of a rapid/initial process and a slower/later process. D, time course of pH_S_ during removal of 1.5% CO_2_/10 mM HCO_3_^−^. This is a reproduction – with a magnified *y*‐axis – of the red record (during CO_2_ removal) in Fig. 2B. E, detail of panel D, processed as in panel B. F, dependence of dpH_S_/dt on pH_S_ during CO_2_/HCO_3_^−^ removal, processed as in panel C. Note that real time runs from left to right.

Figure 4*A* reproduces the pH_S_ time course for CO_2_/HCO_3_^−^ addition in Fig. 2*B* (i.e. ‘hAQP5 + Tris’). The jittery black record in Fig. 4*B* shows the time course of the actual pH_S_ relaxation, beginning at a local time (*t*_Local_) of 0, which we judged by eye to be the time of the fastest pH_S_ decay. The smooth orange curve is the result of a DExp curve fit. The dashed orange line is the tangent to the DExp function, evaluated at *t*_Local_ = 0. The blue curve in Fig. 4*C* is a plot of dpH_S_/dt, computed as the derivative of the best‐fit DExp function, *vs*. pH_S_. Here time flows from right to left. The break in slopes at pH_S_ ≅ 7.54 is the result of the second slower exponential process. The dashed blue line is the derivative of the DExp function, again calculated at *t*_Local_ = 0, but now plotted as a function of pH_S_. Although the choice of *t*_Local_ = 0 is subject to human error, the effect of misjudgement is likely to be minimal inasmuch as the DExp fit encompasses so many points, and because both the initial time course and the initial plot of dpH_S_/dt *vs*. pH_S_ are nearly linear.

Figure 4*D–F* is analogous to the top row of panels, except that here we analyse the CO_2_/HCO_3_^−^‐removal step in Fig. 2*B*. Note that all of the pH_S_ records are inverted, and that, in Fig. 4*F*, time runs from left to right.

##### Summary

**Figure 5:**
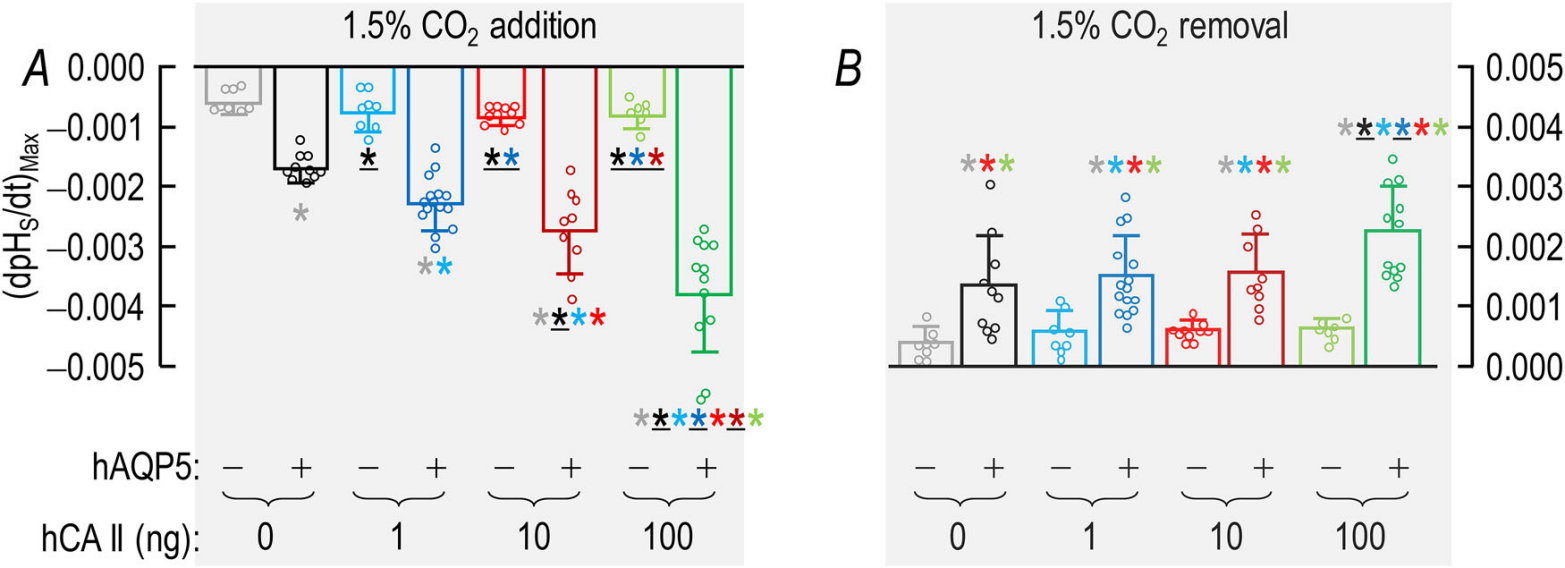
Summary of (dpH_S_/dt)_Max_ data from experiments like those in Fig. 2: ±hAQP5, Δcytosolic hCA II, all in the absence of extracellular bCA (i.e. –bCA) A, summary of (dpH_S_/dt)_Max_ upon addition of 1.5% CO_2_/10 mM HCO_3_^−^. We computed individual (dpH_S_/dt)_Max_ values as described in Fig. 4. B, summary of (dpH_S_/dt)_Max_ upon removal of 1.5% CO_2_/10 mM HCO_3_^−^. The ‘Δ’ in the title and elsewhere in the paper implies that we choose among several levels of injected hCA II. Data are presented as mean ± SD. ‘−’ and lighter‐coloured bars indicate that we injected oocytes with H_2_O; ‘+’ and darker‐coloured bars, with cRNA encoding hAQP5 on Day 1. ‘0’, ‘1’, ‘10’ and ‘100’ indicate the amount of hCA II (ng) injected into oocytes on Day 4. Grey/black and light‐/dark‐blue, red and green pairs of bars indicate eight groups of oocytes. These colours correspond to the colours of the rectangles that surround panel labels (e.g. ‘H_2_O + Tris’) in Fig. 2. Statistical star/bar conventions. See legend of Fig. 3 for a description of statistical significance. See Statistics Table 5 for *P*‐values. The complete statistical analyses are presented in Table A5A, A5B.

The layout of the synopses in Fig. 5*A,B* for (dpH_S_/dt)_Max_ data – CO_2_ addition/removal, ±hAQP5, with increasing amounts of injected hCA II – are the same as in Fig. 3*A* for the ΔpH_S_ data.

##### Conclusions

Both for CO_2_/HCO_3_^−^ addition and removal: (a) in the absence of hCA II expression of hAQP5 (grey *vs*. black bars) increases the magnitude of (dpH_S_/dt)_Max_, and the difference in mean values is statistically significant. The same is true for the expression of hAQP5 at each of the increasing hCA II levels (compare light *vs*. dark blue, red, and green bars). (b) In the absence of hAQP5, injecting hCA II in ever‐greater amounts (lighter bars) causes (dpH_S_/dt)_Max_ to trend upward but is without a statistically significant effect on (dpH_S_/dt)_Max_. And (c) in the presence of hAQP5, injecting increasing amounts of hCA II tends to produce ever‐greater magnitudes of (dpH_S_/dt)_Max_, with several of the differences reaching statistical significance, especially for CO_2_/HCO_3_^−^ addition. And (d) the effects on (dpH_S_/dt)_Max_ are in the same direction but statistically more robust than for ΔpH_S_ in Fig. 3*A,B*.

Interpretation: (dpH_S_/dt)_Max_ in Fig. 5*A,B*; ±hAQP5, ΔhCA II, –bCA. Our analysis of these data is similar to those for ΔpH_S_ in sections above.^12^

### ±hAQP5, ΔhCA II, –bCA: effects on other oocyte parameters

#### Initial pH_i_

**Figure 6:**
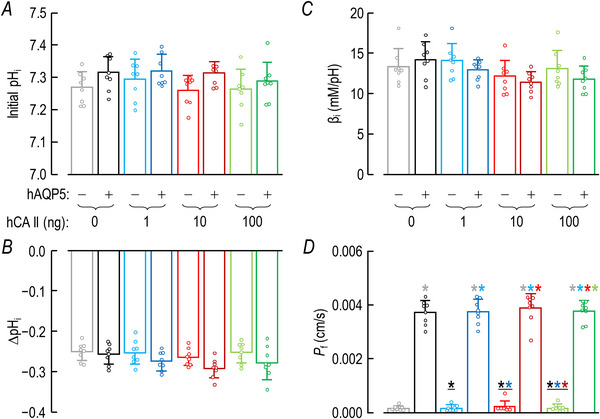
Summary of other oocyte parameters **from experiments like those in** **Fig**. **2**: ±hAQP5, Δcytosolic hCA II, all in the absence of extracellular bCA (i.e. –bCA) A, summary of initial pH_i_ values. We computed individual initial pH_i_, ΔpH_i_ (see panel B) and β_I_ (see panel C) values as outlined in ‘Methods’ > ’Electrophysiological measurements’ > ‘Calculation of intrinsic intracellular buffering power’. B, summary of ∆pH_i_ elicited by addition of 1.5% CO_2_/10 mM HCO_3_^−^. C, summary of intrinsic buffering power (β_I_). D, summary of *P*_f_. We computed individual initial pH_i_, ΔpH_i_ (see panel B) and β_I_ (see panel C) values as outlined in ‘Methods’ > ’Electrophysiological measurements’ > ‘Measurement of *P*_f_’. The ‘Δ’ in the title and elsewhere in the paper implies that we choose among several levels of injected hCA II. Data are presented as mean ± SD. ‘−’ and lighter‐coloured bars indicate that we injected oocytes injected with H_2_O; ‘+’ and darker‐coloured bars, with cRNA encoding hAQP5 on Day 1. ‘0’, ‘1’, ‘10’ and ‘100’ indicate the amount of hCA II (ng) injected into oocytes on Day 4. Grey/black and light‐/dark‐blue, red and green pairs of bars indicate eight groups of oocytes. These colours correspond to the colours of the rectangles that surround panel labels (e.g. ‘H_2_O + Tris’) in Fig. 2. Statistical star/bar conventions. See legend of Fig. 3 for a description of statistical significance. See Statistics Table 6 for *P*‐values. The complete statistical analyses are presented in Table A6A, A6B, A6C, A6D.

Figure 6*A* summarizes the initial pH_i_ values of oocytes subjected to the eight protocols of Fig. 2. All of the mean values are within 0.1 pH units of each other. Statistical analyses reveal no significant differences.

#### ΔpH_i_

Figure 6*B* summarizes the ΔpH_i_ values – that is, the decreases in pH_i_ – elicited by application of 1.5% CO_2_/NaHCO_3_ for the eight protocols. These values are all well within 0.1 of each other, and statistical analyses show no statistically significant differences among the eight ΔpH_i_ mean values.

#### β_I_

Figure 6*C* summarizes the intrinsic buffering power values of the eight groups of oocytes. Statistical analyses show no significant differences among the groups. The modest downward trend from the leftmost bars to the rightmost bars may reflect a faster equilibration of CO_2_ within oocytes with higher injected levels of hCA II, especially with hAQP5 expression. With a slower CO_2_ equilibration (see Fig. 2*A*) the measured ΔpH_i_ would tend to underestimate the value that we would have obtained at *t* =  ∞ and thus lead to an artificially elevated computed β_I_ value.

#### P_f_

After pH_S_ and pH_i_ were monitored we performed the *P*_f_ assay with each oocyte. Figure 6*D* summarizes our *P*_f_ data. The injection of cRNA encoding hAQP5 significantly increases *P*_f_ for each of the four groups of oocytes previously injected with no or increasing amounts of hCA II (compare lighter *vs*. darker bars in blue, red and green). Thus we can conclude that the oocytes express hAQP5 that traffics normally to the plasma membrane. Because the increase in *P*_f_ induced by hAQP5 was virtually the same for the four doses of hCA II, it is most likely that the CA has no effect on the monomeric pores that are responsible for the osmotic water permeability of hAQP5.

### ±hAQP5, –hCA II, +bCA: effects on ΔpH_S_ and (dpH_i_/dt)_Max_

In the final four sections of Results we examine oocytes studied in the presence of extracellular bCA and thereby explore the functional interaction between hAQP5 and CA_o_ in the transmembrane diffusion of CO_2_.

In this first of the final four sections we study oocytes in the absence of injected hCA II. On Day 1 we injected oocytes with either ‘H_2_O’ or ‘H_2_O + cRNA’ encoding ‘hAQP5’ and then on Day 4 we injected all oocytes with ‘Tris’. During the experiment we augment the extracellular solutions with 0.1 mg/ml of bCA, the same level as used previously by Musa‐Aziz et al. (2014b). This protocol is identical to that of Fig. 2*A,B* (–bCA) except for the presence of bCA.

#### pH_S_

**Figure 7:**
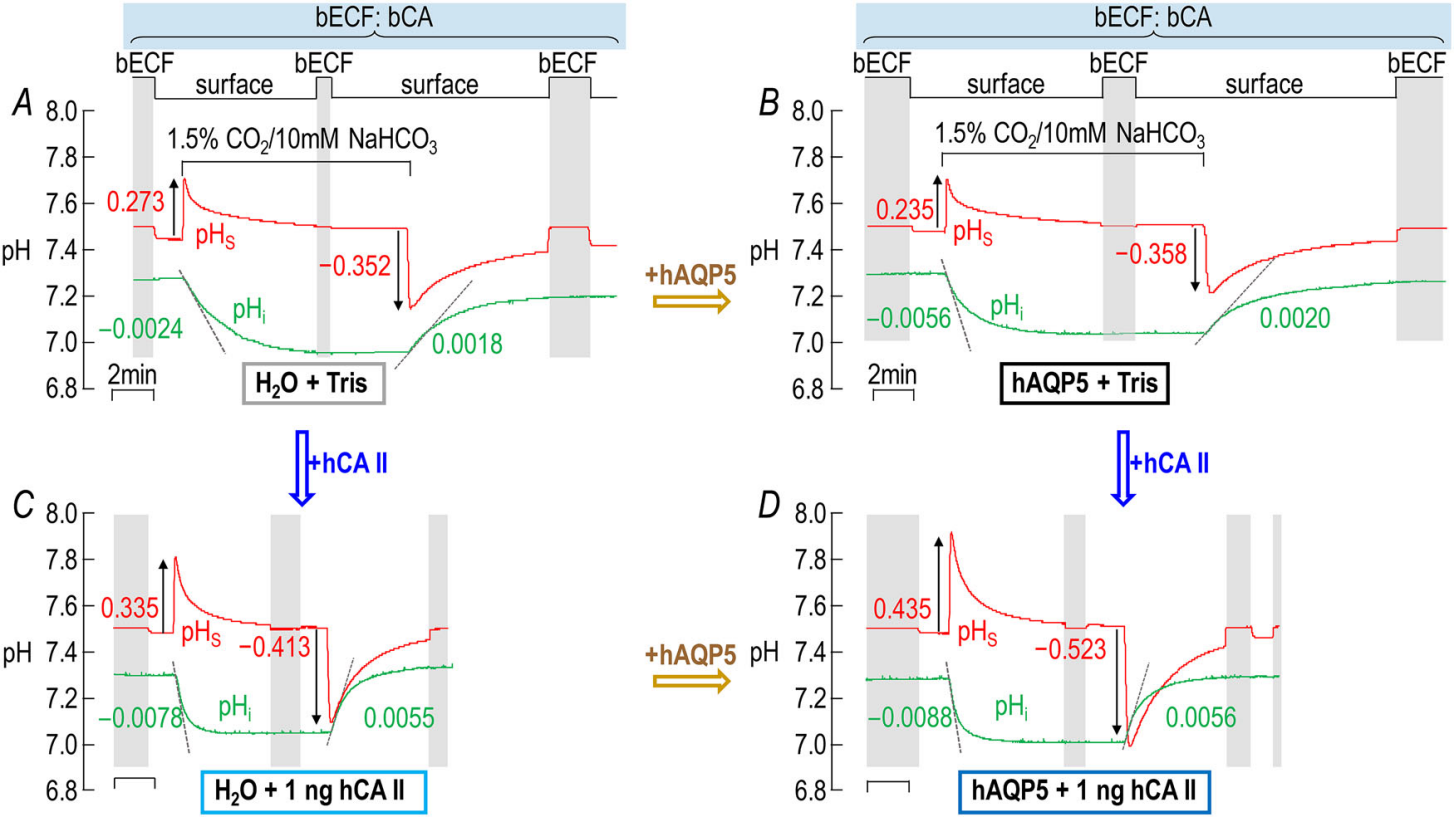
Representative pH_S_ and pH_i_ recordings: ±hAQP5, ±cytosolic hCA II, all in the presence of extracellular bCA (i.e. +bCA) A, ‘H_2_O + Tris’. We injected this oocyte with H_2_O (as a control for cRNA encoding hAQP5) on Day 1 and with ‘Tris’ (as a control for bCA II) on Day 4. B, ‘hAQP5 + Tris’. We injected this oocyte on Day 1 with cRNA encoding hAQP5 and then on Day 4 with ‘Tris’. *C*, H_2_O + 1 ng hCA II’. Similar to panel A, except that on Day 4, we injected the oocyte with hCA II enzyme dissolved in ‘Tris’. *D*, ‘hAQP5 + 1 ng hCA II’. Similar to panel B, except that on Day 4, we injected the oocyte with hCA II enzyme dissolved in ‘Tris’. The numbers in red are ΔpH_s_ and those in green are (dpH_i_/dt)_Max_ for the oocytes presented here. The stair‐step line at the top of *A* and *B* indicates the position of the pH_S_ electrode. The vertical shaded bars indicate times during which the pH_S_ electrode is in the bulk extracellular fluid (bECF) for recalibration; at other times the pH_S_ electrode dimples the cell surface for actual pH_S_ measurements. The colours of the rectangles that surround the panel labels (e.g. ‘H_2_O + Tris’) correspond to the colours of the bars in Fig. 8 and later figures. ECF, bCA indicates that we obtained all measurements while exposing oocytes to solutions containing 0.1 mg/ml of bCA.

  The red record in Fig. 7*A* shows the pH_S_ data for a representative ‘H_2_O + Tris’ oocyte and in Fig. 7*B* the comparable data for an ‘hAQP5 + Tris’ oocyte. Notice that the pH_S_ transients in Fig. 7*A* (+bCA, extracellular) are many fold larger – both with CO_2_/HCO_3_^−^ addition and removal – than their counterparts in Fig. 2*A* (–bCA). Musa‐Aziz et al. (2014b) had previously made a similar observation with extracellular hCA II (compare their figs. 13g and 15g).

##### Theoretical considerations

Note that in comparing pH_S_ transients in Fig. 7*A* *vs*. Fig. 2*A* the pH_S_ electrode is ‘cis’ to the added bCA in Fig. 7. A major reason that bCA increases the magnitude of ΔpH_S_ is that – near the extracellular surface of the plasma membrane – the added bCA greatly accelerates H^+^ consumption during CO_2_ influx (see Fig. 1*A*) and H^+^ production during CO_2_ efflux (see Fig. 1*B*). Although the pH_S_ electrode directly senses changes in [H^+^]_S_, these only indirectly reflect CO_2_ fluxes. Thus by comparing ±bCA (cis to the pH_S_ electrode) we can reach no intuitive conclusions about the possible effect of bCA on transmembrane CO_2_ fluxes from pH_S_ data. However previous mathematical simulations predict that the CO_2_ fluxes must have increased under these conditions (Musa‐Aziz et al., 2014b; Occhipinti et al., 2014), even though in some cases the effects may be too small to measure.

Exemplar data in Fig. 7*A* vs. Fig. 7*B*. The pH_S_ records show that in the presence of extracellular bCA the expression of hAQP5 does not produce an apparent increase in the magnitudes of the pH_S_ transients. This apparent lack of effect is quite different from the large fractional increases in ΔpH_S_ magnitudes that we observed in the absence of extracellular bCA (i.e. Fig. 2*B* *vs*. Fig. 2*A*), where the ΔpH_S_ magnitudes are so much smaller.

##### Summary

**Figure 8:**
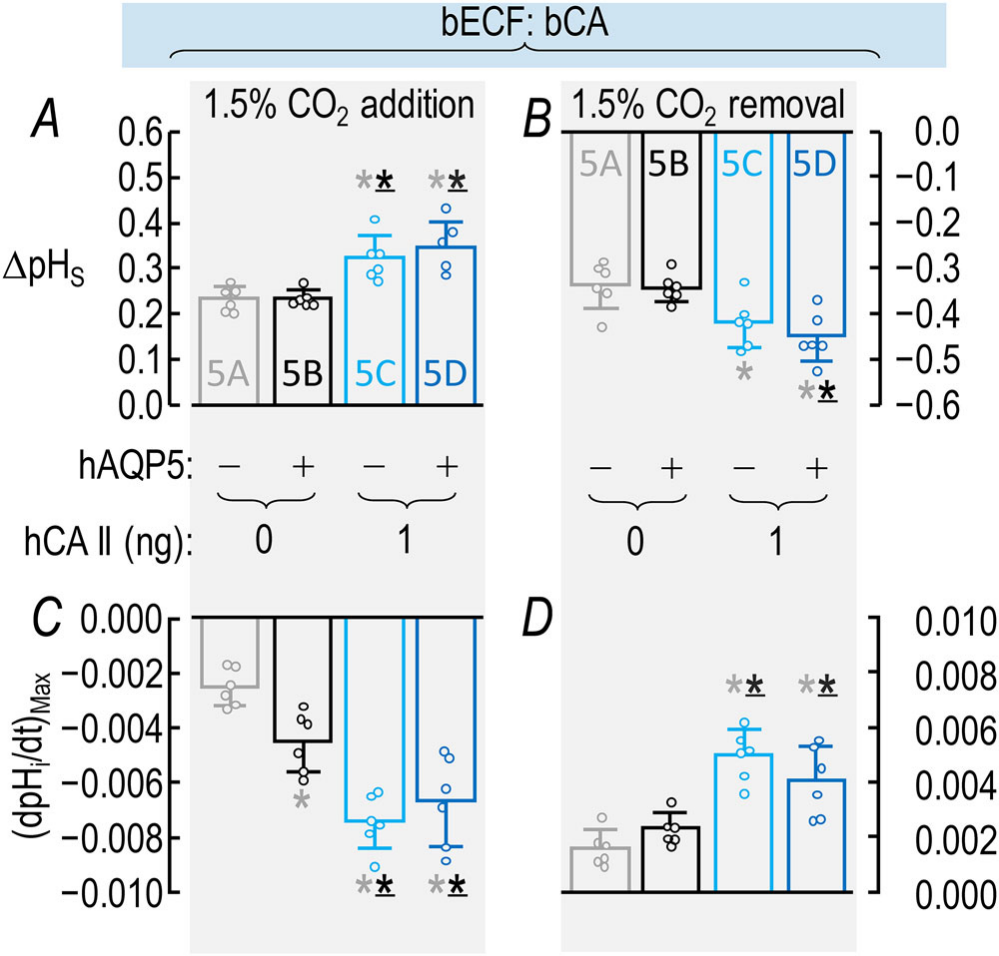
Summary of ΔpH_S_ and (dpH_i_/dt)_Max_ data from experiments like those in Fig. 7: ±hAQP5, ± **cytosolic** hCA II, all in the presence of extracellular bCA (i.e. +bCA) This figure is analogous to Fig. 3, which summarized ΔpH_S_ and (dpH_i_/dt)_Max_ data obtained from oocytes in the absence of bCA. A, summary of ∆pH_S_ upon addition of 1.5% CO_2_/10 mM HCO_3_^−^. We computed individual ΔpH_S_ values as outlined in ‘Methods’ > ’Electrophysiological measurements’ > ‘Calculation of ΔpH_S_’. B, summary of ∆pH_S_ upon removal of 1.5% CO_2_/10 mM HCO_3_^−^. C, summary of (dpH_i_/dt)_Max_ upon addition of 1.5% CO_2_/10 mM HCO_3_^−^. We computed individual ΔpH_S_ values as outlined in ‘Methods’ > ’Electrophysiological measurements’ > ‘Calculation of ‘maximal’ dpH_S_ /dt’. D, summary of (dpH_i_/dt)_Max_ upon removal of 1.5% CO_2_/10 mM HCO_3_^−^. Data are presented as mean ± SD. ‘−’ and lighter‐coloured bars indicate oocytes injected with H_2_O; ‘+’ and darker‐coloured bars indicate oocytes injected with cRNA encoding hAQP5 on Day 1. ‘0’ and ‘1’ indicate the amount of hCA II (ng) injected into oocytes on Day 4. Grey/black and light‐/dark‐blue pairs of bars indicate four groups of oocytes. These colours correspond to the colours of the rectangles that surround panel labels (e.g. ‘H_2_O + Tris’) in Fig. 7. Statistical star/bar conventions. See legend of Fig. 3 for a description of statistical significance. See Statistics Table 8 for *P*‐values. The complete statistical analyses are presented in Table A8A, A8B, A8C, A8D.

The grey and black bars in Fig. 8*A* show the mean ΔpH_S_ data for CO_2_/HCO_3_^−^ addition, ±hAQP5, all in the absence of hCA II but presence of extracellular bCA. Fig. 8*B* shows corresponding data for CO_2_/HCO_3_^−^ removal.

##### Conclusions

Considering oocytes examined in the presence of bCA: in the absence of hCA II expression of hAQP5 (black *vs*. grey bars) does not significantly affect ΔpH_S_, either for CO_2_/HCO_3_^−^ addition or removal.

##### Interpretation

ΔpH_S_ (grey vs. black bars) in Fig. 8*A,B*: ±hAQP5, –hCA  II, +bCA. (1) bCA promotes large transmembrane CO_2_ gradients. During CO_2_/HCO_3_^−^ addition bCA maintains relatively high CO_2_ levels near the extracellular face of the membrane and markedly increases CO_2_ influxes. During the subsequent CO_2_/HCO_3_^−^ removal bCA maintains relatively low CO_2_ levels at the cell surface. Thus even in the absence of hAQP5 (grey bars) the CO_2_ fluxes enabled by bCA are very high. (2) In parallel in a ‘cis‐side’ effect, bCA greatly increases the magnitudes of ΔpH_S_, as we can see by comparing Fig. 8*A*,*B* *vs*. Fig. 3*A,B*. Thus during CO_2_/HCO_3_^−^ addition bCA enhances H^+^ consumption, whereas during CO_2_/HCO_3_^−^ removal bCA enhances H^+^ production. (3) With expression of hAQP5 (black bars) – combining an increased $P_{M,CO_{2}}$ with the capacity of bCA to replenish/consume cell‐surface CO_2_ – cytosolic CA activity becomes rate‐limiting. Thus during CO_2_ addition CO_2_ rapidly builds up near the inner surface of the membrane, limiting the transmembrane gradient and choking CO_2_ influx – especially as assessed by a pH_S_ electrode that is ‘cis’ to the bCA. The same is true in reverse during CO_2_/HCO_3_^−^ removal. (4) The evidence that hAQP5 does indeed increase $P_{M,CO_{2}}$ will come below in our analysis of Fig. 8*C*.

#### dpH_i_/dt

The green records in Fig. 7*A* and Fig. 7*B* show the pH_i_ data collected simultaneously with the red pH_S_ transients (presented above) in these same panels. Note that (dpH_i_/dt)_Max_ values in Fig. 7*A* (+bCA) are substantially greater – both with CO_2_/HCO_3_^−^ addition and removal – than their counterparts in Fig. 2*A* (–bCA). Musa‐Aziz et al. (2014b) had previously made a similar observation with extracellular hCA II (compare their figures 13e and 15e). Note that in comparing pH_i_ changes in Fig. 7*A* *vs*. Fig. 2*A* the pH_i_ electrode is ‘trans’ to the bCA that we add to the bECF. During CO_2_/HCO_3_^−^ addition bCA accelerates extracellular CO_2_ formation and thus maintains a relatively high [CO_2_] near the extracellular surface of the membrane. Similarly during CO_2_/HCO_3_^−^ removal bCA accelerates CO_2_ consumption and thus maintains a relatively low [CO_2_] at the extracellular membrane surface. The greater transmembrane CO_2_ gradients lead to greater CO_2_ fluxes and thus faster ‘trans‐side’ pH_i_ changes. Thus comparing Fig. 7*A* *vs*. Fig. 2*A* we can conclude that bCA increases transmembrane CO_2_ fluxes.

If we now compare the pH_i_ records in Fig. 7*A* and Fig. 7*B*, we see that hAQP5 expression produces a substantial acceleration in the rate of pH_i_ decrease during CO_2_/HCO_3_^−^ addition, consistent with an hAQP5‐dependent increase in CO_2_ influx. During CO_2_/HCO_3_^−^ removal the presence of hAQP5 does not substantially speed the pH_i_ increase.

##### Summary

The grey and black bars in Fig. 8*C* summarize the downward (dpH_i_/dt)_Max_ for CO_2_/HCO_3_^−^ addition, ±hAQP5, all in the presence of extracellular bCA but absence of hCA II. Fig. 8*D* summarizes the corresponding data for CO_2_/HCO_3_^−^ removal.

##### Conclusions

Considering oocytes examined in the absence of hCA II but presence of bCA (Fig. 8*C,D*): during CO_2_/HCO_3_^−^ addition expression of hAQP5 (black *vs*. grey bars) significantly increases the magnitude of (dpH_i_/dt)_Max_. During CO_2_/HCO_3_^−^ removal expression of hAQP5 causes (dpH_i_/dt)_Max_ to trend faster, although the difference is not statistically significant.

##### Interpretation

(dpH_i_/dt)_Max_ (grey vs. black bars) in Fig. 8*C,D*; ±hAQP5, –hCA II, +bCA. See our ΔpH_S_ ‘Interpretation’ immediately above.^13^ (1) bCA promotes large transmembrane CO_2_ gradients. (2) In a ‘trans‐side’ effect bCA greatly increases the magnitudes of (dpH_i_/dt)_Max_, as we can see by comparing grey and black bars in Fig. 8*C,D* (+bCA) *vs*. Fig. 3*C,D* (–bCA) – consequences of the gradient effect in point #1. (3) Here in Fig. 8*C* (+bCA) hAQP5 increases the magnitude of (dpH_i_/dt)_Max_ during CO_2_/HCO_3_^−^ addition, demonstrating that the hAQP5 increases $P_{M,CO_{2}}$. In Fig. 3*C* (–bCA) the effect of expressing hAQP5 was nil because the limited availability of extracellular CO_2_ choked influx.

### ±hAQP5, +hCA II, +bCA: effects on ΔpH_S_ and (dpH_i_/dt)_Max_

To continue our exploration of the functional interaction of hAQP5 with CAs in the diffusion of CO_2_ in this next series of experiments we not only augment the extracellular solutions with 0.1 mg/ml bCA but also inject hCA II. On Day 1 we injected all oocytes with either ‘H_2_O’ or ‘H_2_O + cRNA’ encoding ‘hAQP5’ and then on Day 4 we injected all oocytes with 1 ng hCA II.

#### pH_S_

 The red records in Fig. 7*C* (–hAQP5, +hCA II, +bCA) and Fig. 7*D* (+hAQP5, +hCA II, +bCA) are analogous to those in panels A and B, except for the injection of hCA II. We now make three sets of comparisons:

- Comparing the red pH_S_ records between Fig. 7*C* (+hCA II) and Fig. 7*A* (–hCA II) – having in common –hAQP5, +bCA – we see that the addition of 1 ng hCA II increases the magnitudes of the pH_S_ transients – both with CO_2_/HCO_3_^−^ addition and removal. Recall that Musa‐Aziz et al. (2014a) made similar ΔpH_S_ observations ±cytosolic hCA II, though in the absence of added CA_o_. Because the pH_S_ electrode is ‘trans’ to the added hCA II (in comparing Fig. 7*C* *vs*. Fig. 7*A*), we can conclude that the hCA II has increased the transmembrane CO_2_ flux in the continued presence of extracellular bCA.
- Comparing the pH_S_ records in Fig. 7*D* (+hCA II) *vs*. Fig. 7*B* (–hCA II) – having in common +hAQP5, +bCA – we see that the addition of 1 ng hCA II again increases the magnitudes of the pH_S_ transients.
- Comparing Fig. 7*D* (+hAQP5) *vs*. Fig. 7*C* (–hAQP5) we see that on a background of injected hCA II and extracellular bCA hAQP5 expression has limited effect.
- Note that the pH_S_ transients here in Fig. 7*C,D* (±hAQP5, +hCA II, +bCA) are much larger than their counterparts in Fig. 2*C,D* (±hAQP5, +hCA II, –bCA).

##### Summary

(a) The grey *vs*. light‐blue bars in Fig. 8*A* show the mean ΔpH_S_ data for CO_2_/HCO_3_^−^ addition, ± hCA II, all in the absence of hAQP5 but presence of bCA. Fig. 8*B* shows the corresponding data for CO_2_/HCO_3_^−^ removal. (b) The black *vs*. dark‐blue bars compare ±hCA II but in the presence of both hAQP5 and bCA. (c) The light‐blue *vs*. dark‐blue bars compare ±hAQP5, all in the presence of both hCA II and bCA.

##### Conclusions

(a) The difference in mean values represented by the grey and light‐blue bars (±hCA II in the absence of hAQP5) is statistically significant, as is (b) the difference between the black and dark‐blue bars (±hCA II in the presence of hAQP5). Note that the effects in ‘a’ and ‘b’ correspond to the previously described synergistic effects of CA_i_ and CA_o_ (Musa‐Aziz et al., 2014a, 2014b; Occhipinti et al., 2014). (c) The difference between the light‐ *vs*. dark‐blue bars (±hAQP5 in the presence of hCA II and bCA) is not significant. (d) The statistical analysis summarized in Statistics Table 8E,F compares ΔpH_S_ amplitudes summarized in Fig. 8*A,B* (+bCA) *vs*. the grey/black/light‐blue/dark‐blue bars in Fig. 3*A,B* (–bCA). The result is that the effect of bCA is significant.

##### Interpretation

ΔpH_S_ (colourful bars) in Fig. 8*A,B*; ±hAQP5, +hCA II, +bCA. See ‘Interpretation’ for ΔpH_S_ in the previous section^14^: (1) the bCA enhances transmembrane CO_2_. (2) The ΔpH_S_ data summarized by the light/dark‐blue bars in Fig. 8*A,B* (+bCA) are in stark contrast to the analogous data in Fig. 3*A,B* (–bCA) – note that this is a ‘cis’ comparison (i.e. ±bCA→pH_S_) – where the ΔpH_S_ values are only about ⅛ to ½ as large during CO_2_/HCO_3_^−^ addition. (3) Expression of hAQP5 in the presence of 1 ng of injected hCA II (light‐ *vs*. dark‐blue bars), even with the increase in $P_{M,CO_{2}}$, does not increase ΔpH_S_ magnitudes because CO_2_ fluxes are choked by insufficient cytosolic CA activity (i.e. an insufficient transmembrane CO_2_ gradient). And (4) recall that hAQP5 does indeed increase $P_{M,CO_{2}}$ (see Fig. 8*C*). In addition to points #1 – #4, which are analogous to the corresponding points made in the previous section, (5) in a ‘trans‐side’ effect injection of 1 ng of hCA II – either in the absence of hAQP5 (grey *vs*. light‐blue bars) or in the presence of hAQP5 (black *vs*. dark‐blue bars) – increases the ΔpH_S_ magnitudes because the hCA II is able to increase transmembrane CO_2_ gradients sufficiently under these conditions.

#### dpH_i_/dt

  The green records in Fig. 7*C* and Fig. 7*D* show the pH_i_ data that correspond to the pH_S_ data presented above and lead to three comparisons:

- Comparing the pH_i_ records between Fig. 7*C* (+hCA II) and Fig. 7*A* (–hCA II) – both in the absence of hAQP5 – we see that the addition of 1 ng hCA II substantially increases the rates of pH_i_ changes, both with CO_2_/HCO_3_^−^ addition and removal. Musa‐Aziz et al. (2014a) made similar (dpH_i_/dt)_Max_ observations ±hCA II, though in the absence of added CA_o_.
- Comparing the pH_i_ records in Fig. 7*D* (+hCA II) *vs*. Fig. 7*B* (–hCA II) – now both in the presence of hAQP5 – we again see that the addition of 1 ng hCA II increases the magnitudes of (dpH_i_/dt)_Max_.
- Comparing the pH_i_ records in Fig. 7*D* (+hAQP5) *vs*. Fig. 7*C* (–hAQP5) we see that – on a background of injected hCA II and extracellular bCA – hAQP5 expression has little effect.
- Finally the magnitudes of (dpH_i_/dt)_Max_ in Fig. 7*C,D* are modestly larger than those in Fig. 2*C,D*.

##### Summary

The layout and meaning of the bars in Fig. 8*C,D* are the same as in Fig. 8*A,B*.

##### Conclusions

As we observed for the ΔpH_S_ data the differences between (a) the two lighter‐coloured bars (– hAQP5, ±hCA II) and (b) the two darker‐coloured bars (+hAQP5, ±hCA II) are statistically significant. In contrast, (c) the difference between the light‐ and dark‐blue bars (±hAQP5, + hCA II, +bCA) is not significant. (d) The statistical analysis summarized in Statistics Table 8G,H compares (dpH_i_/dt)_Max_ magnitudes from Fig. 8*C,D* (+bCA) *vs*. the grey/black/light‐blue/dark‐blue bars in Fig. 3*C,D* (–bCA). The result is that the effect of bCA is significant.

Interpretation: (dpH_i_/dt)_Max_ (colourful bars) in Fig. 8*C,D*; ±hAQP5, +hCA II, +bCA. See ‘Interpretation’ for ΔpH_S_ immediately above^15^: (1) bCA magnifies transmembrane CO_2_ gradients. (2) In a ‘trans’ effect the magnitudes of the light‐ and dark‐blue bars in Fig. 8*C,D* (+hCA II, +bCA) are modestly larger than their counterparts in Fig. 3*C,D* (+hCA II, –bCA) – a specific example of enhanced transmembrane CO_2_ gradients noted in point #1. (3) The light‐ and dark‐blue bars (i.e. ±hAQP5, +hCA II, +bCA) are not significantly different, presumably because 1 ng of injected hCA II does not raise cytosolic CA activity sufficiently to prevent choking the dominant effects of hAQP5 (↑$P_{M,CO_{2}}$) and bCA (↑CO_2_ gradient). We predict that greater CA_i_ activities (e.g. 100 ng) would have alleviated the choke and revealed a much taller +hAQP5 bar. And (4) hAQP5 does indeed increase $P_{M,CO_{2}}$ as evidenced by the comparison of grey *vs*. black bars in Fig. 8*C*. In addition to points #1–#4 (analogous to those made in previous section) (5) we note that – because of a ‘cis’ effect – we cannot intuitively interpret the effects of injecting hCA II on (dpH_i_/dt)_Max_, either in the absence of hAQP5 (grey *vs*. light‐blue bars) or in the presence of hAQP5 (black *vs*. dark‐blue bars). Even though we cannot intuitively assess ±hCA II effects from (dpH_i_/dt)_Max_ we know from the corresponding ΔpH_S_ data in Fig. 8*A,B* that the injection of hCA II – by enhancing transmembrane CO_2_ gradients – must have increased the CO_2_ fluxes.

### ±hAQP5, ±hCA II, +bCA: effects on pH_S_ relaxation

#### Summary

**Figure 9:**
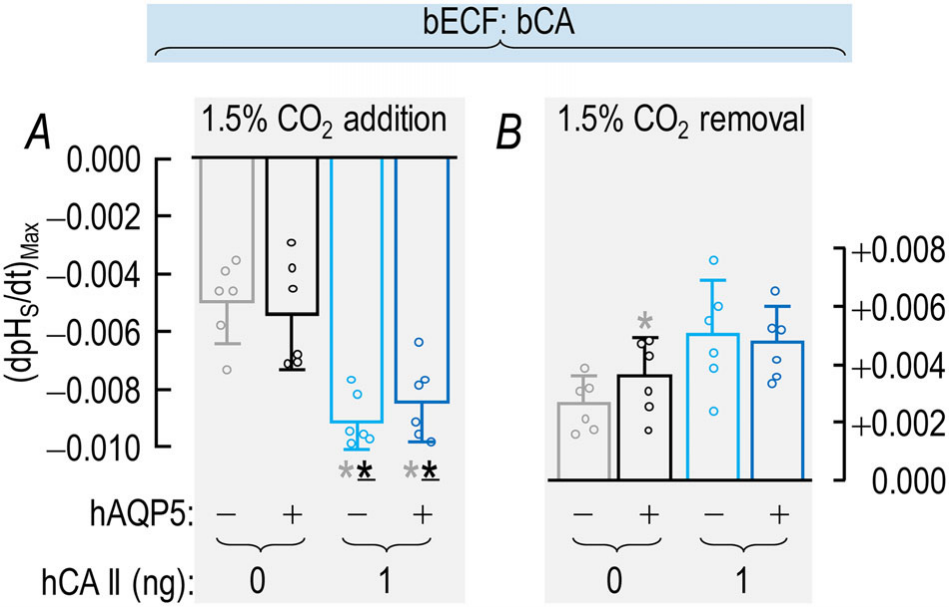
Summary of (dpH_S_/dt)_Max_ data from experiments like those in **Fig**. **8**: ±hAQP5, ±cytosolic hCA II, all in the presence of extracellular bCA (i.e. +bCA) This figure is analogous to Fig. 5, which summarized (dpH_S_/dt)_Max_ data obtained from oocytes in the absence of bCA. A, summary of (dpH_S_/dt)_Max_ upon addition of 1.5% CO_2_/10 mM HCO_3_^−^. We computed individual (dpH_S_/dt)_Max_ values as described in Fig. 4. B, summary of (dpH_S_/dt)_Max_ upon removal of 1.5% CO_2_/10 mM HCO_3_^−^. Data are presented as mean ± SD. ‘−’ and lighter‐coloured bars indicate oocytes injected with H_2_O; ‘+’ and darker‐coloured bars indicate oocytes injected with cRNA encoding hAQP5 on Day 1. ‘0’ and ‘1’ indicate the amount of hCA II (ng) injected into oocytes on Day 4. Grey/black and light‐/dark‐blue pairs of bars indicate four groups of oocytes. These colours correspond to the colours of the rectangles that surround panel labels (e.g. ‘H_2_O + Tris’) in Fig. 7. Statistical star/bar conventions. See legend of Fig. 3 for a description of statistical significance. See Statistics Table 9 for *P*‐values.The complete statistical analyses are presented in Table A9A, A9B.

The layout of Fig. 9*A,B* is similar to that of Fig. 8*A,B* (i.e. ΔpH_S_), except that here in Fig. 9*A,B* we examine (dpH_S_/dt)_Max_ as we did in Fig. 5*A,B*.

#### Conclusions

For oocytes examined in the presence of bCA:

(a) In the absence of hAQP5 injecting hCA II (grey *vs*. light‐blue bars in Fig. 9*A*) produces an increase in (dpH_S_/dt)_Max_ during CO_2_/HCO_3_^−^ addition; the difference is statistically significant. During CO_2_/HCO_3_^−^ removal the results trend towards a greater magnitude but do not reach statistical significance (Statistics Table 9*B*). These results in Fig. 9*A* (–hAQP5, ±hCA II, +bCA) are in contrast to those in Fig. 5*A* (–hAQP5, ±hCA II, –bCA), where injected hCA II is without statistically significant effect, and even the trends were mild.

**Statistics Table 9:** For clarity within the figure panel we present tables of *P*‐values for one‐way ANOVA with Tukey's *post hoc* means comparison for the data presented in Fig. 9. For all tables α is 0.05, and significant *P*‐values are highlighted in bold. A, *P*‐values for means comparisons of (dpH_S_/dt)_Max_ on CO_2_ addition. B, *P*‐values for means comparisons of (dpH_S_/dt)_Max_ on CO_2_ removal. C, *P‐*values for means comparisons of β_i_. D, *P*‐values for means comparisons of *P*_f_ data

*9A (dpH_S_/dt)_Max_ on CO_2_ addition*				
cRNA		H_2_O	hAQP5	H_2_O
	hCA II (ng)	‘Tris’	‘Tris’	1
hAQP5	‘Tris’	0.960		
H_2_O	1	**3.38×10^−4^**	**0.00103**	
hAQP5	1	**0.00221**	**0.00674**	0.842

*9B (dpH_S_/dt)_Max_ on CO_2_ removal*				
cRNA		H_2_O	hAQP5	H_2_O
	hCA II (ng)	‘Tris’	‘Tris’	1
hAQP5	‘Tris’	**0.0288**		
H_2_O	1	0.625	0.281	
hAQP5	1	0.0604	0.984	0.461

And (b) in oocytes expressing hAQP5 injecting hCA II (black *vs*. dark blue bars) increases the magnitude of (dpH_S_/dt)_Max_ during CO_2_/HCO_3_^−^ addition with a statistically significant difference. This treatment produces a slight upward trend during CO_2_/HCO_3_^−^ removal. Note that these increments (due to injecting hCA II) are no greater than for oocytes not expressing hAQP5. Viewed differently expressing hAQP5 significantly increases the magnitude of (dpH_S_/dt)_Max_ in only 1 of 4 cases (CO_2_/HCO_3_^−^ removal in the absence of hCA II, grey *vs*. black bars in Fig. 9*B*). These results contrast to those in Fig. 5 (– bCA) where – in the presence of hAQP5 – increasingly large hCA II injections tended to produce graded increases (dpH_S_/dt)_Max_ and for both CO_2_/HCO_3_^−^ addition and removal. We note, however, that the hCA II injections in Fig. 9 were either 0 or 1 ng, whereas in the Fig. 5 (–bCA) study those were as high as 100 ng.

(c) Expression of hAQP5 in the absence of hCA II (grey *vs*. black bars in Fig. 9*A,B*) or in the presence of hCA II (light‐ *vs*. dark‐blue bars) has no effect on (dpH_S_/dt)_Max_ except during CO_2_/HCO_3_^−^ removal in the absence of hCA II (grey *vs*. black bars, Fig. 9*B*). These results contrast with those of Fig. 5 (– bCA), where hAQP5 expression increases (dpH_S_/dt)_Max_ magnitudes robustly and consistently.

(d) The (dpH_S_/dt)_Max_ magnitudes in Fig. 9 are ∼2‐ to ∼3‐fold greater than the analogous ones in Fig. 5. The differences are greatest in the absence of hCA II, where the lighter‐coloured bars in Fig. 9 are ∼10‐fold greater.

Interpretation: (dpH_S_/dt)_Max_ in Fig. 9*A,B*; ±hAQP5, ±hCA II, +bCA. Our analysis of these (dpH_S_/dt)_Max_ is similar to that for the ΔpH_S_ data in the previous section.^16^

#### ±hAQP5, ±hCA II, +bCA: effects on other oocyte parameters

**Figure 10:**
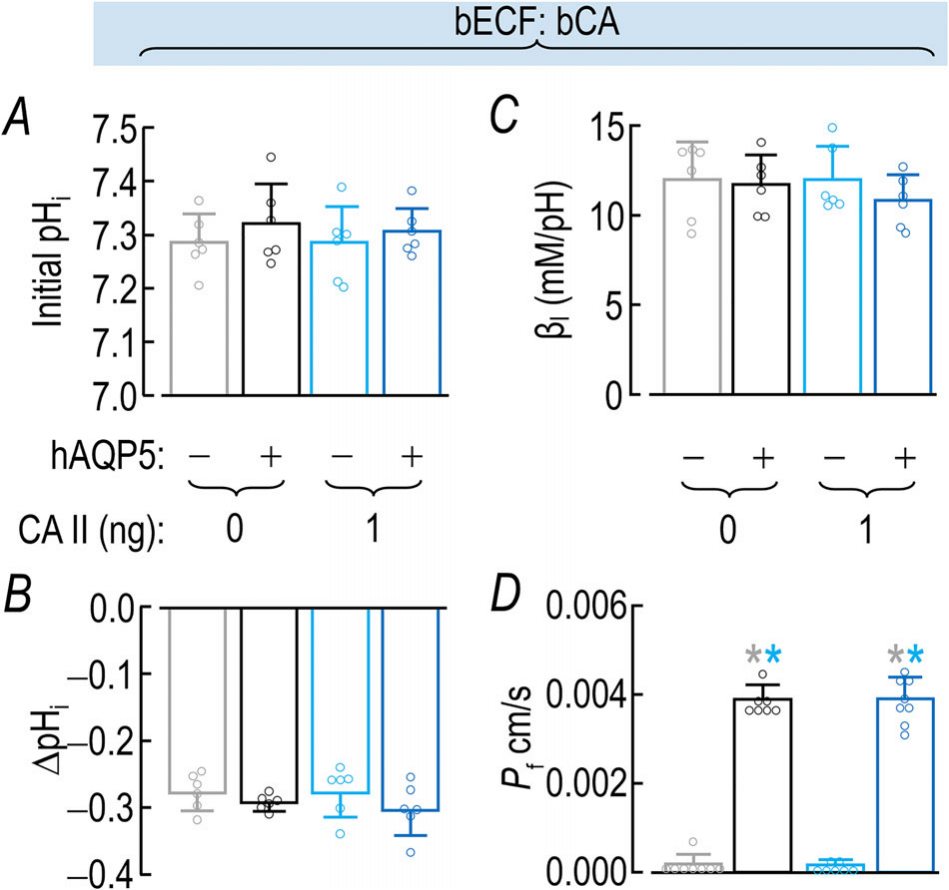
Summary of other oocyte parameters from experiments like those in Fig. 7: ±hAQP5, ±cytosolic hCA II, all in the presence of extracellular bCA (i.e. +bCA) This figure is analogous to Fig. 6, which summarized comparable data obtained from oocytes in the absence of bCA. A, summary of initial pH_i_ values. We computed individual initial pH_i_, ΔpH_i_ (see panel B) and β_I_ (see panel C) values as outlined in ‘Methods’ > ‘Electrophysiological measurements’ > ‘Calculation of intrinsic intracellular buffering power’. B, summary of ∆pH_i_ elicited by addition of 1.5% CO_2_/10 mM HCO_3_^−^. C, summary of intrinsic buffering power (β_I_). D, summary of *P*_f_. We computed individual initial pH_i_, ΔpH_i_ (see panel B) and β_I_ (see panel C) values as outlined in ‘Methods’ > ’Electrophysiological measurements’ > ‘Measurement of *P*_f_’. Data are presented as mean ± SD. ‘−’ and lighter‐coloured bars indicate oocytes injected with H_2_O; ‘+’ and darker‐coloured bars indicate oocytes injected with cRNA encoding hAQP5 on Day 1. ‘0’ and ‘1’ indicate the amount of hCA II (ng) injected into oocytes on Day 4. Grey/black and light‐/dark‐blue pairs of bars indicate eight groups of oocytes. These colours correspond to the colours of the rectangles that surround panel labels (e.g. ‘H_2_O + Tris’) in Fig. 7. Statistical star/bar conventions. See legend of Fig. 3 for a description of statistical significance. See Statistics Table 10 for *P*‐values. The complete statistical analyses are presented in Table A10A, A10B, A10C, A10D.

The four bars in each of the panels of Fig. 10 are analogous to the four leftmost bars of the four panels in Fig. 6; the difference is that in Fig. 10 we summarize experiments in which we exposed oocytes to 0.1 mg/ml extracellular bCA.

##### Initial pHi

  Statistical analyses, as in the case of Fig. 6*A*, reveal no significant differences among the four mean initial pH_i_ values in Fig. 10*A*. Moreover Statistics Table 10E, which summarizes an analysis of values in Fig. 10*A* (+bCA) *vs*. the grey/black/light‐blue/dark‐blue bars in Fig. 6*A* (–bCA), reveals no significant effect of adding bCA.

**Statistics Table 10:** For clarity within the figure panel we present tables of *P*‐values for one‐way ANOVA with Tukey's *post hoc* means comparison for the data presented in Fig. 10. For all tables α is 0.05, and significant *P*‐values are highlighted in bold. A, *P*‐values for means comparisons of initial pH_i_ data. B, *P*‐values for means comparisons of ∆pH_i_ data. C, *P‐*values for means comparisons of β_I_. D, *P*‐values for means comparisons of *P*_f_ data. Panels E–H provide descriptive statistics and means comparisons (see Methods, Statistical analysis) for comparisons of data represented by the grey, black, light‐blue and dark‐blue bars in Fig. 6 (–bCA) *vs*. Fig. 10 (+bCA). E, Initial pH_i_. F, ΔpH_i_ amplitude on addition of CO_2_/HCO_3_^−^. G, β_I_. H, *P*_f_.

*10A initial pH_i_*							
cRNA		H_2_O	hAQP5	H_2_O			
	hCA II (ng)	‘Tris’	‘Tris’	1			
hAQP5	‘Tris’	0.752					
H_2_O	1	1.00	0.727				
hAQP5	1	0.939	0.974	0.925			

*10B ∆pH_i_ data*							
cRNA		H_2_O	hAQP5	H_2_O			
	hCA II (ng)	‘Tris’	‘Tris’	1			
hAQP5	‘Tris’	0.798					
H_2_O	1	1.00	0.779				
hAQP5	1	0.486	0.949	0.465			

*10C Buffering power (β_I_)*							
cRNA		H_2_O	hAQP5	H_2_O			
	hCA II (ng)	‘Tris’	‘Tris’	1			
hAQP5	‘Tris’	0.994					
H_2_O	1	1.00	0.994				
hAQP5	1	0.652	0.799	0.659			

*10D P*_f_ *data*							
cRNA		H_2_O	hAQP5	H_2_O			
	hCA II (ng)	‘Tris’	‘Tris’	1			
hAQP5	‘Tris’	**2.10×10^−12^**					
H_2_O	1	0.999	**2.10×10^−12^**				
hAQP5	1	**2.10×10^−12^**	0.999	**2.10×10^−12^**			

*10E, initial pH_i_ values ±bCA in bECF (*Fig. *6A* *vs*. Fig. *10A*)							
Descriptive stats.	*n*	Mean	SD	SEM			
bECF	32	7.30	0.0551	0.00975			
bECF + bCA	24	7.31	0.0603	0.0123			
**Means comparison**	**Mean diff**.	**SEM**	***Q*‐value**	***P*‐value**	**α**	**LCL**	**UCL**
bECF *vs*. bECF + bCA	0.00669	0.0155	0.610	0.668	0.0500	−0.0244	0.0378

*10F, ∆pH_i_ upon CO_2_ addition ±bCA in bECF (*Fig. *6B* *vs*. Fig. *10B*)							
Descriptive stats.	*n*	Mean	SD	SEM			
bECF	32	−0.258	0.0258	0.00456			
bECF + bCA	24	−0.285	0.0308	0.00628			
**Means comparison**	**Mean diff**.	**SEM**	***Q*‐value**	***P*‐value**	**α**	**LCL**	**UCL**
bECF *vs*. bECF + bCA	−0.0270	0.00756	5.04	**7.74×10^−4^**	0.0500	0.0118	0.0421

*10G, β*_i_ *values for oocytes ±bCA in bECF (*Fig. *6C* *vs*. Fig. *10C*)							
Descriptive stats.	*n*	Mean	SD	SEM			
bECF	32	13.6	1.93	0.341			
bECF + bCA	24	11.6	1.73	0.353			
**Means comparison**	**Mean diff**.	**SEM**	***Q*‐value**	***P*‐value**	**α**	**LCL**	**UCL**
bECF *vs*. bECF + bCA	−2.03	0.498	5.75	**1.55×10^−4^**	0.0500	−3.02	−1.03

*10H, P_f_ ±bCA in bECF (*Fig. *6D* *vs*. Fig. *10D*)							
Descriptive stats.	*n*	Mean	SD	SEM			
bECF	32	0.00196	0.00184	3.25×10^−4^			
bECF + bCA	30	0.00204	0.00192	3.50×10^−4^			
**Means comparison**	**Mean diff**.	**SEM**	***Q*‐value**	***P*‐value**	**α**	**LCL**	**UCL**
bECF *vs*. bECF + bCA	8.03×10^−5^	4.77×10^−4^	0.238	0.867	0.0500	−8.74×10^−4^	0.00103

#### ΔpH_i_

  We observe no significant differences among mean ΔpH_i_ values in Fig. 10*B*, as observed among groups in Fig. 6*B*. However Statistics Table 10F, which summarizes an analysis of values in Fig. 10*B* (+bCA) *vs*. the grey/black/light‐blue/dark‐blue bars in Fig. 6*B* (–bCA), reveals a small but significant difference due to the addition of bCA. The magnitudes of ΔpH_i_ values in Fig. 6*B* are somewhat smaller, presumably reflecting the slower transmembrane equilibration of CO_2_ due to the absence of bCA, particularly at lower levels of injected hCA II.

#### β_I_

Continuing the trend from the previous two panels we observe no significant differences among mean β_I_ in Fig. 10*C*, as described among analogous conditions in Fig. 6*C*. However Statistics Table 10G, which summarizes an analysis of values in Fig. 10*C* (+bCA) *vs*. the grey/black/light‐blue/dark‐blue bars in Fig. 6*C* (–bCA), reveals a small but significant difference – similar to the analysis of the ΔpH_i_ data above. Because their ΔpH_i_ magnitudes tend to be smaller, the β_I_ values in Fig. 6*C* (– bCA) are somewhat larger than those in Fig. 10*C* (+bCA).

#### P_f_

As for the previous three panels the mean values summarized in Fig. 10*D* (+bCA) – now for *P*_f_ – are indistinguishable from the comparable values in Fig. 6*D* (–bCA). Our statistical analyses in Statistics Table 10H reveal no significant effect of bCA on *P*_f_. Thus we can conclude that extracellular bCA – a mixture of CA I and CA II – does not interfere with the monomeric pores of hAQP5. Moreover as we did in our analysis of Fig. 6*D* we can conclude, from a comparison of the black and dark‐blue bars, that the hCA II does not functionally interfere with the monomeric pores.

## Discussion

### Historical context

#### Permeability *vs*. gradient

Although previous papers on CO_2_ diffusion across membranes have addressed the role of channels or the role of CAs, the present paper is the first to undertake a systematic examination of both channels and CAs, as well as their synergistic interaction to enhance CO_2_ fluxes. The underlying principle is Fick's law of diffusion, which we reproduce from eqn (2):

(3)

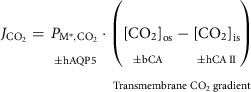

and embellish to emphasize that the key parameters in the present study increase $P_{M^{*},CO_{2}}$ (i.e. hAQP5) and the transmembrane CO_2_ gradient. During CO_2_ influx bCA (when present) in the extracellular fluid maintains a relatively high [CO_2_]_os_, whereas hCA II (when present) maintains a relatively low [CO_2_]_is_, the result being an enhanced inwardly directed CO_2_ gradient. Conversely during CO_2_ efflux bCA in the extracellular fluid maintains a relatively low [CO_2_]_os_, whereas hCA II maintains a relatively high [CO_2_]_is_, the result being an enhanced outwardly directed CO_2_ gradient.

It is perhaps worth noting that similar principles are at work during O_2_ fluxes across the erythrocyte membrane. In that case, according to papers not yet peer reviewed (Moss et al., 2025; Occhipinti et al., 2025; Zhao et al., 2025), AQP1, the Rh complex and as‐yet‐unidentified channel(s) make the dominant contribution to $P_{M,CO_{2}}$. Moreover it is haemoglobin that maximizes transmembrane O_2_ gradients (ignoring haemoglobin diffusion within the cytoplasm) by serving as a sink or source of O_2_ near the plasma membrane. In the laboratory experiments on O_2_ efflux from RBCs one can use an extracellular O_2_ scavenger like sodium dithionite to consume O_2_ near the extracellular face of the membrane in an action that is analogous to that of bCA in the present experiments on CO_2_ efflux.

Working on artificial lipid bilayers Gutknecht et al. (1977) showed that mobile (more or less freely diffusible) CA enhances CO_2_ fluxes (measured using ^14^C‐labeled CO_2_) but only in the presence of sufficient non‐CO_2_/HCO_3_^−^ buffers (phosphate, HEPES, ‘Tris’). Their interpretation is that the CA increases the transmembrane CO_2_ gradient by replenishing CO_2_ on one side of the membrane and consuming it on the other. This replenishment (or consumption) requires that HCO_3_^−^ diffuse through the unconvected layers on opposite sides of the membrane to serve as either the source or product of the CA reactions. Moreover the buffers act as either a source of or sink for H^+^ in these reactions.

The trio of papers by Musa‐Aziz et al. (2014a, 2014b) and Occhipinti et al. (2014) extended this line of reasoning to *Xenopus* oocytes, which they injected with recombinant hCA II (to an apparent concentration ∼50% higher than in RBCs) and/or cRNA encoding hCA IV (which contributes to both extracellular‐surface and cytosolic CA activity). In addition they systematically varied [CO_2_]_o_ and [HEPES]_o_ and performed three‐dimensional reaction‐diffusion mathematical modelling to enable a quantitative interpretation of the data. As anticipated from the earlier artificial lipid‐bilayer work they found that each CA alone augmented CO_2_ diffusion by increasing transmembrane CO_2_ gradients. However the combination of the two is not simply additive but highly synergistic. The reason is that, with a CA on just one side of the membrane, net CO_2_ fluxes are limited by the build‐up or depletion of CO_2_ on the other – the ‘choking’ (or throttling) effect to which we allude in the Results section of the present paper. They also demonstrated an additional synergism involving an extracellular non‐HCO_3_^−^ buffer (i.e. HEPES), assessed by ‘trans‐side’ measurements of pH_i_, and examined the impact of increasing [CO_2_]_o_ during CO_2_/HCO_3_^−^ application. In their papers they did not address the issue of CO_2_ permeability.

The first studies to address the $P_{M,CO_{2}}$ term in eqn (3) were those by Nakhoul et al. (1998) and Cooper and Boron (1998), who showed that AQP1 – in addition to being a H_2_O channel – is an effective CO_2_ channel. Musa‐Aziz et al. (2007) and Geyer et al. (2013) later examined a range of mammalian AQPs and showed that AQP5 has the highest CO_2_/H_2_O permeability ratio. The only study to broach channel‐CA interactions was that by Nakhoul et al. (1998), who studied CO_2_‐induced pH_i_ changes in oocytes with the vitelline membrane intact. They found that the stimulatory effect of AQP1 on CO_2_ influx into oocytes occurs only with bCA injected into the cytoplasm. In the present paper the analogous experiment is summarized by the light‐ *vs*. dark‐blue bars in Fig. 3*C*, where hAQP5 expression causes (dpH_i_/dt)_Max_ to trend faster. Perhaps a difference, as suggested by others (Vilas et al., 2015), is that CA II binds to AQP1 but not AQP5.

The present paper builds on the work of Musa‐Aziz and Occhipinti (Musa‐Aziz et al., 2014a, 2014b; Occhipinti et al., 2014) and generally uses identical approaches, with three important differences:

First rather than recombinant hCA II in the present study we use hCA II purified commercially from RBCs.

Second rather than heterologously expressing hCA IV we add bCA to the extracellular fluid. The reasons for this switch are fourfold: (1) hCA IV expression in oocytes increases not only cell‐surface CA activity but also cytosolic CA activity. The oocyte membrane confines bCA to the outside of the cell. (2) The co‐expression of hCA IV and hAQP5 adds an extra burden of heterologous expression and also introduces potential competition between the injected cRNAs encoding the two proteins (‘ribosome steal’). (3) We also chose to add purified CA protein to the bECF because in principle we know the precise increase in CA_o_ activity, which is difficult to know in the case of protein expression. And (4) we chose bCA because it is a less‐expensive combination of bCA I (less active) and bCA II (more active) *vs*. hCA II; this is an important practical consideration during continuous‐flow experiments, which consume considerable volumes of experimental solutions.

Third rather than working only with oocytes having a background $P_{M,CO_{2}}$ we alternated between injections of H_2_O and cRNA encoding hAQP5.

#### Buffering

One might ask whether the introduction of a CA increases buffering power (β). The short answer is ‘no’. We divided β for CO_2_/HCO_3_^−^ into two components: (1) CO_2_/HCO_3_^−^ buffering power and (2) non‐CO_2_/HCO_3_^−^ buffering power (see Thornell et al., 2025). In the present study we see that increasing [CO_2_]_o_ causes pH_i_ to fall – an example of intracellular ‘respiratory acidosis’. The CO_2_/HCO_3_^−^ buffer system does contribute to the CO_2_‐induced acid load, and thus CAs have no impact on β, even though they may markedly increase the rate at which pH_i_ reaches a new steady state (see Figs. 6*C* and 10*C*). It is the non‐CO_2_/HCO_3_^−^ buffers that limit the extent of a CO_2_‐induced pH change. If the intracellular acid load had been ‘metabolic’ in nature (e.g. the iontophoretic injection of KHCO_3_ or HCl as pioneered by the late Roger Thomas in 1976), then both CO_2_/HCO_3_^−^ and non‐CO_2_/HCO_3_^−^ buffers would have contributed to β_Total_. However even here CAs would only have speeded the attainment of CO_2_/HCO_3_^−^ equilibration and not increased the magnitude of buffering.

### pH measurements made ‘cis’ *vs*. ‘inter’ *vs*. ‘trans’ to the altered parameter

The papers of Musa‐Aziz et al. (2014a, 2014b) and Occhipinti et al. (2014) introduced the concept of pH measurements, used as an indirect measure of something else (e.g. CO_2_ flux), being made ‘cis’ or ‘trans’ to a compartment with an alteration that directly affects pH (e.g. CA activity, non‐HCO_3_^−^ buffering power). Thus if one wishes to assess the effects on CO_2_ flux of injecting (or not injecting) hCA II into the cytosol, the measurement of pH_i_ – ‘cis’ to the added hCA II – does not provide intuitive insight into the effects of hCA II’ on transmembrane CO_2_ fluxes. Of course at the same time as making the pH_i_ measurement one may also monitor pH_S_ – ‘trans’ to the added hCA II in the same cell. Interpreting the data from such a ‘trans’ perspective does provide intuitive insight into CO_2_ fluxes. The same principles apply in the opposite sense if one wishes to assess the effects of adding (or not adding) bCA to the bECF. The measurement of pH_S_ – ‘cis’ to the added bCA – does not provide intuitive insight into the effects of bCA on CO_2_ fluxes, whereas the measurement of pH_i_ – ‘trans’ – does.

The reason for the ‘cis‐side’ prohibition is that the enzymatic activity of the CA produces or consumes H^+^ and thus can produce large pH changes that are independent of – and could conflated with the interpretation of – changes in transmembrane CO_2_ fluxes. Thus intuitive interpretations can be extremely difficult, even though mathematical modelling can unravel alterations in ‘cis‐side’ pH from alterations in the flux of CO_2_ or other buffer components. Such unraveling was a major component of the studies of Musa‐Aziz et al. (2014a, 2014b) and Occhipinti et al. (2014), which demonstrated that both cytosolic and extracellular CA increase transmembrane CO_2_ gradients and thus increase CO_2_ fluxes. Of course avoiding a prohibited ‘cis‐side’ interpretation requires proper experimental design. However the ‘cis‐side’ prohibition is more an issue of data interpretation: when altering any parameter that affects acid–base reaction rates in Fig. 1*A*, an intuitive interpretation requires that one examine pH ‘trans’ to the alteration.

Stated simply ‘trans‐side’ pH measurements are valuable for intuitive interpretations because trans‐side pH change can occur only as the result of altered CO_2_ fluxes across the membrane.

The experiments summarized by Fig. 8 are a useful case study. bCA is present continuously in the bECF. Thus when we inject hCA II, the pH_S_ measurements – ‘trans’ to the hCA II – do provide intuitive insight into the effect of hCA II on transmembrane CO_2_ fluxes because in this particular comparison the bCA status is unchanging. Basing one's intuition on pH_i_ measurements – ‘cis’ to the added hCA II – would be unwise. However if we compare Fig. 8 (+bCA) with Fig. 3 (–bCA), an intuitive assessment of bCA effects must come from pH_i_ measurements – ‘trans’ to the ±bCA condition. Thus ‘cis’ *vs*. ‘trans’ is not a matter of data *per se* but a matter of perspective during data analysis.

Finally in Figs. 3 and 8 we also assess the effects of expressing the integral membrane protein hAQP5. Both pH_S_ and pH_i_ measurements are neither ‘cis’ nor ‘trans’ to the altered expression of hAQP5 and thus the altered $P_{M,CO_{2}}$. One might use the term ‘inter’ (Latin, ‘within’, ‘inside’ or ‘between’) to indicate the position of the protein relative to the membrane. In such an ‘inter’ situation one could use both pH_S_ and pH_i_ measurements to assess the data intuitively.

### Molecular mechanism of CO_2_ permeability

Given the suggestion that AQPs may conduct dissolved gases via the hydrophobic central pore of the tetramer (Boron, 2010; Wang et al., 2007) it is not surprising that molecular dynamics simulations are consistent with the hypothesis that O_2_ (Zhang & Chen, 2013) and CO_2_ (Alishahi & Kamali, 2019) can diffuse through the central pore of hAQP5. Preliminary data on hAQP5 suggest that (1) mutating amino acid residues near the outer mouth of the central pore to residues with bulky side chains (e.g. T41F) or (2) creating a divalent‐cation binding site (T41H) and then adding Ni^2+^ or Zn^2+^ greatly reduces ΔpH_S_, as supported by crystal structures and molecular dynamics (Shinn et al., 2024). In addition a preliminary report suggests that with hAQP1 the mercurial pCMBS can block one component of $P_{M,CO_{2}}$, the stilbene derivative DIDS can block an equally large component, and together the two can eliminate the CO_2_ permeability of hAQP1 (Musa‐Aziz et al., 2025). Thus the emerging picture is that some CO_2_ can permeate the four hydrophilic monomeric pores (at least of hAQP1), whereas another component – presumably the major component of CO_2_ – moves through the central pore of hAQP5.

### Diagnostic power of ΔΔΔpH_S_ ± hCA II in identifying enhanced CO_2_ permeability

**Figure 11:**
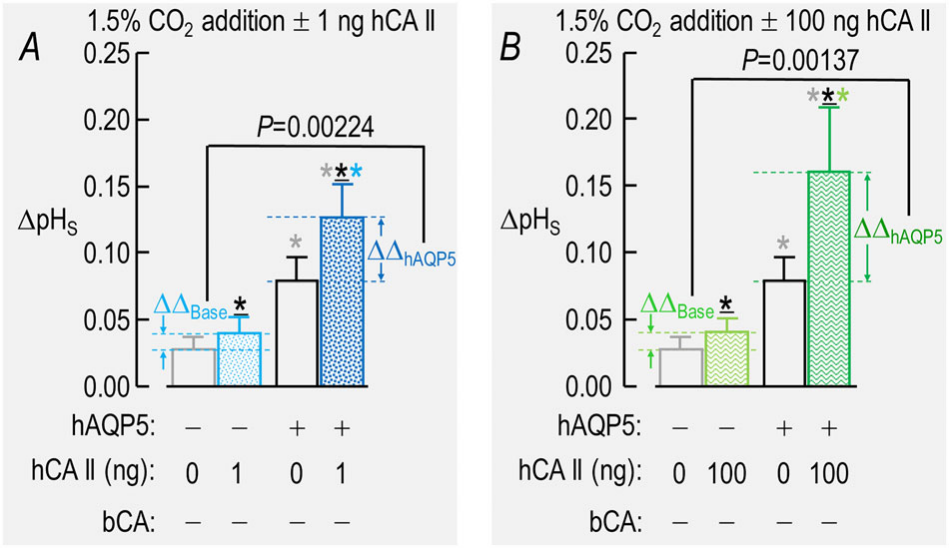
Effect of expressing hAQP5 on ΔΔpH_S_: comparison of selected ΔpH_S_ bars extracted from Fig. 3*A* (–bCA) Here we rearrange bars from Fig. 3A to juxtapose two bars representing the same hAQP5 status (‘–’, absent; ‘+’, heterologously expressed): the left bar in a pair representing the absence of hCA II and the right bar representing the presence of injected hCA II. A, ΔΔpH_S_ determined with ±1 ng of injected hCA II during addition of CO_2_/HCO_3_^−^. B, ΔΔpH_S_ determined with ±100 ng of injected hCA II during addition of CO_2_/HCO_3_^−^. For a pair of bars the difference between ΔpH_S_ values is ΔΔpH_S_. Thus ΔΔ_Base_ is difference between ΔpH_S_ values under baseline conditions (i.e. no hAQP5) induced by the addition of the trans‐side CA (i.e. CA II); ΔΔ_hAQP5_ is the corresponding difference between ΔpH_S_ values in AQP5‐expressing oocytes. Comparing two pairs of bars the difference between ΔΔ_hAQP5_ and ΔΔ_Base_ values is ΔΔΔpH_S,hAQP5_. Although no bCA is present in any of these experiments, we include bCA status (‘–’, absent from bECF) to facilitate comparisons with Fig. 12. Statistical star/bar conventions. See legend of Fig. 3 for a description of statistical significance. The stars, which indicate statistical significance among the individual bars, have the same meanings as in Fig. 3A. The *P*‐values indicate statistical significance between the respective ΔΔ_Base_ and ΔΔ_hAQP5_ bar pairs and reflect the effect of adding 1 ng hCA II (panel *A*) or 100 ng hCA II (Panel *B*) to the ‘trans’ side of the membrane during the pH_S_ measurement. See Table A11*A* and Table A11*B* for the statistics summary (including all *P*‐values reported by the stars).

In Fig. 11 we rearrange CO_2_‐influx bars from Fig. 3*A* so that we can easily compare the effects – on ΔpH_S_ – of adding a CA, namely hCA II, on the side of the membrane ‘trans’ to the pH_S_ measurement. The pair of bars on the left of Fig. 11*A* shows that in the absence of hAQP5 injecting 1 ng of hCA II into oocytes has only a minor effect on ΔpH_S_. That is the baseline ΔΔpH_S_ – or ΔΔpH_S,Base_ – is small. However in the presence of a CO_2_ channel hAQP5 ΔΔpH_S_ – or ΔΔpH_S,hAQP5_ – is substantially greater. The difference between the two ΔΔpH_S_ values – the ΔΔΔpH_S_ due to the presence of hAQP5 or ΔΔΔpH_S,hAQP5_ – is statistically significant (Table A11*A*).

Figure 11*B* is a rearrangement of two pairs of CO_2_‐influx bars, but with 100 ng of injected hCA II. The ΔΔpH_S,Base_ is similar to the value in Fig. 11*A*. However here the effect of hAQP5 expression, namely ΔΔpH_S,hAQP5_, is even greater. Thus it appears that the greater the level of hCA II the greater is the effect of hAQP5 expression on ΔΔpH_S_. Although this set of comparisons is of CO_2_ influx, we could reach a similar set of conclusions for CO_2_ efflux (i.e. by rearranging bars in Fig. 3*B*).

If we did not know the identity of the membrane protein expressed in these experiments, we could deduce that the protein must have a significant CO_2_ conductance, assuming that the protein itself lacks significant CA activity. We can imagine that the cytosolic CA, by serving as a sink for CO_2_ during influx, sucks CO_2_ into the cell. If $P_{M,CO_{2}}$ is rate‐limiting, the addition of a CO_2_ channel will lead to increased CO_2_ influx, a greater decrease in [CO_2_] at the extracellular cell surface and thus a greater ΔpH_S_.

Note that in a cell in which it is impractical to inject or express a cytosolic CA one might use a drug like acetazolamide to block endogenous CA II and thereby perform an analogous ΔΔΔpH_S_ assay.

### Diagnostic power of ΔΔ(dpH_i_/dt)_Max_ ±bCA in identifying enhanced CO_2_ permeability

**Figure 12:**
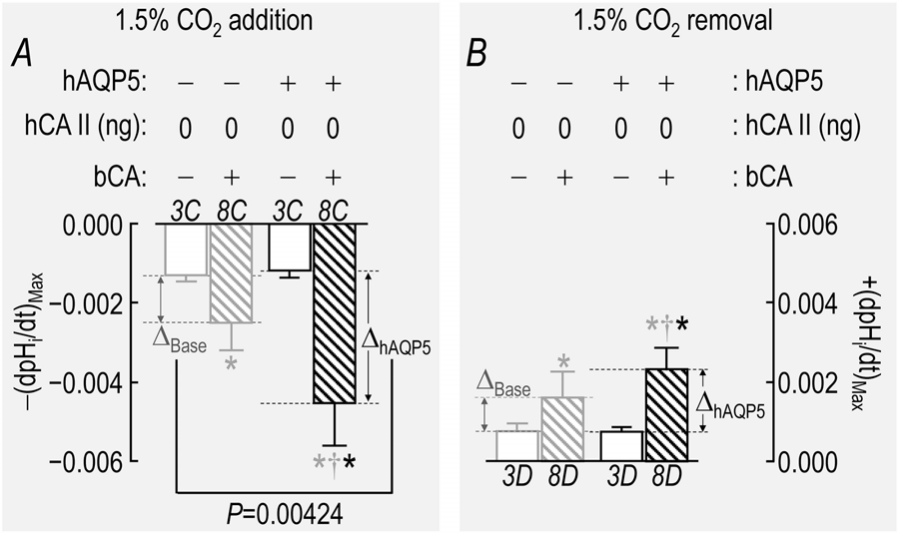
Effect of expressing hAQP5 on Δ(dpH_i_/dt)_Max_: comparison of selected (dpH_i_/dt)_Max_ bars extracted from Fig. 3*C* *vs*. Fig. 8*C* and from Fig. 3*D* *vs*. Fig. 8*D*. Here we rearrange the grey and black bars from Fig. 3*C* (–bCA) and the grey and black bars from Fig. 8*C* (+bCA) to juxtapose two bars representing the same hAQP5 status (‘–’, absent; ‘+’, heterologously expressed): the left bar in a pair representing the absence of bCA and the right bar in a pair representing the presence of bCA in the bECF. A, summary of Δ(dpH_i_/dt)_Max_ ±bCA upon addition of CO_2_/HCO_3_^−^. B, summary of Δ(dpH_i_/dt)_Max_ ±bCA upon removal of CO_2_/HCO_3_^−^. For a pair of bars the difference between (dpH_i_/dt)_Max_ values is Δ(dpH_i_/dt)_Max_. Thus Δ_Base_ is the difference between (dpH_i_/dt)_Max_ values under baseline conditions (i.e. no hAQP5) induced by the addition of the trans‐side CA (i.e. bCA); Δ_hAQP5_ is the corresponding difference between (dpH_i_/dt)_Max_ values in hAQP5‐expressing oocytes. Comparing two pairs of bars the difference between Δ_hAQP5_ and Δ_Base_ values is ΔΔ(dpH_i_/dt)_Max,hAQP5_. Although no hCA II is present in any of these experiments, we include hCA II status (‘0 ng’, not injected) to facilitate comparisons with Fig. 11. Statistical star/bar conventions. See legend of Fig. 3 for a description of statistical significance. The stars indicate statistical significance among individual bars; the grey star associated with the grey‐ and black‐hatched bars indicates a significant difference compared to the open grey bar (–hAQP5, –bCA); the black star associated with the black‐hatched bars (+AQP5, +bCA) indicates a significant difference compared to the open black bar (+hAQP5, –bCA); the grey dagger symbol associated with the black‐hatched bars (+AQP5, +bCA) indicates a significant difference compared to the open grey‐hatched bar (–hAQP5, +bCA). See Table A12*A* and Table A12*B* for the statistics summary (including all the *P*‐values reported by the stars).

In Fig. 12 we perform the inverse analysis of Fig. 11: we rearrange CO_2_‐influx bars from Fig. 3*C* and Fig. 8*C* so that we can easily compare the effects – now on Δ(dpH_i_/dt)_Max_ – of adding a CA – now bCA – on the side of the membrane ‘trans’ to the pH_i_ measurement. The pair of bars on the left of Fig. 12*A* shows that, in the absence of hAQP5, adding bCA to the bECF produces a modest increase in the magnitude of (dpH_i_/dt)_Max_ – the Δ(dpH_i_/dt)_Max,Base_. On the contrary in the presence of the CO_2_ channel hAQP5 the magnitude of (dpH_i_/dt)_Max_ – Δ(dpH_i_/dt)_Max,hAQP5_ – is substantially greater. The difference between the two Δ(dpH_i_/dt)_Max_ values – the ΔΔ(dpH_i_/dt)_Max_ due to the presence of hAQP5 or ΔΔ(dpH_i_/dt)_Max,hAQP5_ – is statistically significant (Table A12*A*).

Figure 12*B* shows a similar analysis for CO_2_/HCO_3_^−^ removal. Because the (dpH_i_/dt)_Max_ values are smaller, the Δ(dpH_i_/dt)_Max_ values also are of smaller magnitude than in Fig. 12*B*. In this case the difference between the two Δ(dpH_i_/dt)_Max_ values is not statistically significant. Thus this assay may not be practical as shown. A future approach could be to raise [CO_2_]_o_, raise [HEPES]_o_, inject 100 ng of hCA II and raise the bCA level (albeit at the increased cost of the enzyme in a continuously flowing solution). Some combination of these changes would increase the rates of pH_i_ change, and thereby make it easier to detect (dpH_i_/dt)_Max_ differences in both the influx and efflux assays.

### Advances and Limitations

#### Synergisms

In the present study we confirm the strong synergy between extracellular and cytosolic CAs (see Figs. 3 and 8). We also identify a strong synergy between a CO_2_ channel (i.e. hAQP5) and a cytosolic CA (i.e. hCA II; see pH_S_ data in Fig. 3). Finally we identify a strong synergy between a CO_2_ channel and an extracellular CA (i.e. bCA; see dpH_i_/dt data in Fig. 8).

#### ‘Cis‐’ *vs*. ‘trans‐side’ effects

Our group has previously pointed out the challenges in pH measurements made ‘cis’ to a CA manipulation (Lu et al., 2006). For example ‘cis‐side’ pH_i_ measurements led to the erroneous conclusion that cytosolic CA II binds to and thereby stimulates the Cl‐HCO_3_ exchanger AE1, while, in fact, the added CA II was catalysing the reaction HCO_3_^−^ + H^+^ → CO_2_ + H_2_O and producing a rapid pH_i_ increase because of catalysis, not transport.

The aforementioned trio of papers (Musa‐Aziz et al., 2014a, 2014b; Occhipinti et al., 2014) systematically deals with the issue of pH measurements made ‘cis’ to manipulations that impact acid–base chemistry (e.g. modulation of CA activity, alterations in non‐HCO_3_^−^ buffering power) and the necessity to focus on ‘trans’ effects. The present paper builds upon and expands these concepts. Note that our modulation of $P_{M,CO_{2}}$ – an ‘inter’ situation – is neither ‘cis’ nor ‘trans’ to our electrodes and is thus immune from these considerations.

#### ‘Inter’ effect

We introduce this term to describe a manoeuvre that impacts the membrane separating two solutions (e.g. intra‐ *vs*. extracellular). For example the act of introducing, deleting, mutating or otherwise changing the activity of an integral membrane protein is ‘in between’ ‘cis’ and ‘trans’. Likewise altering lipid composition or otherwise altering the chemistry of membrane lipids or their interaction with proteins or other substances would be an ‘inter’ effect.

#### pH_S_ relaxation assessed as (dpH_S_/dt)_Max_

Although we have previously assessed pH_S_ relaxation as the time constant of a single‐exponential decay, we now introduce a more general tool for describing the initial rate of pH_S_ decay, especially one that deviates substantially from an SExp time course. Although we implement this tool using a double‐exponential curve fit, in principle we could use any function that near *t*_Local_ = 0 fits pH_S_ *vs*. time with a monotonic decay.

#### Novel diagnostic paradigms

Our approaches for assessing ΔΔΔpH_S_ and ΔΔ(dpH_i_/dt)_Max_ are potentially valuable for evaluating the CO_2_ permeability of candidate membrane proteins.

#### Limitations

A potential drawback of using bCA rather than hCA IV is that, with bCA added to the bECF, chemical or physical factors near the membrane may limit its access to the nanodomain at the membrane surface. On the contrary the use of bCA avoids the issue of ‘ribosome steal’ noted above. Moreover bCA ideally provides CA activity throughout the extracellular unconvected layer. Indeed Musa‐Aziz et al. (2014b) and Occhipinti et al. (2014) showed that bCA II together with hCA IV (the extracellular presence of which is presumably confined to the nanodomain at the membrane surface) is more effective than hCA IV alone.

In the present study, recognizing an already large matrix of experimental conditions, we did not explore bCA levels higher than 0.1 mg/ml, levels that may have generated greater (dpH_i_/dt)_Max_ signals. In our bCA study we did not explore hCA II levels greater than 1 ng, levels that presumably would have generated larger ΔpH_S_ signals.

Unlike the trio of papers noted above the present study does not include assessments of altered extracellular concentrations of (1) CO_2_ (1.5%, 5%, 10% *vs*. 1.5% in present study) or (2) non‐HCO_3_^−^ buffers (1, 5, 25 mM HEPES *vs*. 5 mM here). Such investigations are important because – especially together with the accompanying mathematical model – they provide valuable insights into the impact and interactions of various acid–base buffer members and CAs on transmembrane CO_2_ fluxes. Including such analyses in the present study together with ±hAQP5 would have expanded our experimental matrix to unrealistic levels.

Musa‐Aziz et al. (2014b) found that (1) increasing [CO_2_]_o_ markedly increased both ΔpH_S_ and (dpH_i_/dt)_Max_, especially in the presence of hCA IV (their fig. 10). The effects of increasing [HEPES]_o_ were more complex: (2) with 1.5% CO_2_ raising [HEPES]_o_ predictably decreased ΔpH_S_ (a ‘cis‐side’ effect) but did not significantly affect (dpH_i_/dt)_Max_ (their fig. 13). (3) When they supplemented the hCA IV with extracellular bCA II, still with 1.5% CO_2_, the effect of raising [HEPES]_o_ on (dpH_i_/dt)_Max_ became stronger but still not significant (their fig. 15). However (4) when they worked with 10% CO_2_, the effects of raising [HEPES]_o_ on (dpH_i_/dt)_Max_ were far stronger (their fig. 17).

Based on the above observations we suggest the following for future (dpH_i_/dt)_Max_ experiments on oocytes intended to examine the synergy among hAQP5, CA_i_ and the following: in ΔΔ(dpH_i_/dt)_Max_ protocols it would be helpful to (a) raise [CO_2_]_o_ (e.g. to 10%), (b) explore increasing bCA beyond 0.1 mg/ml and (c) raise [HEPES]_o_ (e.g. to 25 mM). We also suggest that in future ΔΔΔpH_S_ protocols it would be helpful to (a) raise the [CO_2_]_o_ and (b) increase CA_i_ activity (e.g. employing recombinant hCA II, injecting 100 ng or more of purified hCA II).

## Additional information

## Competing interests

All authors declare no conflict of interests.

## Author contributions

D.K.W. contributed to the conception and design of the research, performed the experiments, analysed the data and interpreted the results, wrote the first draft of the manuscript, prepared the figures and edited the manuscript. F.J.M. contributed to the conception and design of the research, interpreted the results, performed statistical analyses and edited the figures and manuscript. W.F.B. contributed to the conception and design of the research, interpreted the results and edited the figures and manuscript. All authors approved the final version of the manuscript; all qualify for authorship, and all those who qualify for authorship are listed.

## Funding

This work was supported by NIH grants HL160857 and DK128315; by Office of Naval Research (ONR) grant N00014‐11‐1‐0889, N00014‐14‐1‐0716 and N00014‐15‐1‐2060; and by a Multidisciplinary University Research Initiative (MURI) grant N00014‐16‐1‐2535 from the Department of Defense (to W.F.B.). The contributions of W.F.B. and F.J.M. to this work were also supported in part by a Department of Defense, Air Force Research Laboratory 711th Human Performance Wing, Studies and Analysis funding 21–023.

## Supporting information


Peer Review History

## Figures and Tables

**Figure A1:**
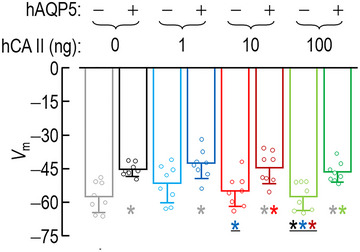
Summary of membrane‐potential (*V*_m_) values for oocytes used in Fig. 3 The bar graph summarizes *V*_m_ values – obtained near the end of experiments (e.g. the rightmost grey band in Fig. 1*C*) – for the oocytes used in Fig. 3. Statistical star/bar conventions: see legend of Fig. 3 for a description of statistical significance. See Table A13 for the statistics summary (including all *P*‐values reported by the stars).

## Data Availability

All raw data are deposited into the NIH‐supported Zenodo data repository in common and open formats and are accessible via the persistent identifier; https://doi.org/10.5281/zenodo.16950950

## Footnotes

1 See ‘Methods’ > ‘Physiological solutions’ > ‘Ross’. Throughout the paper, ‘>’ directs the reader to a subsection of the preceding reference.

2 See ‘Methods’ > ‘Electrophysiological measurements’ > ‘Measurement of pH_S_ and pH_i_’ > ‘Subtraction of electrical signals’.

^3^
See ‘Methods’ > ‘Calculation of ΔpH_S_’, ‘Methods’ > “Determination of ‘maximal’ dpH_i_/dt”, ‘Methods’ > ‘Calculation of intrinsic intracellular buffering power’

^4^
For simplicity, we refer to concentrations in the bECF using the subscript “o”.

5 See ‘Methods’ > ‘Electrophysiological measurements’ > ‘Calibration of pH microelectrodes’.

^6^
Here, ±hAQP5 indicates that experiments in this section compare pH responses in the absence vs. presence of hAQP5.

^7^
See ‘Methods’ > ‘Statistical analysis’ > ‘Calibration of pH microelectrodes’.

^8^
Here, ‘Δ’ indicates that in this section we compare a series of injected doses of hCA II; ‘–bCA’ indicates that extracellular bCA was absent in all experiments.

9 In lists of points in the Summary and Conclusion we use (a), (b), (c) …. The (a) in one list corresponds to the (a) in another list, etc. However in the Interpretation, we list points as (1), (2), (3) … because the interpretations do not necessarily correspond to lettered points in a one‐to‐one fashion.

10 See ‘Results’ > ‘±hAQP5, ΔhCA II, –bCA: effects on ΔpH_S_ and (dpH_i_/dt)_Max_’ > ‘ΔpH_S_’ > ‘Interpretation: ΔpH_S_ (colourful bars) in Figure 3*A,B*; ±hAQP5, ΔhCA II, –bCA’.

11 See ‘Results’ > ‘±hAQP5 in absence of exogenous CAs: effects on ΔpH_S_ and (dpH_i_/dt)_Max_’ > ‘(dpH_i_/dt)_Max_’ > ‘Interpretation: (dpH_i_/dt)_Max_ (grey *vs*. black bars) in Figure 3*C,D*; ±hAQP5, –hCA II, –bCA’.

12 See Results > ‘±hAQP5 in absence of exogenous CAs: effects on ΔpH_S_ and (dpH_i_/dt)_Max_’ > ‘pH_S_’ > ‘Interpretation: ΔpHS (grey *vs*. black bars) in Fig. 3*A,B*; ±hAQP5, −hCA II, −bCA’. Also see Results > ‘±hAQP5, ΔhCA II, –bCA: effects on ΔpH_S_ and (dpH_i_/dt)_Max_’ > ‘pH_S_’ > ‘*Interpretation: ΔpH_S_ (colourful bars) in* Fig. 3*A,B* *; ±hAQP5, –hCA II, –bCA*’.

13 See Results > ‘±hAQP5, –hCA II, +bCA: effects on ΔpH_S_ and (dpH_i_/dt)_Max_’ > ‘pH_S_’ > ‘Interpretation: ΔpH_S_ (grey vs. black bars) in Fig. 8*A,B*: ±hAQP5, –hCA  II, +bCA’.

^14^
See Results > ‘±hAQP5, –hCA II, +bCA: effects on ΔpH_S_ & (dpH_i_/dt)_Max_’ > ‘pH_S_’ > ‘Interpretation: ΔpH_S_ (grey vs. black bars) in Fig. 8*A,B*: ±hAQP5, –hCA  II, +bCA’.

^15^
See Results > ‘±hAQP5, +hCA II, +bCA: effects on ΔpH_S_ and (dpH_i_/dt)_Max_’ > ‘pH_S_’ > ‘Interpretation: ΔpH_S_ (colourful bars) in Fig. 8*A,B*: ±hAQP5, +hCA  II, +bCA’.

^16^
See Results > ‘±hAQP5, +hCA II, +bCA: effects on ΔpH_S_ and (dpH_i_/dt)_Max_’ > ‘pH_S_’ > ‘Interpretation: ΔpH_S_ (colourful bars) in Fig. 8*A,B*: ±hAQP5, +hCA  II, +bCA’.

## Appendix A

**Table A3A:** Statistics summary for Fig. 3*A*: (i) descriptive statistics for data presented in the figure panel, ∆pH_S_ upon CO_2_ addition; (ii) overall one‐way ANOVA results; (iii) Tukey's means comparison analysis

i. Descriptive statistics				
	*n*	Mean	SD	SEM
H_2_O + Tris	8	0.0285	0.00895	0.00316
hAQP5 + Tris	8	0.0787	0.0181	0.00641
H_2_O + 1 ng hCA II	8	0.0411	0.0115	0.00406
hAQP5 + 1 ng hCA II	8	0.127	0.0246	0.00869
H_2_O + 10 ng hCA II	8	0.0418	0.00938	0.00332
hAQP5 + 10 ng hCA II	8	0.131	0.0262	0.00925
H_2_O + 100 ng hCA II	8	0.0416	0.00948	0.00335
hAQP5 + 100 ng hCA II	8	0.160	0.0478	0.0169

ii. Overall ANOVA					
Variation	DF	Sum of squares	Mean square	*F*‐value	*P*‐value
Between groups	7	0.147	0.0210	39.2	2.44 × 10^−19^
Within groups	56	0.0301	5.37 × 10^−4^		
Total	63	0.177			

iii. Tukey's test							
Condition A	Condition B	Mean diff.	*Q*‐value	*P*‐value	Sig.	LCL	UCL
hAQP5 + Tris	H_2_O + Tris	0.0502	6.13	0.00150	✓	0.0137	0.0867
H_2_O + 1 ng hCA II	H_2_O + Tris	0.0126	1.54	0.956	–	−0.0238	0.0491
H_2_O + 1 ng hCA II	hAQP5 + Tris	−0.0376	4.59	0.0391	✓	−0.0741	−0.00110
hAQP5 + 1 ng hCA II	H_2_O + Tris	0.0986	12.0	4.18 × 10^−8^	✓	0.0622	0.135
hAQP5 + 1 ng hCA II	hAQP5 + Tris	0.0484	5.91	0.00247	✓	0.0120	0.0849
hAQP5 + 1 ng hCA II	H_2_O + 1ng hCA II	0.0860	10.5	7.19 × 10^−8^	✓	0.0495	0.122
H_2_O + 10 ng hCA II	H_2_O + Tris	0.0133	1.63	0.943	–	−0.0232	0.0498
H_2_O + 10 ng hCA II	hAQP5 + Tris	−0.0369	4.50	0.0456	✓	−0.0734	−4.10×10^−4^
H_2_O + 10 ng hCA II	H_2_O + 1ng hCA II	6.88 × 10^−4^	0.0839	1.00	−	−0.0358	0.0372
H_2_O + 10 ng hCA II	hAQP5 + 1ng hCA II	−0.0853	10.4	7.72 × 10^−8^	✓	−0.122	−0.0488
hAQP5 + 10 ng hCA II	H_2_O + Tris	0.103	12.6	3.90 × 10^−8^	✓	0.0664	0.139
hAQP5 + 10 ng hCA II	hAQP5 + Tris	0.0527	6.43	7.38 × 10^−4^	✓	0.0162	0.0892
hAQP5 + 10 ng hCA II	H_2_O + 1 ng hCA II	0.0902	11.0	5.43 × 10^−8^	✓	0.0538	0.127
hAQP5 + 10 ng hCA II	hAQP5 + 1 ng hCA II	0.00424	0.518	1.00	–	−0.0322	0.0407
hAQP5 + 10 ng hCA II	H_2_O + 10 ng hCA II	0.0896	10.9	5.60 × 10^−8^	✓	0.0531	0.126
H_2_O + 100 ng hCA II	H_2_O + Tris	0.0131	1.60	0.947	–	−0.0234	0.0496
H_2_O + 100 ng hCA II	hAQP5 + Tris	−0.0371	4.53	0.0437	✓	−0.0736	−6.01×10^−4^
H_2_O + 100 ng hCA II	H_2_O + 1 ng hCA II	4.96 × 10^−4^	0.0606	1.00	–	−0.0360	0.0370
H_2_O + 100 ng hCA II	hAQP5 + 1 ng hCA II	−0.0855	10.4	7.56 × 10^−8^	✓	−0.122	−0.0490
H_2_O + 100 ng hCA II	H_2_O + 10 ng hCA II	−1.91×10^−4^	0.0233	1.00	–	−0.0367	0.0363
H_2_O + 100 ng hCA II	hAQP5 + 10 ng hCA II	−0.0898	11.0	5.55 × 10^−8^	✓	−0.126	−0.0533
hAQP5 +100 ng hCA II	H_2_O + Tris	0.132	16.1	2.21 × 10^−8^	✓	0.0955	0.168
hAQP5 +100 ng hCA II	hAQP5 + Tris	0.0817	9.98	1.34 × 10^−7^	✓	0.0453	0.118
hAQP5 +100 ng hCA II	H_2_O + 1 ng hCA II	0.119	14.6	2.93 × 10^−8^	✓	0.0828	0.156
hAQP5 +100 ng hCA II	hAQP5 + 1 ng hCA II	0.0333	4.06	0.0978	–	−0.00318	0.0698
hAQP5 +100 ng hCA II	H_2_O + 10 ng hCA II	0.119	14.5	2.97 × 10^−8^	✓	0.0821	0.155
hAQP5 +100 ng hCA II	hAQP5 + 10 ng hCA II	0.0291	3.55	0.214	–	−0.00742	0.0655
hAQP5 +100 ng hCA II	H_2_O + 100 ng hCA II	0.119	14.5	2.95 × 10^−8^	✓	0.0823	0.155

*Notes*: Threshold for significance a = 0.05. Standard error of the mean = 0.0116.

Abbreviations: DF, degrees of freedom; LCL, lower confidence limit; mean diff., mean difference for each comparison; UCL, upper confidence limit.

**Table A3B:** Statistics summary for Fig. 3*B*: (i) descriptive statistics for data presented in the figure panel, ∆pH_S_ upon CO_2_ removal; (ii) overall one‐way ANOVA results; (iii) Tukey's means comparison analysis

i. Descriptive statistics				
	*n*	Mean	SD	SEM
H_2_O + Tris	8	−0.03093	0.00994	0.00352
hAQP5 + Tris	8	−0.09598	0.02688	0.0095
H_2_O + 1 ng hCA II	8	−0.04167	0.01131	0.004
hAQP5 + 1 ng hCA II	8	−0.13982	0.02817	0.00996
H_2_O + 10 ng hCA II	8	−0.04078	0.01032	0.00365
hAQP5 + 10 ng hCA II	8	−0.13894	0.03249	0.01149
H_2_O + 100 ng hCA II	8	−0.04123	0.0107	0.00378
hAQP5 + 100 ng hCA II	8	−0.16218	0.05069	0.01792

ii. Overall ANOVA					
Variation	DF	Sum of squares	Mean square	*F*‐value	*P*‐value
Between groups	7	0.165	0.0236	33.8	6.93 × 10^−18^
Within groups	56	0.0391	6.99×10^−4^		
Total	63	0.204			

iii. Tukey's test							
Condition A	Condition B	Mean diff.	*Q*‐value	*P*‐value	Sig.	LCL	UCL
hAQP5 + Tris	H_2_O + Tris	−0.0650	6.96	2.03 × 10^−4^	✓	−0.107	−0.0234
H_2_O + 1 ng hCA II	H_2_O + Tris	−0.0107	1.15	0.992	–	−0.0524	0.0309
H_2_O + 1 ng hCA II	hAQP5 + Tris	0.0543	5.81	0.00309	✓	0.0127	0.0959
hAQP5 + 1 ng hCA II	H_2_O + Tris	−0.109	11.7	4.44 × 10^−8^	✓	−0.150	−0.0673
hAQP5 + 1 ng hCA II	hAQP5 + Tris	−0.0438	4.69	0.0321	✓	−0.0854	−0.00223
hAQP5 + 1 ng hCA II	H_2_O + 1 ng hCA II	−0.0981	10.5	7.16 × 10^−8^	✓	−0.140	−0.0565
H_2_O + 10 ng hCA II	H_2_O + Tris	−0.00985	1.05	0.995	–	−0.0515	0.0318
H_2_O + 10 ng hCA II	hAQP5 + Tris	0.0552	5.91	0.00249	✓	0.0136	0.0968
H_2_O + 10 ng hCA II	H_2_O + 1 ng hCA II	8.96 × 10^−4^	0.0959	1.00	–	−0.0407	0.0425
H_2_O + 10 ng hCA II	hAQP5 + 1 ng hCA II	0.0990	10.6	6.67 × 10^−8^	✓	0.0574	0.141
hAQP5 + 10 ng hCA II	H_2_O + Tris	−0.108	11.6	4.51 × 10^−8^	✓	−0.150	−0.0664
hAQP5 + 10 ng hCA II	hAQP5 + Tris	−0.0430	4.60	0.0383	✓	−0.0846	−0.00136
hAQP5 + 10 ng hCA II	H_2_O + 1 ng hCA II	−0.0973	10.4	7.76 × 10^−8^	✓	−0.139	−0.0557
hAQP5 + 10 ng hCA II	hAQP5 + 1 ng hCA II	8.74 × 10^−4^	0.0935	1.00	–	−0.0407	0.0425
hAQP5 + 10 ng hCA II	H_2_O + 10 ng hCA II	−0.0982	10.5	7.15 × 10^−8^	✓	−0.140	−0.0566
H_2_O + 100 ng hCA II	H_2_O + Tris	−0.0103	1.10	0.994	–	−0.0519	0.0313
H_2_O + 100 ng hCA II	hAQP5 + Tris	0.0547	5.86	0.00278	✓	0.0131	0.0964
H_2_O + 100 ng hCA II	H_2_O + 1 ng hCA II	4.40 × 10^−4^	0.0471	1.00	–	−0.0412	0.0420
H_2_O + 100 ng hCA II	hAQP5 + 1 ng hCA II	0.0986	10.5	6.91 × 10^−8^	✓	0.0570	0.140
H_2_O + 100 ng hCA II	H_2_O + 10 ng hCA II	−4.56 × 10^−4^	0.0488	1.00	–	−0.0421	0.0412
H_2_O + 100 ng hCA II	hAQP5 + 10ng hCA II	0.0977	10.5	7.44 × 10^−8^	✓	0.0561	0.139
hAQP5 +100 ng hCA II	H_2_O + Tris	−0.131	14.0	3.17 × 10^−8^	✓	−0.173	−0.0896
hAQP5 +100 ng hCA II	hAQP5 + Tris	−0.0662	7.08	1.50 × 10^−4^	✓	−0.108	−0.0246
hAQP5 +100 ng hCA II	H_2_O + 1 ng hCA II	−0.121	12.9	3.72 × 10^−8^	✓	−0.162	−0.0789
hAQP5 +100 ng hCA II	hAQP5 + 1 ng hCA II	−0.0224	2.39	0.692	–	−0.0640	0.0192
hAQP5 +100 ng hCA II	H_2_O + 10 ng hCA II	−0.121	13.0	3.68 × 10^−8^	✓	−0.163	−0.0798
hAQP5 +100 ng hCA II	hAQP5 + 10 ng hCA II	−0.0232	2.49	0.650	–	−0.0648	0.0184
hAQP5 +100 ng hCA II	H_2_O + 100 ng hCA II	−0.121	12.9	3.70 × 10^−8^	✓	−0.163	−0.0793

*Notes*: Threshold for significance a = 0.05. Standard error of the mean = 0.0132.

Abbreviations: DF, degrees of freedom; LCL, lower confidence limit; mean diff., mean difference for each comparison; UCL, upper confidence limit.

**Table A3C:** Statistics summary for Fig. 3*C*: (i) descriptive statistics for data presented in the figure panel, (dpH_i_/dt)_Max_ upon CO_2_ addition; (ii) overall one‐way ANOVA results. (iii) Tukey's means comparison analysis

i. Descriptive statistics				
	*n*	Mean	SD	SEM
H_2_O + Tris	8	−0.00126	2.08 × 10^−4^	7.35 × 10^−5^
hAQP5 + Tris	8	−0.00122	1.57 × 10^−4^	5.55 × 10^−5^
H_2_O + 1 ng hCA II	8	−0.00458	0.00214	7.57 × 10^−4^
hAQP5 + 1 ng hCA II	8	−0.00553	0.00171	6.06 × 10^−4^
H_2_O + 10 ng hCA II	8	−0.00660	0.00293	0.00104
hAQP5 + 10 ng hCA II	8	−0.00732	0.00236	8.36 × 10^−4^
H_2_O + 100 ng hCA II	8	−0.00645	0.0029	0.00102
hAQP5 + 100 ng hCA II	8	−0.00601	0.00318	0.00113

ii. Overall ANOVA					
Variation	DF	Sum of squares	Mean square	*F*‐value	*P*‐value
Between groups	7	3.17 × 10^−4^	4.53 × 10^−5^	8.98	2.25 × 10^−7^
Within groups	56	2.82 × 10^−4^	5.04 × 10^−6^		
Total	63	5.99 × 10^−4^			

iii. Tukey's test							
Condition A	Condition B	Mean diff.	*Q*‐value	*P*‐value	Sig.	LCL	UCL
hAQP5 + Tris	H_2_O + Tris	3.53 × 10^−5^	0.0445	1.00	–	−0.00350	0.00357
H_2_O + 1 ng hCA II	H_2_O + Tris	−0.00332	4.18	0.0803	–	−0.00685	2.14 × 10^−4^
H_2_O + 1 ng hCA II	hAQP5 + Tris	−0.00335	4.23	0.0744	–	−0.00689	1.79×10^−4^
hAQP5 + 1 ng hCA II	H_2_O + Tris	−0.00427	5.38	0.00798	✓	−0.00780	−7.35 × 10^−4^
hAQP5 + 1 ng hCA II	hAQP5 + Tris	−0.00430	5.42	0.00726	✓	−0.00784	−7.71 × 10^−4^
hAQP5 + 1 ng hCA II	H_2_O + 1 ng hCA II	−9.50 × 10^−4^	1.20	0.989	–	−0.00448	0.00258
H_2_O + 10 ng hCA II	H_2_O + Tris	−0.00534	6.73	3.62 × 10^−4^	✓	−0.00887	−0.00180
H_2_O + 10 ng hCA II	hAQP5 + Tris	−0.00537	6.77	3.25 × 10^−4^	✓	−0.00891	−0.00184
H_2_O + 10 ng hCA II	H_2_O + 1 ng hCA II	−0.00202	2.54	0.624	–	−0.00555	0.00152
H_2_O + 10 ng hCA II	hAQP5 + 1 ng hCA II	−0.00107	1.35	0.979	–	−0.00460	0.00247
hAQP5 + 10 ng hCA II	H_2_O + Tris	−0.00606	7.63	3.76 × 10^−5^	✓	−0.00959	−0.00252
hAQP5 + 10 ng hCA II	hAQP5 + Tris	−0.00609	7.68	3.36 × 10^−5^	✓	−0.00963	−0.00256
hAQP5 + 10 ng hCA II	H_2_O + 1 ng hCA II	−0.00274	3.45	0.244	–	−0.00627	7.97×10^−4^
hAQP5 + 10 ng hCA II	hAQP5 + 1 ng hCA II	−0.00179	2.25	0.753	–	−0.00532	0.00175
hAQP5 + 10 ng hCA II	H_2_O + 10 ng hCA II	−7.19 × 10^−4^	0.906	0.998	–	−0.00425	0.00281
H_2_O + 100 ng hCA II	H_2_O + Tris	−0.00519	6.54	5.69 × 10^−4^	✓	−0.00872	−0.00166
H_2_O + 100 ng hCA II	hAQP5 + Tris	−0.00522	6.58	5.11 × 10^−4^	✓	−0.00876	−0.00169
H_2_O + 100 ng hCA II	H_2_O + 1ng hCA II	−0.00187	2.36	0.708	–	−0.00540	0.00166
H_2_O + 100 ng hCA II	hAQP5 + 1ng hCA II	−9.20×10^−4^	1.16	0.991	–	−0.00445	0.00261
H_2_O + 100 ng hCA II	H_2_O + 10ng hCA II	1.48×10^−4^	0.187	1.00	–	−0.00339	0.00368
H_2_O + 100 ng hCA II	hAQP5 + 10ng hCA II	8.67×10^−4^	1.09	0.994	–	−0.00267	0.00440
hAQP5 +10 0ng hCA II	H_2_O + Tris	−0.00475	5.98	0.00210	✓	−0.00828	−0.00121
hAQP5 +100 ng hCA II	hAQP5 + Tris	−0.00478	6.03	0.00189	✓	−0.00832	−0.00125
hAQP5 +100 ng hCA II	H_2_O + 1ng hCA II	−0.00143	1.80	0.905	–	−0.00496	0.00210
hAQP5 +100 ng hCA II	hAQP5 + 1ng hCA II	−4.79×10^−4^	0.604	1.00	–	−0.00401	0.00305
hAQP5 +100 ng hCA II	H_2_O + 10ng hCA II	5.89×10^−4^	0.743	0.999	–	−0.00294	0.00412
hAQP5 +100 ng hCA II	hAQP5 + 10ng hCA II	0.00131	1.65	0.938	–	−0.00223	0.00484
hAQP5 +100 ng hCA II	H_2_O + 100ng hCA II	4.41×10^−4^	0.556	1.00	–	−0.00309	0.00397

*Notes*: Threshold for significance a = 0.05. Standard error of the mean = 0.0112.

Abbreviations: DF, degrees of freedom; LCL, lower confidence limit; mean diff., mean difference for each comparison; UCL, upper confidence limit.

**Table A3D:** Statistics summary for Fig. 3*D*: (i) descriptive statistics for data presented in the figure panel, (dpH_i_/dt)_Max_ upon CO_2_ removal; (ii) overall one‐way ANOVA results. (iii) Tukey's means comparison analysis

i. Descriptive statistics				
	n	Mean	SD	SEM
H_2_O + Tris	8	8.35 × 10^−4^	1.34 × 10^−4^	4.74 × 10^−5^
hAQP5 + Tris	8	8.07 × 10^−4^	6.85 × 10^−5^	2.42 × 10^−5^
H_2_O + 1 ng hCA II	8	0.00263	0.00119	4.19 × 10^−4^
hAQP5 + 1 ng hCA II	8	0.00306	8.73 × 10^−4^	3.08 × 10^−4^
H_2_O + 10 ng hCA II	8	0.00355	0.00121	4.29 × 10^−4^
hAQP5 + 10 ng hCA II	8	0.00399	0.00139	4.92 × 10^−4^
H_2_O + 100 ng hCA II	8	0.00373	0.00266	9.39 × 10^−4^
hAQP5 + 100 ng hCA II	8	0.00297	0.00134	4.74 × 10^−4^

ii. Overall ANOVA					
Variation	DF	Sum of squares	Mean square	*F*‐value	*P*‐value
Between groups	7	8.57 × 10^−5^	1.22 × 10^−5^	6.78	7.89 × 10^−6^
Within groups	56	1.01 × 10^−4^	1.81 × 10^−6^		
Total	63	1.87 × 10^−4^			

iii. Tukey's test							
Condition A	Condition B	Mean diff.	*Q*‐value	*P*‐value	Sig.	LCL	UCL
hAQP5 + Tris	H_2_O + Tris	−2.75 × 10^−5^	0.0579	1.00	–	−0.00214	0.00209
H_2_O + 1 ng hCA II	H_2_O + Tris	0.00179	3.77	0.154	–	−3.22 × 10^−4^	0.00391
H_2_O + 1 ng hCA II	hAQP5 + Tris	0.00182	3.83	0.141	–	−2.95 × 10^−4^	0.00394
hAQP5 + 1 ng hCA II	H_2_O + Tris	0.00222	4.68	0.0329	✓	1.07 × 10^−4^	0.00434
hAQP5 + 1 ng hCA II	hAQP5 + Tris	0.00225	4.74	0.0295	✓	1.34 × 10^−4^	0.00437
hAQP5 + 1 ng hCA II	H_2_O + 1 ng hCA II	4.29 × 10^−4^	0.903	0.998	–	−0.00169	0.00255
H_2_O + 10 ng hCA II	H_2_O + Tris	0.00272	5.72	0.00381	✓	6.02 × 10^−4^	0.00483
H_2_O + 10 ng hCA II	hAQP5 + Tris	0.00275	5.78	0.00335	✓	6.29 × 10^−4^	0.00486
H_2_O + 10 ng hCA II	H_2_O + 1ng hCA II	9.24 × 10^−4^	1.94	0.865	–	−0.00119	0.00304
H_2_O + 10 ng hCA II	hAQP5 + 1ng hCA II	4.95 × 10^−4^	1.04	0.995	–	−0.00162	0.00261
hAQP5 + 10 ng hCA II	H_2_O + Tris	0.00315	6.63	4.57 × 10^−4^	✓	0.00103	0.00527
hAQP5 + 10 ng hCA II	hAQP5 + Tris	0.00318	6.69	3.97 × 10^−4^	✓	0.00106	0.00529
hAQP5 + 10 ng hCA II	H_2_O + 1 ng hCA II	0.00136	2.85	0.479	–	−7.59 × 10^−4^	0.00347
hAQP5 + 10 ng hCA II	hAQP5 + 1 ng hCA II	9.28×10^−4^	1.95	0.862	–	−0.00119	0.00304
hAQP5 + 10 ng hCA II	H_2_O + 10 ng hCA II	4.33×10^−4^	0.911	0.998	–	−0.00168	0.00255
H_2_O + 100ng hCA II	H_2_O + Tris	0.00290	6.10	0.00160	✓	7.84 × 10^−4^	0.00502
H_2_O + 100ng hCA II	hAQP5 + Tris	0.00293	6.16	0.00140	✓	8.11 × 10^−4^	0.00504
H_2_O + 100ng hCA II	H_2_O + 1 ng hCA II	0.00111	2.33	0.721	–	−0.00101	0.00322
H_2_O + 100ng hCA II	hAQP5 + 1 ng hCA II	6.77 × 10^−4^	1.42	0.972	–	−0.00144	0.00279
H_2_O + 100ng hCA II	H_2_O + 10 ng hCA II	1.82 × 10^−4^	0.383	1.00	–	−0.00193	0.00230
H_2_O + 100ng hCA II	hAQP5 + 10 ng hCA II	−2.51 × 10^−4^	0.528	1.00	–	−0.00237	0.00187
hAQP5 +100ng hCA II	H_2_O + Tris	0.00214	4.50	0.0460	✓	2.19 × 10^−5^	0.00425
hAQP5 +100ng hCA II	hAQP5 + Tris	0.00217	4.56	0.0413	✓	4.94 × 10^−5^	0.00428
hAQP5 +100ng hCA II	H_2_O + 1 ng hCA II	3.44 × 10^−4^	0.724	1.00	–	−0.00177	0.00246
hAQP5 +100ng hCA II	hAQP5 + 1 ng hCA II	−8.49 × 10^−5^	0.179	1.00	–	−0.00220	0.00203
hAQP5 +100ng hCA II	H_2_O + 10 ng hCA II	−5.80 × 10^−4^	1.22	0.988	–	−0.00270	0.00154
hAQP5 +100ng hCA II	hAQP5 + 10 ng hCA II	−0.00101	2.13	0.801	–	−0.00313	0.00110
hAQP5 +100ng hCA II	H_2_O + 100 ng hCA II	−7.62 × 10^−4^	1.60	0.947	–	−0.00288	0.00135

*Notes*: Threshold for significance a = 0.05. Standard error of the mean = 6.27 × 10^−4^.

Abbreviations: DF, degrees of freedom; LCL, lower confidence limit; mean diff., mean difference for each comparison; UCL, upper confidence limit.

**Table A5A:** Statistics summary for Fig. 5*A*: (i) descriptive statistics for data presented in the figure panel, (dpH_S_/dt)_Max_ upon CO_2_ addition; (ii) overall one‐way ANOVA results; (iii) Tukey's means comparison analysis

i. Descriptive statistics				
	*n*	Mean	SD	SEM
H_2_O + Tris	8	−6.14×10^−4^	1.81×10^−4^	6.39×10^−5^
hAQP5 + Tris	10	−0.00171	2.19×10^−4^	6.92×10^−5^
H_2_O + 1 ng h CAII	8	−7.81×10^−4^	3.15×10^−4^	1.11×10^−4^
hAQP5 + 1 ng h CAII	15	−0.00230	4.30×10^−4^	1.11×10^−4^
H_2_O + 10 ng h CAII	10	−8.51×10^−4^	1.48×10^−4^	4.66×10^−5^
hAQP5 + 10 ng h CAII	9	−0.00275	6.86×10^−4^	2.29×10^−4^
H_2_O + 100 ng h CAII	7	−8.21×10^−4^	2.07×10^−4^	7.84×10^−5^
hAQP5 + 100 ng h CAII	12	−0.00379	9.53×10^−4^	2.75×10^−4^

ii. Overall ANOVA					
Variation	DF	Sum of squares	Mean square	*F*‐value	*P*‐value
Between groups	7	9.44×10^−5^	1.35×10^−5^	52.7	1.27×10^−25^
Within groups	71	1.82×10^−5^	2.56×10^−7^		
Total	78	1.13×10^−4^			

iii. Tukey's test								
Condition A	Condition B	Mean diff.	SEM	*Q*‐value	*P*‐value	Sig.	LCL	UCL
hAQP5 + Tris	H_2_O + Tris	−0.00110	2.40×10^−4^	6.49	4.79×10^−4^	✓	−0.00214	0.00209
H_2_O + 1 ng hCA II	H_2_O + Tris	−1.67×10^−4^	2.53×10^−4^	0.933	0.998	–	−3.22×10^−4^	0.00391
H_2_O + 1 ng hCA II	hAQP5 + Tris	9.34×10^−4^	2.40×10^−4^	5.50	0.00518	✓	−2.95×10^−4^	0.00394
hAQP5 + 1 ng hCA II	H_2_O + Tris	−0.00168	2.21×10^−4^	10.8	4.73×10^−8^	✓	1.07×10^−4^	0.00434
hAQP5 + 1 ng hCA II	hAQP5 + Tris	−5.84×10^−4^	2.06×10^−4^	4.00	0.104	–	1.34×10^−4^	0.00437
hAQP5 + 1 ng hCA II	H_2_O + 1ng hCA II	−0.00152	2.21×10^−4^	9.69	7.84×10^−8^	✓	−0.00169	0.00255
H_2_O + 10 ng hCA II	H_2_O + Tris	−2.37×10^−4^	2.40×10^−4^	1.40	0.975	–	6.02×10^−4^	0.00483
H_2_O + 10 ng hCA II	hAQP5 + Tris	8.64×10^−4^	2.26×10^−4^	5.40	0.00655	✓	6.29×10^−4^	0.00486
H_2_O + 10 ng hCA II	H_2_O + 1 ng hCA II	−7.00×10^−5^	2.40×10^−4^	0.413	1.000	–	−0.00119	0.00304
H_2_O + 10 ng hCA II	hAQP5 + 1 ng hCA II	0.00145	2.06×10^−4^	9.92	4.77×10^−8^	✓	−0.00162	0.00261
hAQP5 + 10 ng hCA II	H_2_O + Tris	−0.00214	2.46×10^−4^	12.3	3.42×10^−8^	✓	0.00103	0.00527
hAQP5 + 10 ng hCA II	hAQP5 + Tris	−0.00104	2.32×10^−4^	6.32	7.41×10^−4^	✓	0.00106	0.00529
hAQP5 + 10 ng hCA II	H_2_O + 1 ng hCA II	−0.00197	2.46×10^−4^	11.3	4.18×10^−8^	✓	−7.59×10^−4^	0.00347
hAQP5 + 10 ng hCA II	hAQP5 + 1 ng hCA II	−4.54×10^−4^	2.13×10^−4^	3.01	0.408	–	−0.00119	0.00304
hAQP5 + 10 ng hCA II	H_2_O + 10ng hCA II	−0.00190	2.32×10^−4^	11.6	4.03×10^−8^	✓	−0.00168	0.00255
H_2_O + 100 ng hCA II	H_2_O + Tris	−2.07×10^−4^	2.62×10^−4^	1.12	0.993	–	7.84×10^−4^	0.00502
H_2_O + 100ng hCA II	hAQP5 + Tris	8.94×10^−4^	2.49×10^−4^	5.07	0.0134	✓	8.11×10^−4^	0.00504
H_2_O + 100 ng hCA II	H_2_O + 1 ng hCA II	−3.97×10^−5^	2.62×10^−4^	0.215	1.000	–	−0.00101	0.00322
H_2_O + 100 ng hCA II	hAQP5 + 1ng hCA II	0.00148	2.32×10^−4^	9.03	4.89×10^−7^	✓	−0.00144	0.00279
H_2_O + 100ng hCA II	H_2_O + 10ng hCA II	3.03×10^−5^	2.49×10^−4^	0.172	1.000	–	−0.00193	0.00230
H_2_O + 100 ng hCA II	hAQP5 + 10 ng hCA II	0.00193	2.55×10^−4^	10.7	4.78×10^−8^	✓	−0.00237	0.00187
hAQP5 +100 ng hCA II	H_2_O + Tris	−0.00317	2.31×10^−4^	19.4	0.00	✓	2.19×10^−5^	0.00425
hAQP5 +100 ng hCA II	hAQP5 + Tris	−0.00207	2.17×10^−4^	13.5	2.85×10^−8^	✓	4.94×10^−5^	0.00428
hAQP5 +100 ng hCA II	H_2_O + 1 ng hCA II	−0.00301	2.31×10^−4^	18.4	0.00	✓	−0.00177	0.00246
hAQP5 +100 ng hCA II	hAQP5 + 1 ng hCA II	−0.00149	1.96×10^−4^	10.7	4.74×10^−8^	✓	−0.00220	0.00203
hAQP5 +100 ng hCA II	H_2_O + 10 ng hCA II	−0.00294	2.17×10^−4^	19.2	0.00	✓	−0.00270	0.00154
hAQP5 +100 ng hCA II	hAQP5 + 10 ng hCA II	−0.00104	2.23×10^−4^	6.56	3.95×10^−4^	✓	−0.00313	0.00110
hAQP5 +100 ng hCA II	H_2_O + 100 ng hCA II	−0.00297	2.41×10^−4^	17.4	0.00	✓	−0.00288	0.00135

*Notes*: Threshold for significance a = 0.05. Where the displayed P‐values is 0.00, this indicates a values less than <2.22 × 10^−308^. 2.22×10^−308^ is the smallest possible value for double type data that a 64‐bit system is able to distinguish.

Abbreviations: DF, degrees of freedom; LCL, lower confidence limit; mean diff., mean difference for each comparison; UCL, upper confidence limit.

**Table A5B:** Statistics summary for Fig. 5*B*: (i) descriptive statistics for data presented in the figure panel, (dpH_S_/dt)_Max_ upon CO_2_ removal; (ii) overall one‐way ANOVA results for data presented in Fig. 5*A*; (iii) Tukey's means comparison analysis

i. Descriptive statistics				
	*n*	Mean	SD	SEM
H_2_O + Tris	8	4.13×10^−4^	2.42×10^−4^	8.55×10^−5^
hAQP5 + Tris	10	0.00136	8.19×10^−4^	2.59×10^−4^
H_2_O + 1 ng hCA II	8	5.84×10^−4^	3.49×10^−4^	1.23×10^−4^
hAQP5 + 1 ng hCA II	15	0.00151	6.52×10^−4^	1.68×10^−4^
H_2_O + 10 ng hCA II	10	6.17×10^−4^	1.45×10^−4^	4.60×10^−5^
hAQP5 + 10 ng hCA II	9	0.00157	6.00×10^−4^	2.00×10^−4^
H_2_O + 100 ng hCA II	7	6.33×10^−4^	1.66×10^−4^	6.28×10^−5^
hAQP5 + 100 ng hCA II	12	0.00224	7.43×10^−4^	2.14×10^−4^

ii. Overall ANOVA					
Variation	DF	Sum of squares	Mean square	*F*‐value	*P*‐value
Between groups	7	2.96×10^−5^	4.23×10^−6^	13.3	7.77×10^−11^
Within groups	71	2.26×10^−5^	3.18×10^−7^		
Total	78	5.22×10^−5^			

iii. Tukey's test								
Condition A	Condition B	Mean diff.	SEM	*Q*‐value	*P*‐value	Sig.	LCL	UCL
hAQP5 + Tris	H_2_O + Tris	9.49×10^−4^	2.67×10^−4^	5.02	0.0151	✓	1.14×10^−4^	0.00178
H_2_O + 1 ng hCA II	H_2_O + Tris	1.71×10^−4^	2.82×10^−4^	0.859	0.999	–	−7.09×10^−4^	0.00105
H_2_O + 1 ng hCA II	hAQP5 + Tris	−7.78×10^−4^	2.67×10^−4^	4.11	0.0862	–	−0.00161	5.76×10^−5^
hAQP5 + 1 ng hCA II	H_2_O + Tris	0.00110	2.47×10^−4^	6.30	7.67×10^−4^	✓	3.29×10^−4^	0.00187
hAQP5 + 1 ng hCA II	hAQP5 + Tris	1.51×10^−4^	2.30×10^−4^	0.928	0.998	–	−5.68×10^−4^	8.70×10^−4^
hAQP5 + 1 ng hCA II	H_2_O + 1ng hCA II	9.29×10^−4^	2.47×10^−4^	5.32	0.00783	✓	1.58×10^−4^	0.00170
H_2_O + 10 ng hCA II	H_2_O + Tris	2.04×10^−4^	2.67×10^−4^	1.08	0.995	–	−6.31×10^−4^	0.00104
H_2_O + 10 ng hCA II	hAQP5 + Tris	−7.45×10^−4^	2.52×10^−4^	4.18	0.0768	–	−0.00153	4.23×10^−5^
H_2_O + 10 ng hCA II	H_2_O + 1 ng hCA II	3.25×10^−5^	2.67×10^−4^	0.172	1.000	–	−8.03×10^−4^	8.68×10^−4^
H_2_O + 10 ng hCA II	hAQP5 + 1 ng hCA II	−8.96×10^−4^	2.30×10^−4^	5.51	0.00516	✓	−0.00161	−1.77×10^−4^
hAQP5 + 10 ng hCA II	H_2_O + Tris	0.00116	2.74×10^−4^	5.99	0.00166	✓	3.04×10^−4^	0.00202
hAQP5 + 10 ng hCA II	hAQP5 + Tris	2.11×10^−4^	2.59×10^−4^	1.15	0.992	–	−5.98×10^−4^	0.00102
hAQP5 + 10 ng hCA II	H_2_O + 1 ng hCA II	9.89×10^−4^	2.74×10^−4^	5.11	0.0125	✓	1.33×10^−4^	0.00184
hAQP5 + 10 ng hCA II	hAQP5 + 1 ng hCA II	6.02×10^−5^	2.38×10^−4^	0.358	1.000	–	−6.82×10^−4^	8.03×10^−4^
hAQP5 + 10 ng hCA II	H_2_O + 10ng hCA II	9.56×10^−4^	2.59×10^−4^	5.22	0.00975	✓	1.47×10^−4^	0.00177
H_2_O + 100 ng hCA II	H_2_O + Tris	2.20×10^−4^4	2.92×10^−4^	1.07	0.995	–	−6.91×10^−4^	0.00113
H_2_O + 100ng hCA II	hAQP5 + Tris	−7.28×10^−4^	2.78×10^−4^	3.71	0.165	–	−0.00160	1.39×10^−4^
H_2_O + 100 ng hCA II	H_2_O + 1 ng hCA II	4.91×10^−5^	2.92×10^−4^	0.238	1.000	–	−8.62×10^−4^	9.60×10^−4^
H_2_O + 100 ng hCA II	hAQP5 + 1ng hCA II	−8.79×10^−4^	2.58×10^−4^	4.82	0.0228	✓	−0.00169	−7.36E‐5
H_2_O + 100ng hCA II	H_2_O + 10ng hCA II	1.66×10^−5^	2.78×10^−4^	0.0847	1.00	–	−8.51×10^−4^	8.84×10^−5^
H_2_O + 100 ng hCA II	hAQP5 + 10 ng hCA II	−9.40×10^−4^	2.84×10^−4^	4.68	0.0303	✓	−0.00183	−5.25×10^−5^
hAQP5 +100 ng hCA II	H_2_O + Tris	0.00183	2.57×10^−4^	10.0	3.68×10^−8^	✓	0.00102	0.00263
hAQP5 +100 ng hCA II	hAQP5 + Tris	8.79×10^−4^	2.41×10^−4^	5.15	0.0114	✓	1.25×10^−4^	0.00163
hAQP5 +100 ng hCA II	H_2_O + 1 ng hCA II	0.00166	2.57×10^−4^	9.11	4.03×10^−7^	✓	8.53×10^−4^	0.00246
hAQP5 +100 ng hCA II	hAQP5 + 1 ng hCA II	7.28×10^−4^	2.18×10^−4^	4.72	0.0280	✓	4.63×10^−5^	0.00141
hAQP5 +100 ng hCA II	H_2_O + 10 ng hCA II	0.00162	2.41×10^−4^	9.52	1.21×10^−7^	✓	8.70×10^−4^	0.00238
hAQP5 +100 ng hCA II	hAQP5 + 10 ng hCA II	6.68×10^−4^	2.49×10^−4^	3.80	0.144	–	−1.08×10^−4^	0.00144
hAQP5 +100 ng hCA II	H_2_O + 100 ng hCA II	0.00161	2.68×10^−4^	8.48	2.16×10^−6^	–	7.70×10^−4^	0.00244

*Notes*: Threshold for significance a = 0.05. Where the displayed P‐values is 0.00, this indicates a values less than <2.22×10^−308^. 2.22×10^−308^ is the smallest possible value for double type data that a 64‐bit system is able to distinguish.

Abbreviations: LCL, lower confidence limit; mean diff., mean difference for each comparison; UCL, upper confidence limit

**Table A6A:** Statistics summary for Fig. 6*A*: (i) descriptive statistics for data presented in the figure panel, initial pH_i_; (ii) overall one‐way ANOVA results; (iii) Tukey's means comparison analysis

i. Descriptive statistics				
	n	Mean	SD	SEM
H_2_O + Tris	8	7.27	0.0486	0.0172
hAQP5 + Tris	8	7.31	0.0512	0.0181
H_2_O + 1 ng hCA II	8	7.30	0.0614	0.0217
hAQP5 + 1 ng hCA II	8	7.32	0.0512	0.0181
H_2_O + 10 ng hCA II	8	7.26	0.0452	0.0160
hAQP5 + 10 ng hCA II	8	7.31	0.0352	0.0124
H_2_O + 100 ng hCA II	8	7.26	0.0614	0.0217
hAQP5 + 100 ng hCA II	8	7.29	0.0593	0.0210

ii. Overall ANOVA					
Variation	DF	Sum of squares	Mean square	*F*‐value	*P*‐value
Between groups	7	0.0324	0.00462	1.68	0.131
Within groups	56	0.154	0.00274		
Total	63	0.186			

iii. Tukey's test							
Condition A	Condition B	Mean diff.	*Q*‐value	*P*‐value	Sig.	LCL	UCL
hAQP5 + Tris	H_2_O + Tris	0.0440	2.37	0.701	–	−0.0385	0.126
H_2_O + 1 ng hCA II	H_2_O + Tris	0.0264	1.43	0.971	–	−0.0561	0.109
H_2_O + 1 ng hCA II	hAQP5 + Tris	−0.0176	0.948	0.997	–	−0.100	0.0649
hAQP5 + 1 ng hCA II	H_2_O + Tris	0.0515	2.78	0.512	–	−0.0309	0.134
hAQP5 + 1 ng hCA II	hAQP5 + Tris	0.00759	0.410	1.000	–	−0.0749	0.0900
hAQP5 + 1 ng hCA II	H_2_O + 1 ng hCA II	0.0252	1.36	0.978	–	−0.0573	0.108
H_2_O + 10 ng hCA II	H_2_O + Tris	−0.00884	0.477	1.000	–	−0.0913	0.0736
H_2_O + 10 ng hCA II	hAQP5 + Tris	−0.0528	2.85	0.481	–	−0.135	0.0297
H_2_O + 10 ng hCA II	H_2_O + 1 ng hCA II	−0.0352	1.90	0.877	–	−0.118	0.0472
H_2_O + 10 ng hCA II	hAQP5 + 1 ng hCA II	−0.0604	3.26	0.309	–	−0.143	0.0221
hAQP5 + 10 ng hCA II	H_2_O + Tris	0.0439	2.37	0.703	–	−0.0386	0.126
hAQP5 + 10 ng hCA II	hAQP5 + Tris	−1.08×10^−4^	0.00586	1.00	–	−0.0826	0.0824
hAQP5 + 10 ng hCA II	H_2_O + 1 ng hCA II	0.0175	0.943	0.998	–	−0.0650	0.0999
hAQP5 + 10 ng hCA II	hAQP5 + 1 ng hCA II	−0.00770	0.416	1.000	–	−0.0902	0.0748
hAQP5 + 10 ng hCA II	H_2_O + 10ng hCA II	0.0527	2.84	0.484	–	−0.0298	0.135
H_2_O + 100 ng hCA II	H_2_O + Tris	−0.00531	0.287	1.000	–	−0.0878	0.0772
H_2_O + 100ng hCA II	hAQP5 + Tris	−0.0493	2.66	0.569	–	−0.132	0.0332
H_2_O + 100 ng hCA II	H_2_O + 1 ng hCA II	−0.0317	1.71	0.925	–	−0.114	0.0508
H_2_O + 100 ng hCA II	hAQP5 + 1 ng hCA II	−0.0569	3.07	0.385	–	−0.139	0.0256
H_2_O + 100ng hCA II	H_2_O + 10 ng hCA II	0.00353	0.191	1.000	–	−0.0789	0.0860
H_2_O + 100 ng hCA II	hAQP5 + 10 ng hCA II	−0.0492	2.65	0.572	–	−0.132	0.0333
hAQP5 +100 ng hCA II	H_2_O + Tris	0.0177	0.957	0.997	–	−0.0647	0.100
hAQP5 +100 ng hCA II	hAQP5 + Tris	−0.0262	1.42	0.972	–	−0.109	0.0562
hAQP5 +100 ng hCA II	H_2_O + 1 ng hCA II	−0.00866	0.468	1.000	–	−0.0911	0.0738
hAQP5 +100 ng hCA II	hAQP5 + 1 ng hCA II	−0.0338	1.83	0.898	–	−0.116	0.0486
hAQP5 +100 ng hCA II	H_2_O + 10 ng hCA II	0.0266	1.43	0.970	–	−0.0559	0.109
hAQP5 +100 ng hCA II	hAQP5 + 10 ng hCA II	−0.0261	1.41	0.973	–	−0.109	0.0563
hAQP5 +100 ng hCA II	H_2_O + 100 ng hCA II	0.0230	1.24	0.987	–	−0.0594	0.105

*Notes*: Threshold for significance a = 0.05. SEM = 0.0262.

Abbreviations: DF, degrees of freedom; LCL, lower confidence limit; mean diff., mean difference for each comparison; UCL, upper confidence limit.

**Table A6B:** Statistics summary for Fig. 6*B*: (i) descriptive statistics for data presented in the figure panel, ΔpH_i_; (ii) overall one‐way ANOVA results; (iii) Tukey's means comparison analysis

i. Descriptive statistics				
	*n*	Mean	SD	SEM
H_2_O + Tris	8	0.251	0.0220	0.00778
hAQP5 + Tris	8	0.257	0.0253	0.00894
H_2_O + 1 ng hCA II	8	0.252	0.0296	0.0105
hAQP5 + 1 ng hCA II	8	0.274	0.0234	0.00828
H_2_O + 10 ng hCA II	8	0.264	0.0209	0.00740
hAQP5 + 10 ng hCA II	8	0.292	0.0238	0.00841
H_2_O + 100 ng hCA II	8	0.252	0.0254	0.00898
hAQP5 + 100 ng hCA II	8	0.278	0.0422	0.0149

ii. Overall ANOVA table					
Variation	DF	Sum of squares	Mean square	*F*‐value	*P*‐value
Between groups	7	0.0125	0.00178	2.39	0.0329
Within groups	56	0.0419	7.47×10^−4^		
Total	63	0.0543			

iii. Tukey's test							
Condition A	Condition B	Mean diff.	*Q*‐value	*P*‐value	Sig.	LCL	UCL
hAQP5 + Tris	H_2_O + Tris	0.00615	0.636	1.000	–	−0.0369	0.0492
H_2_O + 1 ng hCA II	H_2_O + Tris	0.00173	0.179	1.000	–	−0.0413	0.0448
H_2_O + 1 ng hCA II	hAQP5 + Tris	−0.00442	0.457	1.000	–	−0.0475	0.0386
hAQP5 + 1 ng hCA II	H_2_O + Tris	0.0234	2.42	0.679	–	−0.0196	0.0664
hAQP5 + 1 ng hCA II	hAQP5 + Tris	0.0173	1.79	0.909	–	−0.0258	0.0603
hAQP5 + 1 ng hCA II	H_2_O + 1 ng hCA II	0.0217	2.24	0.757	–	−0.0214	0.0647
H_2_O + 10 ng hCA II	H_2_O + Tris	0.0129	1.34	0.980	–	−0.0301	0.0560
H_2_O + 10 ng hCA II	hAQP5 + Tris	0.00677	0.700	1.000	–	−0.0363	0.0498
H_2_O + 10 ng hCA II	H_2_O + 1 ng hCA II	0.0112	1.16	0.991	–	−0.0319	0.0542
H_2_O + 10 ng hCA II	hAQP5 + 1 ng hCA II	−0.0105	1.09	0.994	–	−0.0535	0.0325
hAQP5 + 10 ng hCA II	H_2_O + Tris	0.0409	4.23	0.0737	–	−0.00212	0.0840
hAQP5 + 10 ng hCA II	hAQP5 + Tris	0.0348	3.60	0.199	–	−0.00828	0.0778
hAQP5 + 10 ng hCA II	H_2_O + 1 ng hCA II	0.0392	4.05	0.0995	–	−0.00386	0.0822
hAQP5 + 10 ng hCA II	hAQP5 + 1 ng hCA II	0.0175	1.81	0.902	–	−0.0255	0.0605
hAQP5 + 10 ng hCA II	H_2_O + 10ng hCA II	0.0280	2.90	0.460	–	−0.0150	0.0710
H_2_O + 100 ng hCA II	H_2_O + Tris	0.00154	0.160	1.000	–	−0.0415	0.0446
H_2_O + 100ng hCA II	hAQP5 + Tris	−0.00461	0.477	1.000	–	−0.0476	0.0384
H_2_O + 100 ng hCA II	H_2_O + 1 ng hCA II	−1.90×10^−4^	0.0197	1.00	–	−0.0432	0.0428
H_2_O + 100 ng hCA II	hAQP5 + 1ng hCA II	−0.0219	2.26	0.748	–	−0.0649	0.0212
H_2_O + 100ng hCA II	H_2_O + 10ng hCA II	−0.0114	1.18	0.990	–	−0.0544	0.0317
H_2_O + 100 ng hCA II	hAQP5 + 10 ng hCA II	−0.0394	4.07	0.0963	–	−0.0824	0.00367
hAQP5 +100 ng hCA II	H_2_O + Tris	0.0276	2.85	0.480	–	−0.0155	0.0706
hAQP5 +100 ng hCA II	hAQP5 + Tris	0.0214	2.22	0.767	–	−0.0216	0.0645
hAQP5 +100 ng hCA II	H_2_O + 1 ng hCA II	0.0258	2.67	0.563	–	−0.0172	0.0689
hAQP5 +100 ng hCA II	hAQP5 + 1 ng hCA II	0.00417	0.431	1.000	–	−0.0389	0.0472
hAQP5 +100 ng hCA II	H_2_O + 10 ng hCA II	0.0147	1.52	0.960	–	−0.0284	0.0577
hAQP5 +100 ng hCA II	hAQP5 + 10 ng hCA II	−0.0133	1.38	0.976	–	−0.0564	0.0297
hAQP5 +100 ng hCA II	H_2_O + 100 ng hCA II	0.0260	2.69	0.554	–	−0.0170	0.0691

*Notes*: Threshold for significance a = 0.05. SEM = 0.0137.

Abbreviations: DF, degrees of freedom; LCL, lower confidence limit; mean diff., mean difference for each comparison; UCL, upper confidence limit.

**Table A6C:** Statistics summary for Fig. 6*C*: (i) descriptive statistics for data presented in the figure panel, β_i_. (ii) overall one‐way ANOVA results; (iii) Tukey's means comparison analysis

i. Descriptive statistics				
	*n*	Mean	SD	SEM
H_2_O + Tris	8	13.4	2.20	0.779
hAQP5 + Tris	8	14.2	2.14	0.758
H_2_O + 1 ng hCA II	8	14.1	2.07	0.731
hAQP5 + 1 ng hCA II	8	12.9	1.21	0.427
H_2_O + 10 ng hCA II	8	12.1	2.03	0.718
hAQP5 + 10 ng hCA II	8	11.5	1.23	0.435
H_2_O + 100 ng hCA II	8	13.1	2.26	0.799
hAQP5 + 100 ng hCA II	8	11.8	1.57	0.556

ii. Overall ANOVA table					
Variation	DF	Sum of squares	Mean square	*F*‐value	*P*‐value
Between groups	7	57.6	8.22	2.32	0.0379
Within groups	56	199	3.55		
Total	63	256			

iii. Tukey's test							
Condition A	Condition B	Mean diff.	*Q*‐value	*P*‐value	Sig.	LCL	UCL
hAQP5 + Tris	H_2_O + Tris	0.873	1.31	0.982	–	−2.09	3.84
H_2_O + 1 ng hCA II	H_2_O + Tris	0.699	1.05	0.995	–	−2.27	3.67
H_2_O + 1 ng hCA II	hAQP5 + Tris	−0.173	0.260	1.000	–	−3.14	2.79
hAQP5 + 1 ng hCA II	H_2_O + Tris	−0.414	0.621	1.000	–	−3.38	2.55
hAQP5 + 1 ng hCA II	hAQP5 + Tris	−1.29	1.93	0.869	–	−4.25	1.68
hAQP5 + 1 ng hCA II	H_2_O + 1 ng hCA II	−1.11	1.67	0.934	–	−4.08	1.85
H_2_O + 10 ng hCA II	H_2_O + Tris	−1.27	1.91	0.876	–	−4.24	1.70
H_2_O + 10 ng hCA II	hAQP5 + Tris	−2.14	3.22	0.326	–	−5.11	0.824
H_2_O + 10 ng hCA II	H_2_O + 1 ng hCA II	−1.97	2.96	0.434	–	−4.93	0.997
H_2_O + 10 ng hCA II	hAQP5 + 1 ng hCA II	−0.856	1.28	0.984	–	−3.82	2.11
hAQP5 + 10 ng hCA II	H_2_O + Tris	−1.87	2.80	0.504	–	−4.83	1.10
hAQP5 + 10 ng hCA II	hAQP5 + Tris	−2.74	4.11	0.0905	–	−5.70	0.227
hAQP5 + 10 ng hCA II	H_2_O + 1 ng hCA II	−2.57	3.85	0.137	–	−5.53	0.401
hAQP5 + 10 ng hCA II	hAQP5 + 1 ng hCA II	−1.45	2.18	0.782	–	−4.42	1.51
hAQP5 + 10 ng hCA II	H_2_O + 10ng hCA II	−0.597	0.895	0.998	–	−3.56	2.37
H_2_O + 100 ng hCA II	H_2_O + Tris	−0.261	0.392	1.000	–	−3.23	2.71
H_2_O + 100ng hCA II	hAQP5 + Tris	−1.13	1.70	0.928	–	−4.10	1.83
H_2_O + 100 ng hCA II	H_2_O + 1 ng hCA II	−0.960	1.44	0.970	–	−3.93	2.01
H_2_O + 100 ng hCA II	hAQP5 + 1ng hCA II	0.153	0.229	1.000	–	−2.81	3.12
H_2_O + 100ng hCA II	H_2_O + 10ng hCA II	1.01	1.51	0.960	–	−1.96	3.97
H_2_O + 100 ng hCA II	hAQP5 + 10 ng hCA II	1.60	2.41	0.685	–	−1.36	4.57
hAQP5 +100 ng hCA II	H_2_O + Tris	−1.55	2.33	0.720	–	−4.52	1.41
hAQP5 +100 ng hCA II	hAQP5 + Tris	−2.42	3.64	0.188	–	−5.39	0.542
hAQP5 +100 ng hCA II	H_2_O + 1 ng hCA II	−2.25	3.38	0.267	–	−5.22	0.715
hAQP5 +100 ng hCA II	hAQP5 + 1 ng hCA II	−1.14	1.71	0.926	–	−4.10	1.83
hAQP5 +100 ng hCA II	H_2_O + 10 ng hCA II	−0.282	0.424	1.000	–	−3.25	2.68
hAQP5 +100 ng hCA II	hAQP5 + 10 ng hCA II	0.314	0.472	1.000	–	−2.65	3.28
hAQP5 +100 ng hCA II	H_2_O + 100 ng hCA II	−1.29	1.94	0.867	–	−4.26	1.68

*Notes*: Threshold for significance a = 0.05. SEM = 0.942.

Abbreviations: DF, degrees of freedom; LCL, lower confidence limit; mean diff., mean difference for each comparison; UCL, upper confidence limit.

**Table A6D:** Statistics summary for Fig. 6*D*: (i) descriptive statistics for data presented in the figure panel, *P*_f_; (ii) overall one‐way ANOVA results; (iii) Tukey's means comparison analysis

i. Descriptive statistics				
	*n*	Mean	SD	SEM
H_2_O + Tris	8	1.60×10^−4^	9.95×10^−5^	3.52×10^−5^
hAQP5 + Tris	8	0.00373	4.41×10^−4^	1.56×10^−4^
H_2_O + 1 ng hCA II	8	1.90×10^−4^	1.10×10^−4^	3.90×10^−5^
hAQP5 + 1 ng hCA II	8	0.00376	4.48×10^−4^	1.58×10^−4^
H_2_O + 10 ng hCA II	8	2.35×10^−4^	2.01×10^−4^	7.11×10^−5^
hAQP5 + 10 ng hCA II	8	0.00388	5.32×10^−4^	1.88×10^−4^
H_2_O + 100 ng hCA II	8	1.82×10^−4^	1.50×10^−4^	5.31×10^−5^
hAQP5 + 100 ng hCA II	8	0.00378	3.80×10^−4^	1.34×10^−4^

ii. Overall ANOVA table					
Variation	DF	Sum of squares	Mean square	*F*‐value	*P*‐value
Between groups	7	2.07×10^−4^	2.96×10^−5^	261	2.53×10^−4^
Within groups	56	6.35×10^−6^	1.13×10^−7^		
Total	63	2.13×10^−4^			

iii. Tukey's test							
Condition A	Condition B	Mean diff.	*Q*‐value	*P*‐value	Sig.	LCL	UCL
hAQP5 + Tris	H_2_O + Tris	0.00357	30.0	6.86×10^−8^	✓	0.00304	0.00410
H_2_O + 1 ng hCA II	H_2_O + Tris	3.02×10^−5^	0.254	1.00	–	−5.00×10^−4^	5.60×10^−4^
H_2_O + 1 ng hCA II	hAQP5 + Tris	−0.00354	29.8	8.93×10^−7^	✓	−0.00407	−0.00301
hAQP5 + 1 ng hCA II	H_2_O + Tris	0.00360	30.2	1.87×10^−6^	✓	0.00307	0.00413
hAQP5 + 1 ng hCA II	hAQP5 + Tris	2.33×10^−5^	0.196	1.00	–	−5.07×10^−4^	5.53×10^−4^
hAQP5 + 1 ng hCA II	H_2_O + 1ng hCA II	0.00357	30.0	2.79×10^−7^	✓	0.00304	0.00410
H_2_O + 10 ng hCA II	H_2_O + Tris	7.45×10^−5^	0.626	1.00	–	−4.56×10^−4^	6.05×10^−4^
H_2_O + 10 ng hCA II	hAQP5 + Tris	−0.00350	29.4	8.76×10^−7^	✓	−0.00403	−0.00297
H_2_O + 10 ng hCA II	H_2_O + 1 ng hCA II	4.43×10^−5^	0.372	1.00	–	−4.86×10^−4^	5.74×10^−4^
H_2_O + 10 ng hCA II	hAQP5 + 1 ng hCA II	−0.00352	29.6	8.85×10^−7^	✓	−0.00405	−0.00299
hAQP5 + 10 ng hCA II	H_2_O + Tris	0.00372	31.2	7.30×10^−8^	✓	0.00319	0.00425
hAQP5 + 10 ng hCA II	hAQP5 + Tris	1.46×10^−4^	1.23	0.988	–	−3.84×10^−4^	6.77×10^−4^
hAQP5 + 10 ng hCA II	H_2_O + 1 ng hCA II	0.00369	31.0	7.23×10^−8^	✓	0.00316	0.00422
hAQP5 + 10 ng hCA II	hAQP5 + 1 ng hCA II	1.23×10^−4^	1.03	0.996	–	^−4^.07×10^−4^	6.53×10^−4^
hAQP5 + 10 ng hCA II	H_2_O + 10ng hCA II	0.00364	30.6	7.16×10^−8^	✓	0.00311	0.00418
H_2_O + 100 ng hCA II	H_2_O + Tris	2.18×10^−5^	0.183	1.000	–	−5.08×10^−4^	5.52×10^−4^
H_2_O + 100ng hCA II	hAQP5 + Tris	−0.00355	29.8	8.96×10^−7^	✓	−0.00408	−0.00302
H_2_O + 100 ng hCA II	H_2_O + 1 ng hCA II	−8.37×10^−6^	0.0703	1.00	–	−5.38×10^−4^	5.22×10^−4^
H_2_O + 100 ng hCA II	hAQP5 + 1 ng hCA II	−0.00357	30.0	6.86×10^−8^	✓	−0.00410	−0.00304
H_2_O + 100ng hCA II	H_2_O + 10 ng hCA II	−5.27×10^−5^	0.442	1.00	–	−5.83×10^−4^	4.77×10^−4^
H_2_O + 100 ng hCA II	hAQP5 + 10 ng hCA II	−0.00370	31.1	7.25×10^−8^	✓	−0.00423	−0.00317
hAQP5 +100 ng hCA II	H_2_O + Tris	0.00362	30.4	7.08×10^−8^	✓	0.00309	0.00415
hAQP5 +100 ng hCA II	hAQP5 + Tris	4.36×10^−5^	0.366	1.00	–	−4.87×10^−4^	5.74×10^−4^
hAQP5 +100 ng hCA II	H_2_O + 1 ng hCA II	0.00359	30.1	1.86×10^−6^	✓	0.00306	0.00412
hAQP5 +100 ng hCA II	hAQP5 + 1 ng hCA II	2.03×10^−5^	0.170	1.00	–	−5.10×10^−4^	5.50×10^−4^
hAQP5 +100 ng hCA II	H_2_O + 10 ng hCA II	0.00354	29.7	8.93×10^−7^	✓	0.00301	0.00407
hAQP5 +100 ng hCA II	hAQP5 + 10 ng hCA II	−1.03×10^−4^	0.864	0.999	–	−6.33×10^−4^	4.27×10^−4^
hAQP5 +100 ng hCA II	H_2_O + 100 ng hCA II	0.00359	30.2	1.87×10^−6^	✓	0.00306	0.00412

*Notes*: Threshold for significance a = 0.05. SEM = 1.68 × 10^−4^.

Abbreviations: DF, degrees of freedom; LCL, lower confidence limit; mean diff., mean difference for each comparison; UCL, upper confidence limit.

**Table A8A:** Statistics summary for Fig. 8*A*: (i) descriptive statistics for data presented in the figure panel, ∆pH_S_ upon CO_2_ addition; (ii) overall one‐way ANOVA results; (iii) Tukey's means comparison analysis

i. Descriptive statistics				
	*n*	Mean	SD	SEM
H_2_O + Tris	6	0.235	0.235	0.0112
hAQP5 + Tris	6	0.236	0.236	0.00784
H_2_O + 1ng hCA II	6	0.324	0.324	0.0196
hAQP5 + 1ng hCA II	6	0.347	0.347	0.0230

ii. Overall ANOVA table					
Variation	DF	Sum of squares	Mean square	*F*‐value	*P*‐value
Between groups	3	0.0614	0.0205	12.4	8.35×10^−5^
Within groups	20	0.0330	0.00165		
Total	23	0.0944			

iii. Tukey's test							
Condition A	Condition B	Mean diff.	*Q*‐value	*P*‐value	Sig.	LCL	UCL
hAQP5 + Tris	H_2_O + Tris	0.00134	0.0810	1.000	–	−0.0643	0.0670
H_2_O + 1 ng hCA II	H_2_O + Tris	0.0889	5.36	0.00582	✓	0.0232	0.155
H_2_O + 1 ng hCA II	hAQP5 + Tris	0.0875	5.28	0.00663	✓	0.0219	0.153
hAQP5 + 1 ng hCA II	H_2_O + Tris	0.112	6.76	6.13×10^−4^	✓	0.0464	0.178
hAQP5 + 1 ng hCA II	hAQP5 + Tris	0.111	6.68	6.99×10^−4^	✓	0.0451	0.176
hAQP5 + 1 ng hCA II	H_2_O + 1 ng hCA II	0.0232	1.40	0.757	–	−0.0425	0.0888

*Notes*: Threshold for significance a = 0.05. SEM = 0.0235.

Abbreviations: DF, degrees of freedom; LCL, lower confidence limit; mean diff., mean difference for each comparison; UCL, upper confidence limit.

**Table A8B:** Statistics summary for Fig. 8*B*: (i) descriptive statistics for data presented in the figure panel, ∆pH_S_ upon CO_2_ removal; (ii) overall one‐way ANOVA results; (iii) Tukey's means comparison analysis

i. Descriptive statistics				
	*n*	Mean	SD	SEM
H_2_O + Tris	6	−0.335	0.0523	0.0213
hAQP5 + Tris	6	−0.341	0.0314	0.0128
H_2_O + 1 ng hCA II	6	−0.418	0.0546	0.0223
hAQP5 + 1 ng hCA II	6	−0.450	0.0531	0.0217

ii. Overall ANOVA table					
Variation	DF	Sum of squares	Mean square	*F*‐value	*P*‐value
Between groups	3	0.0590	0.0197	8.26	9.03×10^−4^
Within groups	20	0.0476	0.00238		
Total	23	0.107			

iii. Tukey's test							
Condition A	Condition B	Mean diff.	*Q*‐value	*P*‐value	Sig.	LCL	UCL
hAQP5 + Tris	H_2_O + Tris	−0.00633	0.318	0.996	–	−0.0852	0.0725
H_2_O + 1 ng hCA II	H_2_O + Tris	−0.0836	4.20	0.0352	✓	−0.162	−0.00478
H_2_O + 1 ng hCA II	hAQP5 + Tris	−0.0773	3.88	0.0560	–	−0.156	0.00156
hAQP5 + 1 ng hCA II	H_2_O + Tris	−0.116	5.80	0.00287	✓	−0.194	−0.0367
hAQP5 + 1 ng hCA II	hAQP5 + Tris	−0.109	5.48	0.00478	✓	−0.188	−0.0304
hAQP5 + 1 ng hCA II	H_2_O + 1 ng hCA II	−0.0319	1.60	0.674	–	−0.111	0.0469

*Notes*: Threshold for significance a = 0.05. SEM = 0.0282.

Abbreviations: DF, degrees of freedom; LCL, lower confidence limit; mean diff., mean difference for each comparison; UCL, upper confidence limit.

**Table A8C:** Statistics summary for Fig. 8*C*: (i) descriptive statistics for data presented in the figure panel, (dpH_i_/dt)_Max_ upon CO_2_ addition; (ii) overall one‐way ANOVA results; (iii) Tukey's means comparison analysis

i. Descriptive statistics				
	*n*	Mean	SD	SEM
H_2_O + Tris	6	−0.00250	7.09×10^−4^	2.89×10^−4^
hAQP5 + Tris	6	−0.00451	0.00110	4.48×10^−4^
H_2_O + 1 ng hCA II	6	−0.00742	9.98×10^−4^	4.08×10^−4^
hAQP5 + 1 ng hCA II	6	−0.00670	0.00165	6.73×10^−4^

ii. Overall ANOVA table					
Variation	DF	Sum of squares	Mean square	*F*‐value	*P*‐value
Between groups	3	8.95×10^−5^	2.98×10^−5^	22.0	1.50×10^−6^
Total	23	1.17×10^−4^			

iii. Tukey's test							
Condition A	Condition B	Mean diff.	*Q*‐value	*P*‐value	Sig.	LCL	UCL
hAQP5 + Tris	H_2_O + Tris	−0.00201	4.24	0.0331	✓	−0.00389	−1.33×10^−4^
H_2_O + 1 ng hCA II	H_2_O + Tris	−0.00492	10.4	2.42×10^−6^	✓	−0.00680	−0.00304
H_2_O + 1 ng hCA II	hAQP5 + Tris	−0.00291	6.12	0.00171	✓	−0.00479	−0.00103
hAQP5 + 1 ng hCA II	H_2_O + Tris	−0.00420	8.84	2.33×10^−5^	✓	−0.00608	−0.00232
hAQP5 + 1 ng hCA II	hAQP5 + Tris	−0.00218	4.60	0.0192	✓	−0.00407	−3.05×10^−4^
hAQP5 + 1 ng hCA II	H_2_O + 1 ng hCA II	7.23×10^−4^	1.52	0.707	–	−0.00116	0.00260

*Notes*: Threshold for significance a = 0.05. SEM = 6.72×10^−4^.

Abbreviations: DF, degrees of freedom; LCL, lower confidence limit; mean diff., mean difference for each comparison; UCL, upper confidence limit.

**Table A8D:** Statistics summary for Fig. 8*D*: (i) descriptive statistics for data presented in the figure panel, (dpH_i_/dt)_Max_ upon CO_2_ removal; (ii) overall one‐way ANOVA results; (iii) Tukey's means comparison analysis

i. Descriptive statistics				
	*n*	Mean	SD	SEM
H_2_O + Tris	6	0.00161	6.45×10^−4^	2.63×10^−4^
hAQP5 + Tris	6	0.00229	5.65×10^−4^	2.31×10^−4^
H_2_O + 1ng hCA II	6	0.00498	9.28×10^−4^	3.79×10^−4^
hAQP5 + 1ng hCA II	6	0.00406	0.00129	5.25×10^−4^

ii. Overall ANOVA table					
Variation	DF	Sum of squares	Mean square	*F*‐value	*P*‐value
Between groups	3	4.36×10^−5^	1.45×10^−5^	17.9	6.92×10^−7^
Within groups	20	1.62×10^−5^	8.12×10^−7^		
Total	23	5.99×10^−5^			

iii. Tukey's test							
Condition A	Condition B	Mean diff.	*Q*‐value	*P*‐value	Sig.	LCL	UCL
hAQP5 + Tris	H_2_O + Tris	6.78×10^−4^	1.84	0.571	–	−7.78×10^−4^	0.00213
H_2_O + 1 ng hCA II	H_2_O + Tris	0.00337	9.16	1.42×10^−5^	✓	0.00192	0.00483
H_2_O + 1 ng hCA II	hAQP5 + Tris	0.00269	7.32	2.49×10^−4^	✓	0.00124	0.00415
hAQP5 + 1 ng hCA II	H_2_O + Tris	0.00245	6.67	7.08×10^−4^	✓	9.97×10^−4^	0.00391
hAQP5 + 1 ng hCA II	hAQP5 + Tris	0.00178	4.82	0.0135	✓	3.19×10^−4^	0.00323
hAQP5 + 1 ng hCA II	H_2_O + 1 ng hCA II	−9.18×10^−4^	2.50	0.318	–	−0.00237	5.38×10^−4^

*Notes*: Threshold for significance a = 0.05. SEM = 5.20×10^−4^.

Abbreviations: DF, degrees of freedom; LCL, lower confidence limit; mean diff., mean difference for each comparison; UCL, upper confidence limit.

**Table A9A:** Statistics summary for Fig. 9*A*: (i) descriptive statistics for data presented in the figure panel, (dpH_S_/d_t_)_Max_ upon CO_2_ addition; (ii) overall one‐way ANOVA results. (iii) Tukey's means comparison analysis

i. Descriptive statistics				
	*n*	Mean	SD	SEM
H_2_O + Tris	6	−0.00502	0.00137	5.58×10^−4^
hAQP5 + Tris	6	−0.00541	0.00185	7.53×10^−4^
H_2_O + 1 ng hCA II	6	−0.00910	9.11×10^−4^	3.72×10^−4^
hAQP5 + 1 ng hCA II	6	−0.00843	0.00133	5.43×10^−4^

ii. Overall ANOVA table					
Variation	DF	Sum of squares	Mean square	*F*‐value	*P*‐value
Between groups	3	7.74×10^−5^	2.58×10^−5^	13.1	5.83×10^−5^
Within groups	20	3.94×10^−5^	1.97×10^−5^		
Total	23	1.17×10^−5^			panel

iii. Tukey's test							
Condition A	Condition B	Mean diff.	*Q*‐value	*P*‐value	Sig.	LCL	UCL
hAQP5 + Tris	H_2_O + Tris	−3.98×10^−4^	0.695	0.960	–	−0.00267	0.00187
H_2_O + 1 ng hCA II	H_2_O + Tris	−0.00408	7.13	3.38×10^−4^	✓	−0.00635	−0.00182
H_2_O + 1 ng hCA II	hAQP5 + Tris	−0.00369	6.43	0.00103	✓	−0.00595	−0.00142
hAQP5 + 1 ng hCA II	H_2_O + Tris	−0.00342	5.96	0.00221	✓	−0.00568	−0.00115
hAQP5 + 1 ng hCA II	hAQP5 + Tris	−0.00302	5.27	0.00674	✓	−0.00528	−7.50×10^−4^
hAQP5 + 1 ng hCA II	H_2_O + 1 ng hCA II	6.68×10^−4^	1.17	0.842	–	−0.00160	0.00294

*Notes*: Threshold for significance a = 0.05. SEM = 8.10 ×10^−4^.

Abbreviations: DF, degrees of freedom; LCL, lower confidence limit; mean diff., mean difference for each comparison; UCL, upper confidence limit.

**Table A9B:** Statistics summary for Fig. 9*B*: (i) descriptive statistics for data presented in the figure panel, (dpH_S_/dt)_Max_ upon CO_2_ removal; (ii) overall one‐way ANOVA results; (iii) Tukey's means comparison analysis.

i. Descriptive statistics				
	*n*	Mean	SD	SEM
H_2_O + Tris	6	0.00271	9.14×10^−4^	3.73×10^−4^
hAQP5 + Tris	6	0.00507	0.00180	7.34×10^−4^
H_2_O + 1 ng hCA II	6	0.00364	0.00127	5.18×10^−4^
hAQP5 + 1 ng hCA II	6	0.00479	0.00120	4.91×10^−4^

ii. Overall ANOVA table					
Variation	DF	Sum of squares	Mean square	*F*‐value	*P*‐value
Between groups	3	2.13×10^−5^	7.12×10^−6^	3.99	0.0222
Within groups	20	3.56×10^−5^	1.78×10^−6^		
Total	23	5.70×10^−5^			

iii. Tukey's test							
Condition A	Condition B	Mean diff.	*Q*‐value	*P*‐value	Sig.	LCL	UCL
hAQP5 + Tris	H_2_O + Tris	0.00236	4.33	0.0288	✓	2.04×10^−4^	0.00452
H_2_O + 1 ng hCA II	H_2_O + Tris	9.36×10^−4^	1.72	0.625	–	−0.00122	0.00309
H_2_O + 1 ng hCA II	hAQP5 + Tris	−0.00143	2.62	0.281	–	−0.00358	7.32×10^−4^
hAQP5 + 1 ng hCA II	H_2_O + Tris	0.00209	3.83	0.0604	–	−7.19×10^−5^	0.00424
hAQP5 + 1 ng hCA II	hAQP5 + Tris	−2.76×10^−4^	0.506	0.984	–	−0.00243	0.00188
hAQP5 + 1 ng hCA II	H_2_O + 1 ng hCA II	0.00115	2.11	0.461	–	−0.00101	0.00331

*Notes*: Threshold for significance a = 0.05. SEM = 7.71 ×10^−4^.

Abbreviations: DF, degrees of freedom; LCL, lower confidence limit; mean diff., mean difference for each comparison; UCL, upper confidence limit.

**Table A10A:** Statistics summary for Fig. 10*A*: (i) descriptive statistics for data presented in the figure panel, initial pH_i_ values; (ii) overall one‐way ANOVA results; (iii) Tukey's means comparison analysis

i. Descriptive statistics				
	*n*	Mean	SD	SEM
H_2_O + Tris	6	7.29	0.0530	0.0216
hAQP5 + Tris	6	7.32	0.0741	0.0303
H_2_O + 1 ng hCA II	6	7.28	0.0685	0.0280
hAQP5 + 1 ng hCA II	6	7.31	0.0441	0.0180

ii. Overall ANOVA table					
Variation	DF	Sum of squares	Mean square	*F*‐value	*P*‐value
Between groups	3	0.00556	0.00185	0.497	0.689
Within groups	20	0.0747	0.00373		
Total	23	0.0802			

iii. Tukey's test							
Condition A	Condition B	Mean diff.	*Q*‐value	*P*‐value	Sig.	LCL	UCL
hAQP5 + Tris	H_2_O + Tris	0.0352	1.41	0.752	–	−0.0635	0.134
H_2_O + 1 ng hCA II	H_2_O + Tris	−0.00157	0.0629	1.000	–	−0.100	0.0972
H_2_O + 1 ng hCA II	hAQP5 + Tris	−0.0368	1.47	0.727	–	−0.136	0.0620
hAQP5 + 1 ng hCA II	H_2_O + Tris	0.0203	0.813	0.939	–	−0.0785	0.119
hAQP5 + 1 ng hCA II	hAQP5 + Tris	−0.0149	0.599	0.974	–	−0.114	0.0838
hAQP5 + 1 ng hCA II	H_2_O + 1 ng hCA II	0.0218	0.876	0.925	–	−0.0769	0.121

*Notes*: Threshold for significance a = 0.05. SEM = 0.0353.

Abbreviations: DF, degrees of freedom; LCL, lower confidence limit; mean diff., mean difference for each comparison; UCL, upper confidence limit.

**Table A10B:** Statistics summary for Fig. 10*B*: (i) descriptive statistics for data presented in the figure, ΔpH_i_ values; (ii) overall one‐way ANOVA results; (iii) Tukey's means comparison analysis

i. Descriptive statistics				
	*n*	Mean	SD	SEM
H_2_O + Tris	6	−0.275	0.0274	0.0112
hAQP5 + Tris	6	−0.291	0.0117	0.00477
H_2_O + 1 ng hCA II	6	−0.275	0.0374	0.0153
hAQP5 + 1 ng hCA II	6	−0.301	0.0383	0.0157

ii. Overall ANOVA table					
Variation	DF	Sum of squares	Mean square	*F*‐value	*P*‐value
Between groups	3	0.00297	9.90×10^−4^	1.05	0.391
Within groups	20	0.0188	9.40×10^−4^		
Total	23	0.0218			

iii. Tukey's test							
Condition A	Condition B	Mean diff.	*Q*‐value	*P*‐value	Sig.	LCL	UCL
hAQP5 + Tris	H_2_O + Tris	−0.0161	1.29	0.798	–	−0.0334	0.0657
H_2_O + 1 ng hCA II	H_2_O + Tris	6.38×10^−4^	0.0510	1.000	–	−0.0502	0.0489
H_2_O + 1 ng hCA II	hAQP5 + Tris	0.0168	1.34	0.779	–	−0.0663	0.0327
hAQP5 + 1 ng hCA II	H_2_O + Tris	−0.0256	2.05	0.486	–	−0.0239	0.0752
hAQP5 + 1 ng hCA II	hAQP5 + Tris	−0.00948	0.758	0.949	–	−0.0401	0.0590
hAQP5 + 1 ng hCA II	H_2_O + 1ng hCA II	−0.0263	2.10	0.465	–	−0.0233	0.0758

*Notes*: Threshold for significance a = 0.05. SEM = 0.0177.

Abbreviations: DF, degrees of freedom; LCL, lower confidence limit; mean diff., mean difference for each comparison; UCL, upper confidence limit.

**Table A10C:** Statistics summary for Fig. 10*C*: (i) descriptive statistics for data presented in the figure panel, intrinsic buffering power (β_I_); (ii) overall one‐way ANOVA results; (iii) Tukey's means comparison analysis

i. Descriptive statistics				
	*n*	Mean	SD	SEM
H_2_O + Tris	6	12.0	2.10	0.855
hAQP5 + Tris	6	11.7	1.63	0.664
H_2_O + 1 ng hCA II	6	12.0	1.87	0.762
hAQP5 + 1 ng hCA II	6	10.8	1.43	0.586

ii. Overall ANOVA table					
Variation	DF	Sum of squares	Mean square	*F*‐value	*P*‐value
Between groups	3	5.77	1.92	0.612	0.615
Within groups	20	62.9	3.14		
Total	23	68.6			

iii. Tukey's test							
Condition A	Condition B	Mean diff.	*Q*‐value	*P*‐value	Sig.	LCL	UCL
hAQP5 + Tris	H_2_O + Tris	−0.265	0.366	0.994	–	−3.13	2.60
H_2_O + 1 ng hCA II	H_2_O + Tris	−0.0111	0.0154	1.000	–	−2.88	2.85
H_2_O + 1 ng hCA II	hAQP5 + Tris	0.253	0.350	0.994	–	−2.61	3.12
hAQP5 + 1 ng hCA II	H_2_O + Tris	−1.20	1.65	0.652	–	−4.06	1.67
hAQP5 + 1 ng hCA II	hAQP5 + Tris	−0.932	1.29	0.799	–	−3.80	1.93
hAQP5 + 1 ng hCA II	H_2_O + 1 ng hCA II	−1.19	1.64	0.659	–	−4.05	1.68

*Notes*: Threshold for significance a = 0.05. SEM = 1.02.

Abbreviations: DF, degrees of freedom; LCL, lower confidence limit; mean diff., mean difference for each comparison; UCL, upper confidence limit.

**Table A10D:** Statistics summary for Fig. 10*D*: (i) descriptive statistics for data presented in the figure panel, *P*_f_; (ii) overall one‐way ANOVA results; (iii) Tukey's means comparison analysis

i. Descriptive statistics				
	*n*	Mean	SD	SEM
H_2_O + Tris	8	1.91×10^−4^	2.19×10^−4^	7.74×10^−5^
hAQP5 + Tris	7	1.67×10^−4^	1.18×10^−4^	4.47×10^−5^
H_2_O + 1 ng hCA II	7	0.00389	3.34×10^−4^	1.26×10^−4^
hAQP5 + 1 ng hCA II	8	0.00391	4.82×10^−4^	1.70×10^−4^

ii. Overall ANOVA table					
Variation	DF	Sum of squares	Mean square	*F*‐value	*P*‐value
Between groups	3	1.04×10^−4^	3.46×10^−5^	331	7.92×10^−21^
Within groups	26	2.72×10^−6^	1.04×10^−7^		
Total	29	1.07×10^−4^			

iii. Tukey's test								
Condition A	Condition B	Mean diff.	SEM	*Q*‐value	*P*‐value	Sig.	LCL	UCL
H_2_O + 1 ng hCA II	H_2_O + Tris	−2.44×10^−5^	1.67×10^−4^	0.206	0.999	–	−4.83×10^−4^	4.34×10^−4^
hAQP5 + Tris	H_2_O + Tris	0.00370	1.67×10^−4^	31.3	2.10×10^−12^	✓	0.00324	0.00416
H_2_O + 1 ng hCA II	hAQP5 + Tris	0.00372	1.73×10^−4^	30.5	2.10×10^−12^	✓	0.00325	0.00420
hAQP5 + 1 ng hCA II	H_2_O + Tris	0.00372	1.62×10^−4^	32.6	2.10×10^−12^	✓	0.00328	0.00416
hAQP5 + 1 ng hCA II	H_2_O + 1 ng hCA II	0.00375	1.67×10^−4^	31.7	2.10×10^−12^	✓	0.00329	0.00420
hAQP5 + 1 ng hCA II	hAQP5 + Tris	2.32×10^−5^	1.67×10^−4^	0.196	0.999	–	−4.36×10^−4^	4.82×10^−4^

*Note*: Threshold for significance a = 0.05. SEM for Tukey's test.

Abbreviations: DF, degrees of freedom; LCL, lower confidence limit; mean diff., mean difference for each comparison; UCL, upper confidence limit.

**Table A11A:** Statistics summary for Fig. 11*A*: (i) descriptive statistics for data presented in the figure panel, which are the ∆pH_S_ upon CO_2_ addition data rearranged from Fig. 3*A* to juxtapose two hAQP5 absent bars and two heterologously expressed hAQP5 bars; (ii) overall one‐way ANOVA results for only data bars presented in Fig. 11*A* extracted from Fig. 3*A*; (iii) Tukey's means comparison analysis for all data presented in Fig. 11A; (iv) descriptive statistics for data presented in Fig. 11*A* to analyse ΔΔ_Base_ *vs*. ΔΔ_AQP5_ when adding 1 ng hCA II on the ‘trans’ side of the membrane to the pH_S_ measurement; (v) one‐way ANOVA results for ΔΔ_Base_ *vs*. ΔΔ_AQP5_ data presented in Fig. 11*A*; (vi) Tukey's means comparison analysis for ΔΔ_Base_ *vs*. ΔΔ_AQP5_ data presented in Fig. 11*A*

i. Descriptive statistics for ΔpH_S_ on CO_2_ addition				
	*n*	Mean	SD	SEM
H_2_O + Tris	8	0.0285	0.00895	0.00316
hAQP5 + Tris	8	0.0787	0.0181	0.00641
H_2_O + 1 ng hCA II	8	0.0411	0.0115	0.00406
hAQP5 + 1 ng hCA II	8	0.127	0.0246	0.00869

ii. Overall ANOVA table for ΔpH_S_ on CO_2_ addition					
Variation	DF	Sum of squares	Mean square	*F*‐value	*P*‐value
Between groups	3	0.0471	0.0157	54.8	7.63×10^−12^
Within groups	28	0.00802	2.86×10^−4^		
Total	31	0.0552			

iii. Tukey's test for ΔpH_S_ on CO_2_ addition								
Condition A	Condition B	Mean diff.	SEM	*Q*‐value	*P*‐value	Sig.	LCL	UCL
hAQP5 + Tris	H_2_O + Tris	0.0502	0.00846	8.39	1.25×10^−5^	✓	0.0271	0.0733
H_2_O + 1 ng hCA II	H_2_O + Tris	0.0126	0.00846	2.11	0.455	–	−0.0105	0.0357
H_2_O + 1 ng hCA II	hAQP5 + Tris	−0.0376	0.00846	6.28	7.00×10^−4^	✓	−0.0607	−0.0145
hAQP5 + 1 ng hCA II	H_2_O + Tris	0.0986	0.00846	16.5	0.00	✓	0.0755	0.122
hAQP5 + 1 ng hCA II	hAQP5 + Tris	0.0484	0.00846	8.09	2.20×10^−5^	✓	0.0253	0.0715
hAQP5 + 1 ng hCA II	H_2_O + 1 ng hCA II	0.0860	0.00846	14.4	6.50×10^−7^	✓	0.0629	0.109

iv. Descriptive statistics for ΔΔpH_S_ on CO_2_ addition				
	*n*	Mean	SD	SEM
ΔΔ_Base_	8	0.0126	0.0115	0.00406
ΔΔ_hAQP5_	8	0.0484	0.0246	0.00869

v. Overall ANOVA table for ΔΔpH_S_ on CO_2_ addition					
Variation	DF	Sum of squares	Mean square	*F*‐value	*P*‐value
Between groups	1	0.00513	0.00513	13.9	0.00223
Within groups	14	0.00516	3.68×10^−4^		
Total	15	0.0103			

vi. Tukey's test for ΔΔpH_S_ on CO_2_ addition								
Condition A	Condition B	Mean diff.						
	SEM	*Q*‐value	*P*‐value	Sig.	LCL	UCL		
ΔΔ_Base_	ΔΔ_AQP5_	0.0358	0.00960	5.28	0.00224	✓	0.0152	0.0564

*Notes*: Threshold for significance a = 0.05. SEM for Tukey's test.

Abbreviations: DF, degrees of freedom; LCL, lower confidence limit; mean diff., mean difference for each comparison; UCL, upper confidence limit.

**Table A11B:** Statistics summary for Fig. 11*B*: (i) descriptive statistics for data presented in Fig. 11*B*, which are the ∆pH_S_ upon CO_2_ removal data rearranged from Fig. 3*B* to juxtapose two hhAQP5 absent bars and two heterologously expressed hAQP5 bars; (ii) overall one‐way ANOVA results for only data bars presented in Fig. 11*B* extracted from Fig. 3*B*; (iii) Tukey's means comparison analysis for all data presented in Fig. 11*B*; (iv) descriptive statistics for data presented in Fig. 11*B* to analyse ΔΔ_Base_ *vs*. ΔΔ_AQP5_ when adding 1 ng hCA II on the ‘trans’ side of the membrane to the pH_S_ measurement; (v) one‐way ANOVA results for ΔΔ_Base_ *vs*. ΔΔ_AQP5_ data presented in Fig. 11*B*; (vi) Tukey's means comparison analysis for ΔΔ_Base_ *vs*. ΔΔ_AQP5_ data presented in Fig. 11*B*

i. Descriptive statistics for ΔpH_S_ on CO_2_ addition				
	n	Mean	SD	SEM
H_2_O + Tris	8	0.0285	0.00895	0.00316
hAQP5 + Tris	8	0.0787	0.0181	0.00641
H_2_O + 100 ng hCA II	8	0.0416	0.00948	0.00335
hAQP5 + 100 ng hCA II	8	0.160	0.0478	0.0169

ii. *Overall ANOVA table for ΔpH_S_ on CO_2_ addition*					
Variation	DF	Sum of squares	Mean square	*F*‐value	*P*‐value
Between groups	3	0.0845	0.0282	40.4	2.61×10^−10^
Within groups	28	0.0195	6.97×10^−4^		
Total	31	0.104			

iii. Tukey's test for ΔpH_S_ on CO_2_ addition								
Condition A	Condition B	Mean diff.	SEM	*Q*‐value	*P*‐value	Sig.	LCL	UCL
hAQP5 + Tris	H_2_O + Tris	0.0502	0.0132	5.38	0.00374	✓	0.0142	0.0862
H_2_O + 100 ng hCA II	H_2_O + Tris	0.0131	0.0132	1.41	0.754	–	−0.0229	0.0492
H_2_O + 100 ng hCA II	hAQP5 + Tris	−0.0371	0.0132	3.97	0.0419	✓	−0.0731	−0.00104
hAQP5 + 100 ng hCA II	H_2_O + Tris	0.132	0.0132	14.1	2.23×10^−7^	✓	0.0959	0.168
hAQP5 + 100 ng hCA II	hAQP5 + Tris	0.0817	0.0132	8.76	6.27×10^−6^	✓	0.0457	0.118
hAQP5 + 100 ng hCA II	H_2_O + 100 ng hCA II	0.119	0.0132	12.7	0.00	✓	0.0828	0.155

iv. Descriptive statistics for ΔΔpH_S_ on CO_2_ addition				
	*n*	Mean	SD	SEM
ΔΔ_Base_	8	0.0131	0.00948	0.00335
ΔΔ_hAQP5_	8	0.0817	0.0478	0.0169

v. Overall ANOVA table					
Variation	DF	Sum of squares	Mean square	*F*‐value	*P*‐value
Between groups	1	0.0188	0.0188	15.8	0.00137
Within groups	14	0.0166	0.00119		
Total	15	0.0355			

vi. Tukey's test								
Condition A	Condition B	Mean diff.	SEM	*Q*‐value	*P*‐value	Sig.	LCL	UCL
ΔΔ_Base_	ΔΔ_AQP5_	0.0686	0.0172	5.63	0.00137	✓	0.0316	0.106

*Notes*: Threshold for significance a = 0.05. SEM for Tukey's test.

Abbreviations: DF, degrees of freedom; LCL, lower confidence limit; mean diff., mean difference for each comparison; UCL, upper confidence limit.

**Table A12A:** Statistics summary for Fig. 12*A*: (i) descriptive statistics for data presented in Fig. 12*A* for comparison of select –(dpH_i_/dt)_Max_ bars extracted from Fig. 3*C* and Fig. 8*C* to analyse –(dpH_i_/dt)_Max_ upon CO_2_ addition in the absence or presence of bCA in bECF on the ‘trans’ side of the membrane to the pH_i_ measurement; (ii) overall one‐way ANOVA results for –(dpH_i_/dt)_Max_ data presented in Fig. 12*A*; (iii) Tukey's means comparison analysis for –(dpH_i_/dt)_Max_ was performed on the subset of data presented in Fig. 12*A*; (iv) descriptive statistics for data presented in Fig. 12*A* to analyse Δ_Base_ *vs*. Δ_AQP5_; (v) one‐way ANOVA results for Δ_Base_ *vs*. Δ_AQP5_ data presented in Fig. 12*A*; (vi) Tukey's means comparison analysis for ΔΔ_Base_ *vs*. ΔΔ_AQP5_ data presented in Fig. 12*A*

i. Descriptive statistics for –(dpH_i_/dt)_Max_ on CO_2_ addition				
	*n*	Mean	SD	SEM
H_2_O + Tris	8	−0.00126	2.08×10^−4^	7.35×10^−5^
H_2_O + Tris + bCA_bECF_	6	−0.00250	7.09×10^−4^	2.89×10^−4^
hAQP5 + Tris	8	−0.00122	1.57×10^−4^	5.55×10^−5^
hAQP5 + Tris+ bCA_bECF_	6	−0.00451	0.00110	4.48×10^−4^

ii. Overall ANOVA table for –(dpH_i_/dt)_Max_ on CO_2_ addition					
Variation	DF	Sum of squares	Mean square	*F*‐value	*P*‐value
Between groups	3	4.73×10^−5^	1.58×10^−5^	42.0	1.04×10^−9^
Within groups	24	9.00×10^−6^	3.75×10^−7^		
Total	27	5.63×10^−5^			

iii. Tukey's test for –(dpH_i_/dt)_Max_ on CO_2_ addition								
Condition A	Condition B	Mean diff.	SEM	*Q*‐value	*P*‐value	Sig.	LCL	UCL
H_2_O + Tris + bCA_bECF_	H_2_O + Tris	−0.00124	3.31×10^−4^	5.30	0.00513	✓	−0.00215	−3.27×10^−4^
hAQP5 + Tris	H_2_O + Tris	3.53×10^−5^	3.06×10^−4^	0.163	0.999	–	−8.09×10^−4^	8.80×10^−4^
hAQP5 + Tris	H_2_O + Tris + bCA_bECF_	0.00127	3.31×10^−4^	5.45	0.00396	✓	3.62×10^−4^	0.00219
hAQP5 + Tris+ bCA_bECF_	H_2_O + Tris	−0.00325	3.31×10^−4^	13.9	4.43×10^−7^	✓	−0.00417	−0.00234
hAQP5 + Tris+ bCA_bECF_	H_2_O + Tris + bCA_bECF_	−0.00201	3.54×10^−4^	8.05	4.07×10^−5^	✓	−0.00299	−0.00104
hAQP5 + Tris+ bCA_bECF_	hAQP5 + Tris	−0.00329	3.31×10^−4^	14.1	4.47×10^−7^	✓	−0.00420	−0.00238

iv. Descriptive statistics for Δ_hAQP5_ *vs*. Δ_Base_ for –(dpH_i_/dt)_Max_ on CO_2_ addition				
	*n*	Mean	SD	SEM
Δ_Base_	6	−0.00271	7.09×10^−4^	2.89×10^−4^
Δ_AQP5_	6	−0.00467	0.00110	4.48×10^−4^

v. Overall ANOVA table					
Variation	DF	Sum of squares	Mean square	*F*‐value	*P*‐value
Between groups	1	1.16×10^−5^	1.16×10^−5^	13.6	0.00424
Within groups	10	8.53×10^−6^	8.53×10^−7^		
Total	11	2.01×10^−5^			

vi. Tukey's test								
Condition A	Condition B	Mean diff.	SEM	*Q*‐value	*P*‐value	Sig.	LCL	UCL
Δ_Base_	Δ_AQP5_	−0.00196	5.33×10^−4^	5.21	0.00424	✓	−0.00315	−7.75×10^−4^

*Note*: Threshold for significance a = 0.05. SEM for Tukey's test.

Abbreviations: DF, degrees of freedom; LCL, lower confidence limit; mean diff., mean difference for each comparison; UCL, upper confidence limit.

**Table A12B:** Statistics summary for Fig. 12*B*: (i) descriptive statistics for data presented in Fig. 12*B*, for comparison of select –(dpH_i_/dt)_Max_ bars extracted from Fig. 3*D* and Fig. 8*D* to analyse +(dpH_i_/dt)_Max_ upon CO_2_ removal in the absence or presence of bCA in bECF on the ‘trans’ side of the membrane to the pH_i_ measurement; (ii) overall one‐way ANOVA results for +(dpH_i_/dt)_Max_ data presented in Fig. 12*B*; (iii) Tukey's means comparison analysis for +(dpH_i_/dt)_Max_ data presented in Fig. 12*B*; (iv) descriptive statistics for data presented in Fig. 12*B* to analyse Δ_Base_ *vs*. Δ_AQP5_; (v) one‐way ANOVA results for Δ_Base_ *vs*. Δ_AQP5_ data presented in Fig. 12*B*; (vi) Tukey's means comparison analysis for ΔΔ_Base_ *vs*. ΔΔ_AQP5_ data presented in Fig. 12*B*

i. Descriptive statistics for +(dpH_i_/dt)_Max_ on CO_2_ removal				
	*n*	Mean	SD	SEM
H_2_O + Tris	8	8.35×10^−4^	1.34×10^−4^	4.74×10^−5^
H_2_O + Tris + bCA_bECF_	6	0.00161	6.45×10^−4^	2.63×10^−4^
hAQP5 + Tris	8	8.07×10^−4^	6.85×10^−5^	2.42×10^−5^
hAQP5 + Tris+ bCA_bECF_	6	0.00229	5.65×10^−4^	2.31×10^−4^

ii. Overall ANOVA table for +(dpH_i_/dt)_Max_ on CO_2_ removal					
Variation	DF	Sum of squares	Mean square	*F*‐value	*P*‐value
Between groups	3	1.01×10^−5^	3.35×10^−6^	21.0	6.82×10^−7^
Within groups	24	3.84×10^−6^	1.60×10^−7^		
Total	27	1.39×10^−5^			

iii. Tukey's test for +(dpH_i_/dt)_Max_ on CO_2_ removal								
Condition A	Condition B	Mean diff.	SEM	*Q*‐value	*P*‐value	Sig.	LCL	UCL
H_2_O + Tris + bCA_bECF_	H_2_O + Tris	7.72×10^−4^	2.16×10^−4^	5.06	0.00778	✓	1.76×10^−4^	0.00137
hAQP5 + Tris	H_2_O + Tris	−2.75×10^−5^	2.00×10^−4^	0.195	0.999	–	−5.79×10^−4^	5.24×10^−4^
hAQP5 + Tris	H_2_O + Tris + bCA_bECF_	−7.99×10^−4^	2.16×10^−4^	5.24	0.00572	✓	−0.00140	−2.04×10^−4^
hAQP5 + Tris+ bCA_bECF_	H_2_O + Tris	0.00145	2.16×10^−4^	9.50	3.43×10^−6^	✓	8.55×10^−4^	0.00205
hAQP5 + Tris+ bCA_bECF_	H_2_O + Tris + bCA_bECF_	6.78×10^−4^	2.31×10^−4^	4.16	0.0338	✓	4.15×10^−5^	0.00131
hAQP5 + Tris+ bCA_bECF_	hAQP5 + Tris	0.00148	2.16×10^−4^	9.68	2.54×10^−6^	✓	8.82×10^−4^	0.00207

iv. Descriptive statistics for Δ_AQP5_ *vs*. Δ_Base_ for –(dpH_i_/dt)_Max_ on CO_2_ removal				
	*n*	Mean	SD	SEM
Δ_Base_	6	7.72×10^−4^	6.45×10^−4^	2.63×10^−4^
Δ_AQP5_	6	0.00148	5.65×10^−4^	2.31×10^−4^

v. Overall ANOVA table					
Variation	DF	Sum of squares	Mean square	*F*‐value	*P*‐value
Between groups	1	1.49×10^−6^	1.49×10^−6^	4.06	0.0715
Within groups	10	3.68×10^−6^	3.68×10^−7^		
Total	11	5.17×10^−6^			

vi. Tukey's test								
Condition A	Condition B	Mean diff.	SEM	*Q*‐value	*P*‐value	Sig.	LCL	UCL
Δ_Base_	Δ_AQP5_	7.06×10^−4^	3.50×10^−4^	2.85	0.0715	✓	−7.43×10^−5^	0.00149

*Notes*: Threshold for significance a = 0.05. SEM for Tukey's test.

Abbreviations: DF, degrees of freedom; LCL, lower confidence limit; mean diff., mean difference for each comparison; UCL, upper confidence limit.

**Table A13:** Statistics summary for Fig. *A*1: (i) descriptive statistics for data presented in Fig. *A*1 for summarizing *V*_m_ values for oocytes used in Fig. 3; (ii) overall one‐way ANOVA results for *V*_m_ data presented in Fig. *A*1; (iii) Tukey's means comparison analysis for *V*_m_ data presented in Fig. *A*1

i. Descriptive statistics				
	*n*	Mean	SD	SEM
H_2_O + Tris	8	−57.8	6.77	2.39
hAQP5 + Tris	8	−45.5	2.90	1.02
hAQP5 + 1 ng hCA II	8	−51.8	8.44	2.98
H_2_O + 1 ng hCA II	8	−43.0	6.55	2.32
H_2_O + 10 ng hCA II	8	−55.1	7.14	2.52
hAQP5 + 10 ng hCA II	8	−44.9	7.05	2.49
H_2_O + 100 ng hCA II	8	−57.6	6.25	2.21
hAQP5 + 100 ng hCA II	8	−46.8	4.39	1.55

ii. Overall ANOVA table					
Variation	DF	Sum of squares	Mean square	*F*‐value	*P*‐value
Between groups	7.00	2.01×10^3^	287	7.01	5.30×10^−6^
Within groups	56.0	2.29×10^3^	40.9		
Total	63.0	4.30×10^3^			

iii. Tukey's test							
Condition A	Condition B	Mean diff.	*Q*‐value	*P*‐value	Sig.	LCL	UCL
hAQP5 + Tris	H_2_O + Tris	12.2	5.42	0.00738	✓	2.18	22.3
H_2_O + 1 ng hCA II	H_2_O + Tris	5.96	2.63	0.581	–	−4.11	16.0
H_2_O + 1 ng hCA II	hAQP5 + Tris	−6.29	2.78	0.513	–	−16.4	3.78
hAQP5 + 1 ng hCA II	H_2_O + Tris	14.7	6.51	6.06×10^−4^	✓	4.66	24.8
hAQP5 + 1 ng hCA II	hAQP5 + Tris	2.48	1.10	0.994	–	−7.59	12.6
hAQP5 + 1 ng hCA II	H_2_O + 1ng hCA II	8.77	3.88	0.132	–	−1.30	18.8
H_2_O + 10 ng hCA II	H_2_O + Tris	2.65	1.17	0.991	–	−7.43	12.7
H_2_O + 10 ng hCA II	hAQP5 + Tris	−9.60	4.25	0.0720	–	−19.7	0.467
H_2_O + 10 ng hCA II	H_2_O + 1ng hCA II	−3.31	1.46	0.967	–	−13.4	6.76
H_2_O + 10 ng hCA II	hAQP5 + 1ng hCA II	−12.1	5.34	0.00863	✓	−22.2	−2.01
hAQP5 + 10 ng hCA II	H_2_O + Tris	12.9	5.70	0.00396	✓	2.82	23.0
hAQP5 + 10 ng hCA II	hAQP5 + Tris	0.646	0.286	1.000	–	−9.43	10.7
hAQP5 + 10 ng hCA II	H_2_O + 1 ng hCA II	6.94	3.07	0.386	–	−3.13	17.0
hAQP5 + 10 ng hCA II	hAQP5 + 1 ng hCA II	−1.83	0.810	0.999	–	−11.9	8.24
hAQP5 + 10 ng hCA II	H_2_O + 10 ng hCA II	10.3	4.53	0.0433	✓	0.179	20.3
H_2_O + 100 ng hCA II	H_2_O + Tris	0.208	0.0921	1.00	–	−9.86	10.3
H_2_O + 100 ng hCA II	hAQP5 + Tris	−12.0	5.32	0.00897	✓	−22.1	−1.97
H_2_O + 100 ng hCA II	H_2_O + 1 ng hCA II	−5.75	2.54	0.624	–	−15.8	4.32
H_2_O + 100 ng hCA II	hAQP5 + 1 ng hCA II	−14.5	6.42	7.56×10^−4^	✓	−24.6	−4.45
H_2_O + 100 ng hCA II	H_2_O + 10 ng hCA II	−2.44	1.08	0.994	–	−12.5	7.63
H_2_O + 100 ng hCA II	hAQP5 + 10 ng hCA II	−12.7	5.61	0.00485	✓	−22.8	−2.62
hAQP5 +100 ng hCA II	H_2_O + Tris	10.9	4.84	0.0241	✓	0.877	21.0
hAQP5 +100 ng hCA II	hAQP5 + Tris	−1.30	0.576	1.000	–	−11.4	8.77
hAQP5 +100 ng hCA II	H_2_O + 1 ng hCA II	4.99	2.21	0.771	–	−5.08	15.1
hAQP5 +100 ng hCA II	hAQP5 + 1 ng hCA II	−3.78	1.67	0.934	–	−13.9	6.29
hAQP5 +100 ng hCA II	H_2_O + 10 ng hCA II	8.30	3.67	0.179	–	−1.77	18.4
hAQP5 +100 ng hCA II	hAQP5 + 10 ng hCA II	−1.95	0.861	0.999	–	−12.0	8.12
hAQP5 +100 ng hCA II	H_2_O + 100 ng hCA II	10.7	4.75	0.0288	✓	0.668	20.8

*Notes*: Threshold for significance a = 0.05. SEM for Tukey's test = 3.20.

Abbreviations: DF, degrees of freedom; LCL, lower confidence limit; mean diff., mean difference for each comparison; UCL, upper confidence limit.
